# ICAM1 initiates CTC cluster formation and trans-endothelial migration in lung metastasis of breast cancer

**DOI:** 10.1038/s41467-021-25189-z

**Published:** 2021-08-11

**Authors:** Rokana Taftaf, Xia Liu, Salendra Singh, Yuzhi Jia, Nurmaa K. Dashzeveg, Andrew D. Hoffmann, Lamiaa El-Shennawy, Erika K. Ramos, Valery Adorno-Cruz, Emma J. Schuster, David Scholten, Dhwani Patel, Youbin Zhang, Andrew A. Davis, Carolina Reduzzi, Yue Cao, Paolo D’Amico, Yang Shen, Massimo Cristofanilli, William A. Muller, Vinay Varadan, Huiping Liu

**Affiliations:** 1 Department of Pharmacology, Northwestern University Feinberg School of Medicine, Chicago, IL USA; 2 Driskill Graduate Program in Life Sciences, Northwestern University Feinberg School of Medicine, Chicago, IL USA; 3 Department of Toxicology and Cancer Biology, University of Kentucky, Lexington, KY USA; 4 Case Comprehensive Cancer Center, Case Western Reserve University, Cleveland, OH USA; 5 Division of Hematology and Oncology, Department of Medicine, Northwestern University Feinberg School of Medicine, Chicago, IL USA; 6 Division of Oncology, Department of Medicine, Washington University in St. Louis, St. Louis, Missouri USA; 7 Department of Electrical and Computer Engineering, TEES-AgriLife Center for Bioinformatics and Genomic Systems Engineering, Texas A&M University, College Station, TX USA; 8 Lurie Comprehensive Cancer Center, Northwestern University Feinberg School of Medicine, Chicago, IL USA; 9 Department of Pathology, Northwestern University Feinberg School of Medicine, Chicago, IL USA

**Keywords:** Cancer stem cells, Metastasis

## Abstract

Circulating tumor cell (CTC) clusters mediate metastasis at a higher efficiency and are associated with lower overall survival in breast cancer compared to single cells. Combining single-cell RNA sequencing and protein analyses, here we report the profiles of primary tumor cells and lung metastases of triple-negative breast cancer (TNBC). ICAM1 expression increases by 200-fold in the lung metastases of three TNBC patient-derived xenografts (PDXs). Depletion of ICAM1 abrogates lung colonization of TNBC cells by inhibiting homotypic tumor cell-tumor cell cluster formation. Machine learning-based algorithms and mutagenesis analyses identify ICAM1 regions responsible for homophilic ICAM1-ICAM1 interactions, thereby directing homotypic tumor cell clustering, as well as heterotypic tumor-endothelial adhesion for trans-endothelial migration. Moreover, ICAM1 promotes metastasis by activating cellular pathways related to cell cycle and stemness. Finally, blocking ICAM1 interactions significantly inhibits CTC cluster formation, tumor cell transendothelial migration, and lung metastasis. Therefore, ICAM1 can serve as a novel therapeutic target for metastasis initiation of TNBC.

## Abstract (web summary)

Circulating tumor cell (CTC) clusters are more efficient at mediating metastasis as compared to single cells and are associated with poor prognosis in breast cancer. Here, the authors show that ICAM1 is enriched in CTC clusters and its loss suppresses cell-cell interaction and CTC cluster formation, and propose ICAM1 as a therapeutic target for treating breast cancer metastasis.

## Introduction

Breast cancer is the most common cancer and the second leading cause of cancer-related deaths among women in the United States^1^. Metastasis accounts for 90% of solid tumor-related mortality and is primarily mediated by hematogenous and lymphatic spread of circulating tumor cells (CTCs) that seed distant organs of the body for secondary tumor growth^2–4^. Compared to single cells, clustered CTCs mediate metastasis at 20–100 times higher efficiency and are associated with lower overall survival of patients with breast cancer^2,5–7^. We previously reported a new mechanism of CTC cluster formation through cellular aggregation instead of cohesive shedding, and demonstrated that the breast tumor-initiating cell marker CD44 directs CTC cluster aggregation, which further enhances stemness of CTC clusters to mediate polyclonal metastasis^8^. However, the molecular mechanisms underlying CTC cluster formation and the cellular heterogeneity of polyclonal metastasis have yet to be fully elucidated.

Triple negative breast cancer (TNBC) represents the clinical subtype of breast cancer that is negative for immunohistochemistry (IHC)-based assessment of estrogen receptor (ER), progesterone receptor (PR), and human epidermal growth factor receptor 2 (HER2) amplification. This clinical subtype has considerable overlap with basal-like breast cancer. TNBC, which represents 10–15% of newly diagnosed breast cancer cases, is considered the most aggressive subtype of breast cancer and is characterized by early recurrence, high incidence of visceral metastasis (to the lungs and liver), and short survival, partially due to a lack of effective targeted therapies^9–14^. The clinical outcomes of TNBC are also partially attributable to subpopulations of tumor-initiating cells (TICs), whose stem cell-like plasticity increases therapeutic resistance, heterogeneous tumor recurrence, and metastasis^15–19^. We set out to identify heterogeneous metastasis-initiating cells using next-generation single-cell RNA sequencing technologies to compare individual cells from paired primary tumors and their spontaneous metastases in patient-derived xenografts (PDXs).

We previously established TNBC PDX models that develop spontaneous micro-metastases to the lungs along with detectable CTC clusters^8,16,20^. Based on single-cell RNA sequencing profiles of primary tumor cells and lung metastases of the PDXs, we identified a subpopulation of lung metastatic cells with ~20-fold higher expression of intercellular adhesion molecule 1 (ICAM1, CD54), compared to that of primary tumor cells. ICAM1 is a cell surface glycoprotein typically expressed on endothelial cells and certain leukocytes^21,22^. Endothelial ICAM1 promotes leukocyte adhesion to endothelium through ICAM1-LFA1 (lymphocyte function-associated antigen 1) and ICAM1-Mac1 (macrophage-1 antigen) intercellular interactions, thereby promoting leukocyte transendothelial migration (TEM)^23–26^. However, the roles of ICAM1 in the development of CTCs, tumor-cluster formation, and metastasis initiation have not been well studied.

Here we report that ICAM1 is a key initiator of metastasis through homophilic ICAM1-ICAM1 interactions that not only promote homotypic CTC cluster formation but also drive tumor-endothelial heterotypic cell adhesion and subsequent TEM. In addition, ICAM1 signaling sustains the levels of cyclin-dependent kinase 6 (CDK6) and other pathway components related to the cell cycle, stemness, and survival. Finally, blocking ICAM1 with anti-ICAM1 neutralizing antibody significantly inhibited tumor cell cluster formation, TEM, and lung colonization. Therefore, we propose that ICAM1 can serve as a novel therapeutic target for metastasis-initiating cells in TNBC.

## Results

### Single-cell RNA sequencing and functional analyses identified ICAM1 as a metastasis initiator

**Fig. 1:**
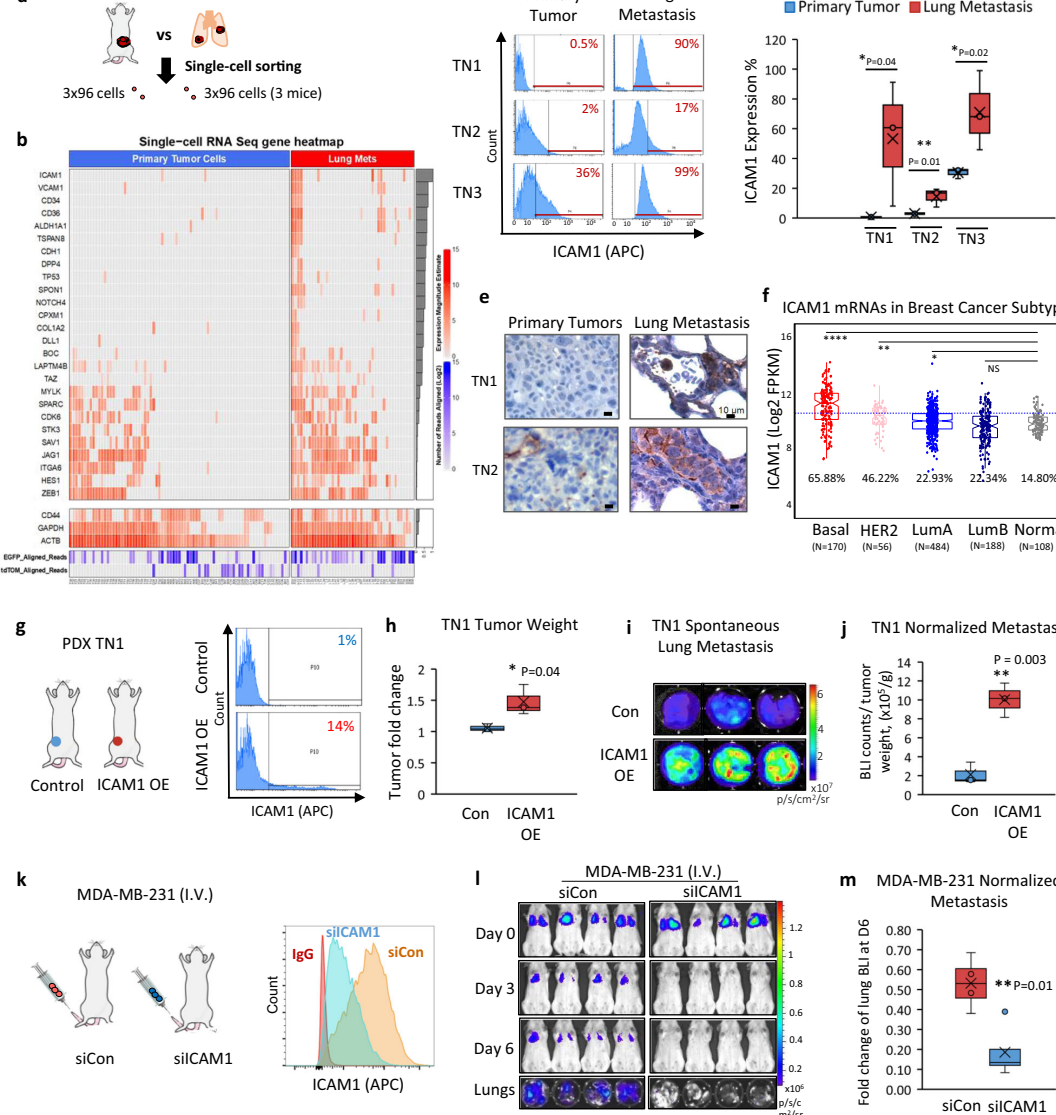
Single-cell RNA sequencing profiles comparing breast cancer cells from primary and metastatic tumor sites. **a** A schematic showing the single-cell RNA sequencing of the sorted cells from L2T- or L2G-labeled TNBC PDX (mice 1 and 2) or MDA-MB-231 tumor (mouse 3)-bearing mice, both primary breast tumors and lung metastases (early micrometastases). **b** Heatmap denoting expression magnitude estimates in log scale (red color) for ICAM1 and co-expressed stemness signature genes in primary tumor cells and lung metastases (*N* = 3 mice, 2 with TN PDXs and 1 with MDA-MB-231 tumor). Genes are sorted according to their correlation with ICAM1 across all cells, as denoted by the gray bars on the right (top highest to bottom lowest). The bottom list of CD44, GAPDH, ACTB, eGFP, and tdTomato serve as control genes without significant changes between primary tumor cells and lung metastases. **c** Representative ICAM1 expression in L2T^+^ or L2G^+^ primary tumor cells and lung metastases determined by flow cytometry from different breast cancer PDX models (TN1, TN2, and TN3). Flow profile gates are shown in Supplementary Fig. 1d. **d** Quantitative data of the differential ICAM1 expression in PDX primary tumors versus lung metastasis from **c**. *n* = 3 biological replicates (mice) for each model. One-sided *t* test *P = 0.04; **P = 0.01; *P = 0.02. The boxes range from the first to third quartile with x in a box indicating mean value and whisker lines extending to outliers (minimum and maximum). **e** Representative IHC staining images of ICAM1 expression (brown color) in primary tumors and lung metastases from PDX TN1 and TN2 models validating **c** and **d**. **f** Distribution of ICAM1 expression across PAM50 subtypes in the TCGA BRCA cohort (*N* = 1037). Basal-like and HER2-enriched subtypes are the top two exhibiting significantly higher ICAM1 expression as compared to normal breast tissue (percentage of cases above the blue line value are shown for each subtype). Statistical significance was assessed using a two-sided Student’s *t* test. *P < 0.05, **P < 0.01, and ****P ≤ 0.0001. **g** Schematic and flow histogram analyses of the orthotopically implanted TN1-PDX tumors with or without ICAM1 overexpression (OE) at the 4th mammary fat pads. **h**. PDX TN1 tumor weights 2 months after orthotopic injections of TN1 cells with ICAM1 OE and control vector (Con) (2.5e5 cells into one mammary fat pad/mouse). *n* = 3 mice per cell group. Two-sided *t* test *P = 0.04. The boxes range from the first to third quartile with x in a box indicating mean value and whisker lines extending to outliers (minimum and maximum). **i**, **j** Representative lung images and normalized BLI signals (total flux) of spontaneous lung metastases in orthotopically implanted ICAM1 OE and control TN1 tumor-bearing mice as in **g**, **h**. *n* = 3 mice per group. Two-sided *t* test **P = 0.003. The boxes range from the first to third quartile with x in a box indicating mean value and whisker lines extending to outliers (minimum and maximum). **k** Schematic and flow histogram analyses of tail vein injected MDA-MB-231 tumor cells transfectd with siRNA control (siCon) and siICAM1. **l**, **m**. Representative images and quantitative data of BLI signal of mice injected with siCon and siICAM1-transfected MDA-MB-231 cells via tail vein (*n* = 4 mice per cell group. Two-sided *t* test **P = 0.01 for time point comparisons. *N* = 6 independent experiments). The boxes range from the first to third quartile with x in a box indicating mean value and whisker lines extending to outliers (minimum and maximum).

We previously developed dual-optical lentiviral reporter vectors expressing luciferase 2-eGFP (L2G) or luciferase 2-tdTomato (L2T) to transduce tumor cells in PDXs^8,16^, which enable the flow analysis, sorting, and fluorescence and bioluminescence imaging (BLI) of labeled tumor cells and metastases. In order to better understand the cellular heterogeneity and identify molecular drivers of metastasis in TNBC, we performed single-cell RNA sequencing to compare individual primary tumor cells with lung metastatic cells sorted from L2G- or L2T-labeled tumor models (Fig. 1a). From three mice bearing TNBC PDXs or MDA-MB-231 tumors, we sorted ~600 single tumor cells (L2G^+^ or L2T^+^) from primary tumors and lung metastases directly into single wells of plates with lysis buffer. Following cDNA library generation, multiple sets of single-cell libraries from paired primary tumors and lung metastases were barcoded for pooled sequencing. The single-cell transcriptomes with high levels of human genome mapping and L2G/L2T tumor cell reporter-gene expression were prioritized for comparison analyses. Two independent analytic strategies were employed to quantify differential mRNA expression between primary tumor cells and lung metastases of each mouse, including a Bayesian approach and a K-Nearest Neighbor (KNN) algorithm^27^. We discovered a signature of 14 overlapping mRNAs exhibiting 2^5^- to 2^10^-fold up-regulation in the lung metastatic cells of two PDXs, including known metastasis-initiating or -promoting genes, such as *CD36*^28^ and *CXCL2*^29^, as well as a list of other genes with less characterized functions in metastasis, such as *ICAM1*, *SELL*, *LGALS9B*, *MGP*, and others (Supplementary Fig. 1a–b and Supplementary Table 1).

Among the newly identified genes specifically up-regulated in the lung metastases of PDXs, *ICAM1* stood out with a 60- to 200-fold increase in the lung metastases (Supplementary Fig. 1a–c). This gene encodes a cell surface protein, intercellular adhesion molecule 1 (ICAM1), also known as CD54, which is involved in vascular adhesion^30,31^. Strikingly, we found that *ICAM1* expression marked a subset cells of lung metastases in the PDXs with concurrent gene expression patterns related to metastasis, tumor initiation, and stem cell functions, such as *VCAM1*, *CD34*, *CD36*, *ALDH1A1*, *TSPAN8*, and *NOTCH4* (Fig. 1b), suggesting *ICAM1* may contribute to metastasis initiation of TNBC.

Using flow cytometry analyses and IHC staining, we first confirmed the highly enriched ICAM1 expression (17–99%) in the lung metastases of three TNBC PDX models (TN1, TN2, and TN3) compared to that of primary tumor cells (0.5–36%, *P* < 0.05 or 0.01) (Fig. 1c–e and Supplementary Fig. 1d). ICAM1 mRNA levels were also found to be more significantly up-regulated in basal-like breast cancers than any other intrinsic subtypes^32^ as compared to normal breast tissues in the TCGA breast cancer cohort (*N* = 1037) (Fig. 1f). Consistent with this, ICAM1 protein levels were also detected at much higher levels in human TNBC cell lines, such as MDA-MB-231, BT-549, FC-IBC-02^33^, and EMF-01^33^, as compared with MCF-7 luminal breast cancer cells via flow cytometry or immunoblotting (Supplementary Fig. 1e–f), as well as in murine TNBC cell lines E0771 and 4T1 (Supplementary Fig. 1g).

To determine the functional importance of ICAM1 in metastasis, we employed two approaches to modulate ICAM1 overexpression (OE) in ICAM1^low^ TNBC PDXs and knockdown in ICAM1^high^ MDA-MB-231 cells. Lentiviral cDNA mediated up-regulation of ICAM1 from 1% to 14% of cells in TN1 PDX models dramatically promoted spontaneous metastasis to the lungs, even upon normalization to the slightly increased tumor weight (Fig. 1g–j). In addition, small interfering RNA (siRNA)-induced ICAM1 knockdown eliminated the lung colonization of MDA-MB-231 cells following tail vein injection into immunodeficient NSG mice (Fig. 1k–m and Supplementary Fig. 2a). To further evaluate the role of ICAM1 in tumorigenesis with a series of cell dilutions, we sorted the ICAM1^+^ (OE) and ICAM1^−^ tumor cells from these transduced PDXs (originally ICAM1^−/low^) (Supplementary Fig. 2b–c). Following orthotopic implantation of 10 cells into mouse mammary fat pads, ICAM1 OE significantly increased the tumorigenicity of TN1 PDX, elevating the TIC frequency from one in 133 ICAM1^−^ cells to about one in 15 ICAM1^+^ cells (Supplementary Fig. 2d–h); however, the tumor initiation or growth from 100 to 1000 cell implants had relatively smaller differences between the two groups (Supplementary Fig. 2d–k). These data demonstrate that ICAM1 promotes both tumorigenesis and metastasis.

### ICAM1 drives CTC cluster formation via tumor cell aggregation

Based on our previous work and other reports, CTC clusters are significantly associated with an unfavorable prognosis for progression-free survival or overall survival, contributing to 20–100 times more efficient metastasis and enhanced stemness and cell-cycle progression relative to single CTCs^2,8,34,35^. We therefore investigated the role of ICAM1 in CTC cluster formation, especially from cellular aggregation^8^, and its downstream signaling pathways.

**Fig. 2:**
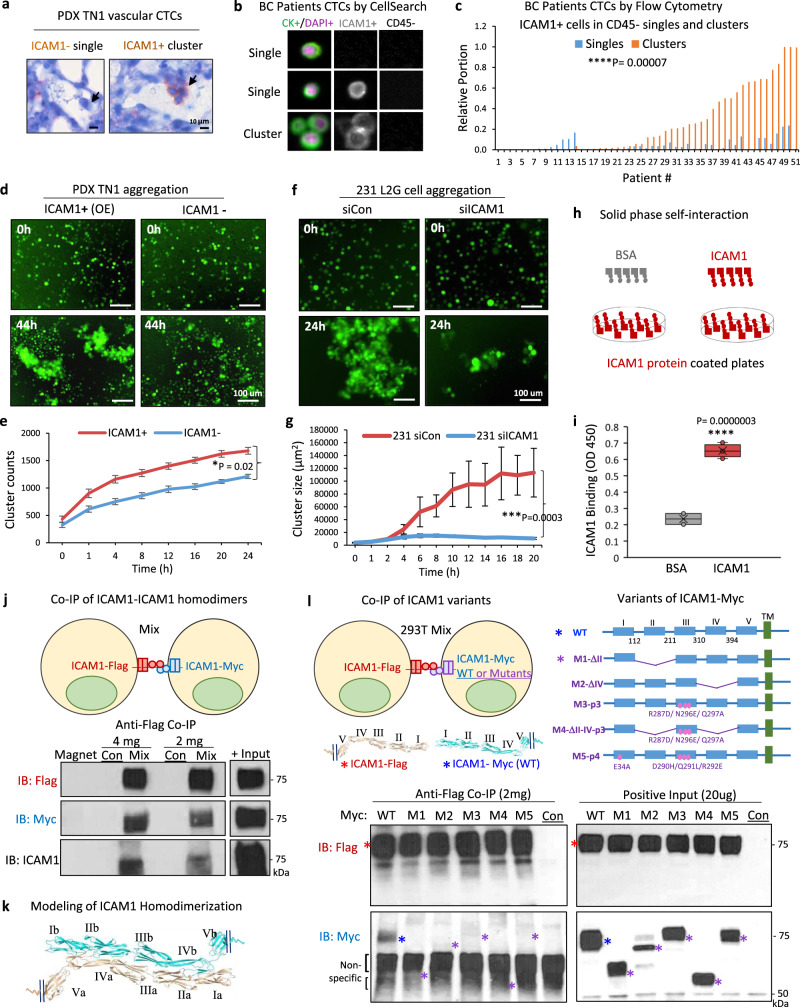
ICAM1 mediates tumor cell clustering through homophilic interaction. **a** Representative IHC staining images of ICAM1^−^ single CTC and ICAM1^+^ CTC cluster (5-cell) (brown) in the lung vasculature of the PDX TN1-bearing mouse. Minimal 3 sets of independent images have shown similar patterns. **b** Representative images of CellSearch-analyzed CTCs in breast cancer (BC) patients: CD45^−^, cytokeratin (CK)^+^ (green), DAPI^+^ (purple), ICAM1^−^ or ICAM1^+^ (gray) (two single cells and a three-cell cluster). **c** Bar graph of the proportion of flow analyzed CD45^−^ICAM1^+^ single (blue) and clustered (orange) CTCs in each of 51 stage III–IV BC patients (*N* = 51 patients. Two-sided *t* test ****P = 0.00007). **d**, **e** Representative images (**d**) and quantitative cluster counts (**e**) of ICAM1^+^ and ICAM1^−^ cells sorted from PDX TN1 OE models showing different cluster formation efficiencies ex vivo (*n* = 10 biological replicates. Data are presented as mean values ± SEM. Error bars represent SE values. Two-sided *t* test *P <  0.02. *N* = 2 independent experiments). **f**, **g** Representative images (**f**) and quantified cluster sizes (**g**) of MDA-MB-231 cells transfected with siRNA control (Con) and siICAM1, and resuspended in poly-HEMA treated plates for cluster formation (*n* = 10 biological replicates. Data are presented as mean values ± SD. Error bars represent SD values. Two-sided *t* test ***P = 0.0003. *N* = 4 independent experiments). **h**, **i** Diagram of solid phase self-interaction assay (**h**) and quantified binding (**i**) of biotin-conjugated ICAM1 and BSA at 1 μg to the solid phase coated with ICAM1 (1 µg), measured as OD_450_ units (two-sided *t* test ****P = 0.0000003. *N* = 2 independent experiments). The boxes range from the first to third quartile with x in a box indicating mean value and whisker lines extending to outliers (minimum and maximum). **j** Co-IP detection of ICAM1-Flag and ICAM-Myc intercellular homodimers. Upper panel, diagram of two HEK-293T cells transfected with C-terminal Flag-tagged and Myc-tagged ICAM1, respectively. Lower panel, immunoblots for Flag, Myc, and ICAM1, respectively, following co-IP with anti-Flag antibody. *N* = 3 independent experiments. **k** ICAM1 extracellular homodimer structure model with interacting domains between II-IV, III-III, and IV-II of two molecules extended from opposite directions. **l** Co-IP detection of ICAM1-Flag with ICAM-Myc variants transfected in two sets of HEK293T cells separately. Upper panel, diagram of two HEK-293T cells transfected with ICAM1-Flag (WT) and ICAM1-Myc variants (1 of 5 different variants). Lower panel, immunoblots for Flag and Myc, following co-IP with anti-Flag showing lost interactions with mutant ICAM1. Colored asterisks indicate the various WT and variant constructs (*N* = 2 independent experiments).

We utilized three methods to detect CTCs and determine if ICAM1 is clinically relevant to CTC clusters and metastasis in breast cancer. First, we detected ICAM1^+^ CTC clusters as well as ICAM1^low^ single CTCs in situ within the vasculature using IHC staining of lung sections of TNBC PDXs (Fig. 2a and Supplementary Fig. 3a). Second, employing the EpCAM-based CellSearch platform^36^ for the blood analysis of nine patients with breast cancer, we detected ICAM1^+^ CTCs in 10–60% of the CTCs (CD45^−^/cytokeratin^+^/DAPI^+^), with a significantly higher proportion of ICAM1^+^ tumor cells in CTC clusters than in single CTCs (Fig. 2b and Supplementary Fig. 3b–c). In order to analyze all the putative CD45^−^ CTCs independent of EpCAM expression, we developed a third complementary approach, flow cytometry-based CTC analysis of patient blood samples following red blood cell lysis and white blood cell isolation. Based on the flow profiles of 30 blood samples collected from advanced breast cancer patients at Northwestern University in a prospective study, we found that ICAM1 expression was significantly enriched in CTC clusters compared to that of single CTCs (Fig. 2c and Supplementary Fig. 3d). In addition, immunofluorescence staining of MDA-MB-231 tumor cells showed higher expression of ICAM1 in the aggregated tumor cells compared to that of single tumor cells (24 h aggregation) (Supplementary Fig. 3e, white arrows pointing to single tumor cells). By immunoblotting, we also found that ICAM1 expression gradually increased upon clustering over time (Supplementary Fig. 3f).

To determine if ICAM1 drives tumor-cluster formation or cellular aggregation, we first sorted ICAM1^+^ and ICAM1^−^ tumor cells from L2G-labeled TN3 PDX, which had partial ICAM1 expression for clustering assays in vitro as previously described^8^. We also overexpressed ICAM1 into L2G-labeled ICAM1-negative TN1 PDXs for sorting of ICAM1-overexpressing (ICAM1^+^ OE) and ICAM1^−^ tumor cells for clustering assay. We observed that ICAM1^+^ tumor cells sorted from TN1 (ICAM1-OE) and TN3 PDXs formed bigger clusters than their counterpart ICAM1^−^ tumor cells over time as measured by IncuCyte imaging (Fig. 2d, e and Supplementary Fig. 4a). Furthermore, ICAM1 knockdown in MDA-MB-231 cells as well as TN3 PDXs dramatically inhibited the tumor cell aggregation (Fig. 2f, g and Supplementary Fig. 4b).

Since we previously demonstrated that CD44 mediates tumor cell aggregation, we next examined whether there is a regulatory correlation between CD44 and ICAM1 in tumor clustering. Analyzed by immunoblotting and flow cytometry, siRNA-mediated knockdown of ICAM1 did not affect the expression of CD44 and vice versa, CD44 knockdown did not alter ICAM1 levels (Supplementary Fig. S4c–d). Notably, ICAM1 knockdown resulted in more profound effects than CD44 knockdown in inhibiting the cluster formation of these cells (Supplementary Fig. 4e). When compared to ICAM1^+^ and CD44^+^ single-positive TNBC cells, ICAM1^+^CD44^+^ double positive cells showed a better clustering efficiency with more than 5-fold bigger clusters (Supplementary Fig. 4f), indicating a potential cooperation between ICAM1- and CD44-mediated two independent pathways in cell aggregation.

### ICAM1 mediates tumor cell aggregation through intercellular homophilic interactions

To understand how ICAM1 mediates tumor cell aggregation, we first investigated if tumor cells express the known ligands of ICAM1, LFA1 (CD11a/CD18), and MAC1 (CD11b/CD18), both of which belong to the beta 2 integrin family (ITGB2, CD18) and are normally expressed on leukocytes^30^. However, breast cancer cells such as MDA-MB-231 had undetectable levels of integrin β2, a common subunit of LFA1 and MAC1 (Supplementary Fig. 5a). Instead, we observed a small amount of ICAM1 at higher molecular weights as potential dimers (~140 kD*) or tetramers (~280 kD^#^) in addition to the glycosylated monomers at ~75 kD in MDA-MB-231 aggregates in the presence of crosslinking reagent DSS (Supplementary Fig. 5b). Since we have previously shown that CD44 mediates cell aggregation via intercellular interaction [8], we utilized multiple experimental approaches to test whether ICAM1 also forms homophilic dimers from neighboring cells, thereby resulting in tumor cell aggregation.

We first performed a solid-phase, self-interaction assay with purified recombinant protein of the His-tagged ICAM1 extracellular domain (ExD) (Fig. 2h). After ICAM1 ExD coated the test plates (solid phase), it showed significant binding to biotin-labeled ICAM1 ExD versus the BSA control (Fig. 2h, i), suggesting possible homophilic interactions between two ICAM1 ExDs. Second, we determined if ICAM1 mediates intercellular self-interactions from two neighboring cells. ICAM1 with two different C-terminal tags, ICAM1-Flag and ICAM1-Myc, was overexpressed into two separate sets of ICAM1^−^ cells. Upon collection, ICAM1-Flag-expressing cells were then mixed and aggregated with ICAM1-Myc-expressing cells (Fig. 2j, top). Aggregated cells were harvested and lysed for co-immunoprecipitation (co-IP) with an anti-Flag antibody. Upon immunoblotting of both proteins in the same pulldown lysate, we revealed the intercellular, homophilic interactions between ICAM1-Flag and ICAM1-Myc proteins (Fig. 2j, bottom). These data demonstrate that ICAM1 directs tumor cell aggregation through its intercellular ICAM1-ICAM1 homophilic interactions, but do not rule out that homophilic interactions in cis^37^ can also occur to possibly make tetramers.

To elucidate the molecular regions required for ICAM homophilic interactions, we analyzed the potential ICAM1 self-binding sites. Based on the predictions of machine learning-assisted structure modeling algorithms^38,39^, ICAM1 ExD contains five domains (I–V), with the C-terminal domain V linked to the transmembrane segment, and the homodimer interface regions spanning certain regions of domains II to IV (Supplementary Fig. 5c). Multiple self-interacting dimer models were predicted across two ICAM1 molecules that extend from two neighboring cells, showing intercellular II-IV, III-III, and IV-II interactions as well as intercellular III-IV and IV-III interactions (Fig. 2k and Supplementary Fig. 5d), with the possibility of intermolecular β-sheet formation being involved in some of the intercellular dimer interfaces.

To verify the molecular basis of ICAM1 dimerization, we generated a series of ICAM1 variants using the *ICAM1-Myc* vector, named M1-M5, including M1 with truncated domain II (Δ112-211), M2 with truncated domain IV (Δ310-394), M3 with predicted homodimer-disruptive mutations R287D/N296E/Q297A in domain III, M4 with combined mutations (M1-M2-M3), and M5 with mutated binding sites for LFA1 and MAC1, E34A in domain I and D290H/Q291L/R292E in domain III, respectively (Fig. 2l top panels). We found that all of the ICAM1-Myc mutants lost their homophilic interactions with ICAM1-Flag in the co-IP assays (Fig. 2l bottom panels), suggesting that multiple regions of ICAM1 are involved in the self-dimerization.

### ICAM1-regulated pathways and targets

**Fig. 3:**
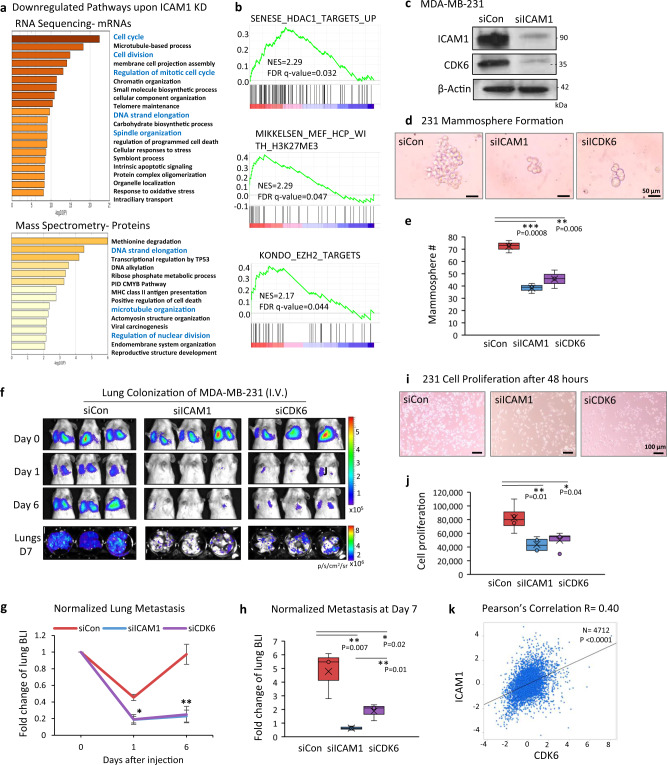
Downstream targets of ICAM1 in regulating metastasis. **a** Down-regulated pathways upon *ICAM1* knockdown in MDA-MB-231 cells, analyzed by RNA sequencing (top) and mass spectrometry analysis (bottom). **b** GSEA of the gene sets for histone deacetylase targets, H3K27ME3, and EZH2 targets enriched among the up-regulated genes in MDA-MB-231 *ICAM1* knockdown cells in comparison to siRNA control, identified by RNA sequencing. **c** Immunoblots of ICAM1 and CDK6 in MDA-MB-231 cells transfected with control siRNAs (siCon) and siICAM1 for gene knockdown. *N* = 3 independent experiments. **d**, **e** Representative images (**d**) and mammosphere quantitation (**e**) of MDA-MB-231 cells transfected with siCon, siICAM1, and siCDK6 (*n* = 3 biological replicates, two-sided *t* test **P = 0.006; ***P = 0.0008. *N* = 3 independent experiments. The boxes range from the first to third quartile with x in a box indicating mean value and whisker lines extending to outliers (minimum and maximum). **f**–**h** Representative images (**f**) and quantitative data (**g**, **h**) of BLI signals of mice injectd with siCon, siICAM1, and siCDK6-transfected MDA-MB-231 cells via tail vein (*n* = 3 mice per cell group. **g** Data are presented as mean values ± SD, two-sided *t* test *P < 0.05; **P ≤ 0.01. *N* = 4 independent experiments. **h** The boxes range from the first to third quartile with x in a box indicating mean value and whisker lines extending to outliers (minimum and maximum). **i**, **j** Representative images (**i**) and counts (**j**) of proliferative MDA-MB-231 (50k cells/well) transfected with siCon, siICAM1, and siCDK6. Cell numbers were measured via hemocytometer counting 48 h after seeding (*n* = 4 biological replicates. two-sided *t* test *P = 0.04; **P = 0.01. *N* = 3 independent experiments. The boxes range from the first to third quartile with x in a box indicating mean value and whisker lines extending to outliers (minimum and maximum). **k** Pearson’s pairwise correlation plot of ICAM1 versus CDK6 expression in breast cancer patients (*n* = 4712), by BC Gene-Expression Miner v4.4. ICAM1 vs. CDK6 *R* = 0.40. ****P < 0.0001.

Next, we examined the role of ICAM1-induced molecular alterations of breast cancer cells in metastasis. We employed RNA sequencing and mass spectrometry for transcriptome and proteome analyses of MDA-MB-231 cells upon siICAM1-mediated knockdown (Fig. 3a; Supplementary Fig. 6a–b; and Supplementary Tables 2 and 3). Multiple pathways were down-regulated in siICAM1-transfected cells, including stemness, the cell cycle, hypoxia and HIF-1 targets, microtubule-based processes, plasma membrane cell projection assembly, telomere maintenance, and cell survival (Fig. 3a and Supplementary Fig. 6b–d). Other pathways involved in histone modification, autophagy, and mammary differentiation were up-regulated in siICAM1-transfected cells (Fig. 3b and Supplementary Fig. 6e–h). These data suggest that ICAM1 signaling enhances cancer stemness and cell-cycle progression, and suppresses epithelial differentiation. Using immunoblotting analyses of cell lysates, we confirmed that ICAM1 knockdown not only reduced protein levels of the top targets related to stemness and cell-cycle regulation, such as CDK6, OCT3/4, NOTCH1, MCM3, ZEB1, Sec23a, and HIF1A; but also up-regulated proteins related to epithelial differentiation and stress signaling, such as KRT19, PAI1, and HMGA2 (Fig. 3c and Supplementary Fig. 6c, f).

We chose a few candidates with the highest expression changes detectable by RNA-seq for siRNA-mediated knockdown in metastasis initiation-related functional studies. While knockdown of CDK6, ZEB1, and Sec23a expression compromised mammosphere formation, tumor cell migration, and/or clustering of MDA-MB-231 cells (Fig. 3d, e and Supplementary Fig. 7a–f), only siCDK6 partially mimicked siICAM1 in inhibiting lung colonization of MDA-MB-231 cancer cells within 7 days after tail vein injection (Fig. 3f–h and Supplementary Fig. 7g–i). This finding suggests that CDK6 is one of the most essential targets required for ICAM1-mediated metastasis. We also found that CDK6 knockdown mimicked ICAM1 knockdown in reducing cell-cycle progression or proliferation of MDA-MB-231 cells (Fig. 3i, j and Supplementary Fig. 7j). Meta-analysis of various breast cancer gene expression datasets using bc-GenExMiner and the TCGA database further demonstrated a significant positive correlation between *ICAM1* with *CDK6* mRNA levels (Fig. 3k and Supplementary Fig. 7k).

We also compared the effects of knocking down ICAM 1 and CDK6 on early metastatic seeding and long-term growth of lung metastasis. At 6–8 h after tail vein injection, siICAM1-transfected cells showed decreased seeding to the lungs, whereas si-CDK6 transfected cells had similar efficiency of seeding as the control cells (Supplementary Fig. 8a, b), suggesting ICAM1-mediated metastatic seeding is cell-cycle independent. Nevertheless, the long-term growth of metastatic cells was dependent on the downstream target of ICAM1, as siCDK6 dramatically slowed the lung colonization within 2 weeks (Supplementary Fig. 8c–e). To demonstrate the rigor and reproducibility, we had also tested two other individual siRNAs for ICAM1 knockdown. Both siRNA-A and siRNA-B had similar effects as the smart pool siRNAs on inhibiting ICAM1 expression and blocking lung colonization (Supplementary Fig. S9a–d). Furthermore, both individual siRNA-A and –B of ICAM1 had interfered the cluster formation, mammosphere formation, and cell proliferation as the smart-pool siRNAs (Supplementary Fig. S9e–j).

### ICAM1 mediates transendothelial migration of breast cancer cells

**Fig. 4:**
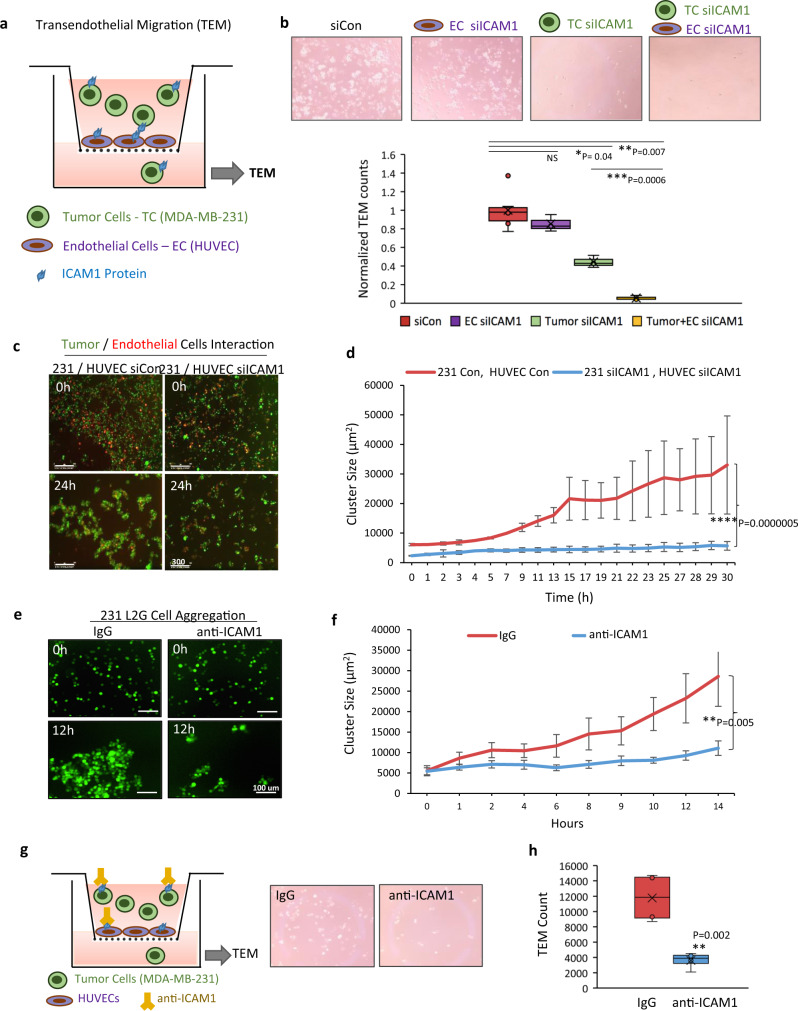
ICAM1 mediates transendothelial migration of breast cancer cells. **a** Diagram of TEM assay with HUVEC cell-coated transwell inserts and tumor cells seeded on the top. Tumor cells transmigrated to the bottom were measured 24 h after seeding. (MDA-MB-231 tumor cells; green, HUVEC; purple, and ICAM1 protein; blue). **b** Representative images (top) and quantitative analyses (bottom) of TNBC cells transmigrated to the bottom chamber, including four conditions (from left to right): (1) siRNA control tumor cells and HUVECs; (2) *ICAM1* knockdown in endothelial cells (EC); (3) *ICAM1* knockdown in tumor cells; (4) *ICAM1* knockdown in both tumor cells and HUVECs (*n* = 3 biological replicates. Two-sided *t* test *P = 0.04; **P = 0.007; ***P = 0.0006. NS not significant. The boxes range from the first to third quartile with x in a box indicating mean value and whisker lines extending to outliers (minimum and maximum). **c**, **d** Time-lapse co-culture images at 0 and 24 h of incubation with TNBC cells (green) and endothelial cells (red) (**c**) and quantitative analyses of aggregates (**d**) following *ICAM1* knockdown in both cell types (*n* = 8 biological replicates. Data are presented as mean values ± SD. Error bars represent SD values. Two-sided *t* test ****P = 0.0000005). **e**, **f** Representative images (**e**) and quantitative analyses (**f**) of TNBC cell cluster formation in the presence of IgG control or anti-ICAM1 neutralizing antibody (*n* = 10 biological replicates, Data are presented as mean values ± SEM. Error bars represent SE values. Two-sided *t* test **P = 0.005). **g**, **h** Diagram and representative images of TEM assay (**g**) and quantitative analysis (**h**) of breast tumor cells transmigrated to the bottom chamber in the presence of IgG control or anti-ICAM1 neutralizing antibody (*n* = 3 biological replicates. Two-sided *t* test **P = 0.002). (Diagram: MDA-MB-231 tumor cells; green, HUVEC; purple, ICAM1 protein; blue, and anti-ICAM1 antibody; yellow). The boxes range from the first to third quartile with x in a box indicating mean value and whisker lines extending to outliers (minimum and maximum).

Since prior work has demonstrated that ICAM1 is expressed in endothelial cells and facilitates leukocyte endothelial transmigration, we examined if ICAM1 in tumor cells interacts with ICAM1 and other possible ligands in endothelial cells to promote trans-endothelial migration (TEM). ICAM1 is also known to bind integrin ligands LFA1 and MAC1, which are mostly expressed in lymphocytes and macrophages^23–26^. We first measured the ICAM1 and its integrin ligands by flow cytometry in endothelial cells and found an absence of the integrin ligand CD18 (part of LFA1 and MAC1) (Supplementary Fig. 10a). Using the transwell insert pre-coated with confluent human umbilical vein endothelial cells (HUVECs), we added breast tumor cells on top of the endothelial cells in the insert under four different conditions: (i) control siRNA in endothelial cells, (ii) ICAM1 knockdown in endothelial cells alone, (iii) in tumor cells alone, or (iv) in both tumor cells and endothelial cells (Fig. 4a). After 24 h, we observed distinct TEM of tumor cells to the bottom chamber in control cells (Fig. 4b). The ICAM1 knockdown in endothelial cells alone slightly inhibited the TEM of tumor cells, whereas the ICAM1 knockdown in tumor cells resulted in a much more significant reduction in the TEM. Moreover, ICAM1 knockdown in both tumor cells and endothelial cells synergized to almost completely block the TEM (Fig. 4b and Supplementary Fig. 10b). In another assay to examine the interactions of breast cancer cells and endothelial cells, we initiated a mixed culture of breast tumor cells and HUVECs (2:1 ratio) in suspension. This also caused heterotypic aggregation between these two cell types, which was inhibited by ICAM1 knockdown in both tumor cells and endothelial cells (Fig. 4c, d).

We next assessed the therapeutic effects of an anti-ICAM1 neutralizing antibody on breast tumor cell aggregation and TEM. Anti-ICAM1 treatment greatly inhibited the cluster formation of TNBC cells in suspension (Fig. 4e, f). The anti-ICAM1 antibody also significantly blocked TEM of L2G-labeled breast tumor cells in a transwell insert pre-coated with confluent HUVECs as described above (Fig. 4g, h). These data suggest that ICAM1 inhibition blocks heterotypic interactions between tumor cells and endothelial cells, in addition to interfering with homotypic tumor cell-tumor cell interactions.

### ICAM1 is a therapeutic target in TNBC metastasis in vivo

**Fig. 5:**
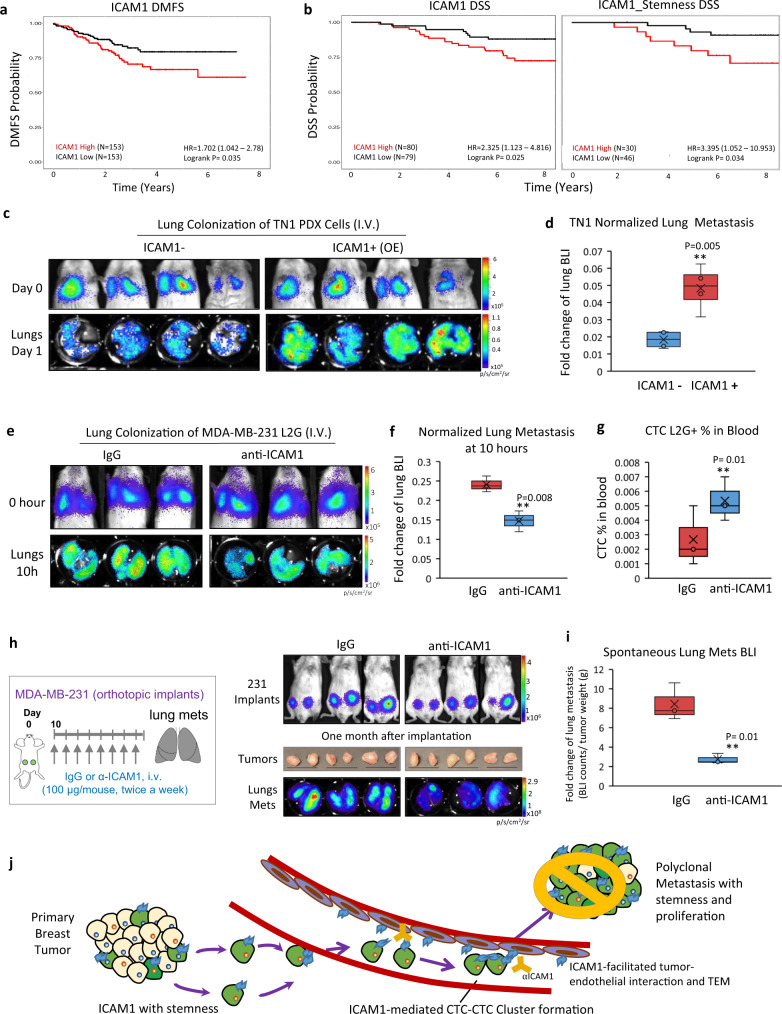
ICAM1 is a therapeutic target of TNBC metastasis. **a** Differences in distant metastasis-free survival (DMFS) of patients with breast tumors exhibiting high versus low ICAM1 expression in the GSE25055 (*N* = 306) cohort. Note that patients with tumors expressing higher than median ICAM1 expression (red) are associated with significantly poorer DMFS (Logrank *P* = 0.035). **b** Differences in disease-specific survival (DSS) of patients with breast tumors exhibiting high versus low ICAM1 expression alone (left panel) or in combination with a 98-gene Stemness Signature Index (right panel) in the GSE1456-GPL96 (*N* = 159) cohort. Note that patients with tumors harboring higher than median ICAM1 expression (left panel, red) exhibit significantly poorer DSS (Logrank *P* = 0.025). Similarly, breast cancers expressing higher than median Stemness Signature Index in addition to higher than median ICAM1 expression (Dual-High, red) exhibit poorer DSS (Logrank *P* = 0.034). **c**, **d** Representative images (**c**) and quantitative data (**d**) of BLI signals in mice on Day 0 (D0, 0 h) and Day 1 (D1, 24 h, dissected lungs) after injection of sorted ICAM1^+^ and ICAM1^−^ TN1 PDX cells via tail vein (*n* = 4 mice per cell group. Two-sided *t* test **P = 0.005). The boxes range from the first to third quartile with x in a box indicating mean value and whisker lines extending to outliers (minimum and maximum). **e**–**g** Representative BLI images of mice at 0 h and dissected lungs at 10 h after tumor cell injections via tail vein (**e**), normalized metastatic seeding to the lungs (**f**), and L2G^+^ CTC analysis (%) in the blood (**g**). The mice were pretreated with IgG or anti-ICAM1 neutralizing antibody (80 µg/mouse) via tail vein, and 3 h later followed by a tail vein injection of MDA-MB-231 cells (1 × 10^5^ cells) and the antibody (100 μg, pre-incubated with cells for 30 min). Lungs were collected 10 h after injection. (*n* = 3 mice per group. Two-sided *t* test **P = 0.008; **P = 0.01. *N* = 2 independent experiments. The boxes range from the first to third quartile with x in a box indicating mean value and whisker lines extending to outliers (minimum and maximum). **h**, **i** Representative BLI images of mice on day 0 post orthotopic implantations of MDA-MB-231 tumor cells, pictures of dissected breast tumors, and BLI of dissected lungs (**h**) and quantitative lung metastasis after normalization by tumor weight of each mouse (**i**). The mice were given long-term treatment with IgG or anti-ICAM1 antibody (100 µg/mouse, twice a week for 4 weeks) (*n* = 3 mice per group. Two-sided *t* test **P = 0.01). The boxes range from the first to third quartile with x in a box indicating mean value and whisker lines extending to outliers (minimum and maximum). **j** Diagram of ICAM1^+^ tumor cells initiating multicellular cluster formation in the circulation, directing TEM, and mediating lung metastasis; partially through sustained expression of CDK6. Blocking the ICAM1 intercellular homophilic interactions between tumor-tumor and tumor-endothelial cells with neutralizing antibodies (gold) will inhibit CTC cluster formation and TEM, and eventually decrease or block metastasis. (ICAM1^+^ tumor cells; green, ICAM1 protein; blue, ICAM1^−^ tumor cells; beige, Endothelial cells; purple, and anti-ICAM1 antibody; yellow).

To determine if ICAM1 is a valid target in TNBC and metastasis, we further examined the clinical relevance of ICAM1 expression to patient outcomes by analyzing two independent breast cancer patient cohorts, GSE25055^40^ and GSE1456^41^. Indeed, high levels of ICAM1 mRNA expression in breast tumors were associated with an unfavorable distant metastasis-free survival (DMFS) (Fig. 5a) as well as disease-specific survival (DSS) (ICAM1 alone and along with the stemness signature genes) (Fig. 5b).

We assessed if ICAM1 promotes metastatic seeding and extravasation of TNBC cells following tail vein inoculation. ICAM1-overexpressing and negative control cells were sorted from TN1 PDX tumors for such analysis, and overexpression of ICAM1 in cells significantly increased the seeding to the lungs when measured 1 day after intravenous injection (Fig. 5c, d). We continued to evaluate the therapeutic effects of an anti-ICAM1 neutralizing antibody on early seeding of TNBC. Mice were pretreated intravenously with the first dosage of anti-ICAM1 neutralizing antibody or IgG (80 ${{{\rm{\mu }}}}$g/mouse) for 3 h on day 0, and then injected via tail vein the MDA-MB-231 cells (1 × 10^5^) pre-mixed with the second dosage of anti-ICAM1 or IgG (100 μg/mouse). When measured at 10 h after tumor cell injection, anti-ICAM1 effectively reduced the extravasation and metastatic seeding of MDA-MB-231 cells to the lungs in NSG mice, coupled with decreased metastatic signals in the lung and increased number of CTCs trapped in the circulation (Fig. 5e–g). The inhibited lung colonization was further confirmed by bioluminescence imaging on day 1 and 2 post injection (Supplementary Fig. 10c–d).

We next evaluated the therapeutic effects of an anti-ICAM1 neutralizing antibody on spontaneous metastasis of TNBC. Once palpable tumors started to form after orthotopic implantation of MDA-MB-231 cells, anti-ICAM1 or IgG was administered twice a week for 4 weeks with both intravenous dosage (80 µg/mouse) via tail vein and subcutaneous dosages into the 4th mammary fat pads near tumor implants (10 µg/tumor, both left and right sides). While anti-ICAM1 has no significant effects on primary tumor growth, it dramatically reduced spontaneous lung metastasis (Fig. 5h, i).

Overall, our findings support that ICAM1 plays a key role in the initiation of metastasis by: (i) mediating tumor cell aggregation through homotypic ICAM1-ICAM1 interactions for CTC cluster formation; (ii) enhancing stemness by upregulating stemness-related genes; (iii) enhancing tumor cell-endothelial cell cross-talk through heterotypic ICAM1-ICAM1 interactions, which promote TEM; and (iv) promoting tumor cell proliferation through sustaining or activating downstream CDK6. Importantly, anti-ICAM1 neutralizing antibody as proof-of-concept inhibits cell aggregation, tumor cell TEM, and metastasis in TNBC (Fig. 5j).

## Discussion

Combining the cutting-edge single-cell RNA sequencing analysis of micro-metastases and molecular function studies, our work elucidates a new ICAM1-mediated pathway independent of CD44 in initiating CTC cluster formation and driving lung metastasis of TNBC. While ICAM1 is known to interact with β2 integrins in leukocytes, these ligands are not detectable in breast cancer cells. Instead, using artificial intelligence-based structural modeling in combination with experimental testing, our studies reveal that ICAM1 is capable of mediating homophilic interactions to drive homotypic CTC-CTC cluster formation as well as heterotypic tumor-endothelial cell adhesion. While the molecular mechanisms underlying ICAM1’s function in tumor cells are not well defined, our study has successfully employed machine learning-based protein structure modeling to predict the ICAM1 dimerization residues for mutagenesis analyses. Deep-learning algorithms with augmented intelligence can also be applied to other bioinformatic analyses and therapeutic development. In breast tumor cells, ICAM1 promotes many pathways and signature genes related to stemness and the cell cycle, including CDK6 as one of the essential targets. Notably, our studies have also identified the gene set enrichment analysis (GSEA) signatures of ICAM1 related to HDAC and HIF1 targets, consistent with published reports on DNA modifications of CTC clusters in breast cancer and metabolic switching of tumor cell clusters with hypoxic signaling in other cancers^34,42^.

Metastasis is mainly mediated by the spread of CTCs, which are frequently observed in the blood of advanced cancer patients^2,3^. While CTC clusters have been recently elucidated to mediate metastasis at a high efficiency and correlate with an unfavorable prognosis in cancer patients^2,7,8^, there are no existing therapeutics specifically targeting CTC clusters. Here we reveal a new mechanism of CTC cluster formation in which ICAM1 serves as a therapeutic target. As proof-of-concept, anti-ICAM1 neutralizing antibody can effectively block tumor cell cluster formation and TEM, thereby inhibiting lung metastasis. From a therapeutic standpoint, considering the multiple intercellular interactions ICAM1 may mediate in tumor initiation and metastasis, we expect that the anti-ICAM1 blockade approach can potentially be added to a combination with other existing therapeutics for TNBC, such as chemotherapeutic agents, PARP inhibitors, anti-vascular reagents, and possibly immunotherapy. Specific domains of ICAM-1 bind to LFA-1 (Ig domain 1) and to Mac1 (Ig domain 3) on leukocytes^43,44^. Our data (Fig. 2l) suggest that multiple regions of ICAM-1 are involved in homophilic clustering of CTC. Identifying the specific regions of ICAM-1 involved in this binding, a goal of future research, may reveal sites on ICAM-1 that can be targeted therapeutically without compromising the immune functions of LFA-1 and Mac1 interactions with ICAM-1. While CD44^+^ breast tumor-initiating cells can contribute to CTC cluster formation and lung metastasis of TNBC^8^, our work suggests that ICAM1 and CD44 independently direct cancer metastasis. Therefore, targeting both pathways would be necessary for future clinical applications.

Inflammatory signals and cytokines such as NF-kB and IL-6 have been known to induce the up-regulation of ICAM1 in endothelial cells and tumor cells^45–47^. Given the high ICAM1 expression in inflammatory breast cancer cell lines FC-IBC-02 and EMF-01 we generated from plural effusion^33^, the blood and lung microenvironment may be not only favorably selecting rare ICAM1^+^ tumor cells with their propensity to cluster, transmigrate, and metastasize, but also capable of providing niche-specific signals to enhance stemness/plasticity in tumor cells with ICAM1 expression for lung metastasis.

While our research focused on elucidating the role of ICAM1 in lung metastasis of TNBC, its functional significance in the lung metastasis of other cancer types needs to be further investigated. ICAM1 has recently been shown to be expressed in several types of tumors^22^ and may be involved in bone metastasis^48^. Any context-dependent functions of ICAM1 in other subtypes and types of cancers, including melanoma and colon cancer cells^25,49,50^, breast cancer^51–53^, gastric cancer^54^, multiple myeloma^55,56^, and esophageal squamous cell carcinoma^57^ require deep mechanistic elucidation and comprehensive human tumor dataset validation.

Overall, our findings on ICAM1’s association with patient outcomes as well as its ability to mediate CTC cluster formation and tumor-endothelial cell adhesion through ICAM1-ICAM1 homophilic interactions provide a strong rationale for therapeutic targeting and prognostic evaluation of tumor-intrinsic and -extrinsic ICAM1 in breast cancer metastasis.

## Methods

### Human specimen analyses

The human specimen collection and blood sample analyses were approved by the Northwestern University Institutional Review Board following NIH guidelines for human subject studies. Written consent was obtained from all patients whose blood samples were analyzed for the study.

### Animal studies and TNBC PDX models

All mice used in this study were housed in specific pathogen-free facilities, with regular diet and regular light/dark cycles, and regular ambient temperature and humidity in the Animal Resources Facilities at Northwestern University. All animal procedures conformed to the NIH Guidelines for the Care and Use of Laboratory Animals and were accepted by the Northwestern University Institutional Animal Care and Use Committees. Female NSG mice at age of 6–8 weeks (Jackson Laboratory) were randomized by age and weight for human PDX inoculations or injections. The exclusion of mice from experiments was based on criteria of illness or disorders unrelated to tumors. Sample sizes were specified based on the results of preliminary experiments.

Human PDX models were established from TNBC patient breast tumor samples or pleural effusion and labeled by dual-reporter genes L2G or L2T to enable tracking of tumor cells growth and metastasis in vivo, as few as 10 cells only^16^. These models were passaged and propagated upon orthotopic implantation of sliced tumor specimen into mouse mammary fat pads in NSG immunodeficient mice. Harvested PDX tumors are minced then dissociated using Collagenase and DNase, and finally filtered with 40-µm nylon strainer to reach single-cell suspension. Dissociated tumor cells might be used for terminal experiments ex vivo without further propagation or maintenance, such as cluster formation analysis on collagen-coated plates. NSG mice were also used for orthotopic implantations of other human TNBC cell lines such as MDA-MB-231 cells and tail vein injections of multiple PDX tumor models^16^ and MDA-MB-231 breast tumor cells expressing L2T or L2G. The IVIS® Spectrum imaging system was used for imaging of primary tumor signals and lung metastasis in all in vivo experiments.

### Cell lines and transfections

MDA-MB-231 and HEK-293 cells were obtained from ATCC and periodically tested to be mycoplasma-negative using Lonza’s MycoAlert Mycoplasma Detection Kit (cat #LT07-218). Phenotypic and transcriptome analyses of these cells corresponded with the published profiles. Early passage of cells (<20 passages) was cultured in DMEM high glucose, supplemented with 10% FBS + 1% penicillin–streptomycin (P/S). PDX primary tumor cells were cultured in HuMEC-ready medium (Life Technologies) plus 5% FBS and 0.5% P/S in collagen type I (BD Biosciences) coated plates.

Breast tumor cells were transfected with Dharmacon siRNAs using Dharmafect (Dharmacon) at 100 nmol/L, and retransfected after 48 h. Cells were collected for experiments 2 days after the second transfection. For overexpression of ICAM1 in HEK-293 cells, the following plasmids were transfected into HEK-293 cells by PolyJet (SignaGen Laboratories): ICAM1 cDNA ORF Clone, Human, C-Flag tag (Sino Biological, Cat# HG10346-CF); and ICAM-1 cDNA ORF Clone, Human, C-Myc tag (Sino Biological, Cat# HG10346-CM). Cells were collected for co-IP and western blotting 48 h after transfection.

### Cell preparation for single-cell RNA sequencing

L2T or L2G-labeled TNBC primary tumor cells and lung metastatic cells were dissociated from three mice, with two bearing PDXs and one bearing MDA-MB-231 tumors. L2T^+^ or L2G^+^ single tumor cells were directly sorted into individual wells (one cell/well) of 96-well plates containing 1X reaction buffer with the RNase Inhibitor (SMART-Seq™ V3 UltraTM Low Input RNA Kit for Sequencing, Clontech) by BD sorter based on tdTomato or GFP fluorescence. The samples were then flash frozen with liquid nitrogen for future use. cDNAs were created using a SMART-Seq™ V3 UltraTM Low Input RNA Kit for Sequencing (Clontech) following the manufacturer’s instructions. The cDNA samples were then validated using an Agilent 2100 Bioanalyzer. The suitable samples were chosen to prepare libraries for sequencing on 96-well plates using a Nextera XT DNA Library Preparation Kit (Illumina). All single-cell libraries were sequenced on the HiSeq 2500 platform to obtain 2–4 million single-end 50-bp reads per sample by the Case Western Reserve University sequencing core.

### Single-cell RNA sequencing analysis

Single-cell RNA sequencing data was obtained for 96 single cells from primary and lung metastases in each of three independent mouse xenograft experiments, including two with PDXs and one with MDA-MB-231 tumors. We prioritized the analyses of those cells with high levels of L2G/L2T expression and human genome mapping. These included 15 cells of lung metastases and 15 cells of the primary tumor from Mouse 1 (PDX); 33 cells of lung metastases and 33 cells of the primary tumor from Mouse 2 (PDX); and 32 cells of the primary tumor, 32 cells of lung metastases, and 32 circulating tumor cells from Mouse 3 (MDA-MB-231). Raw sequencing reads were aligned to GrCH37 (human) and mm9 (mouse) reference genomes, in addition to aligning to eGFP and tdTom sequences using Bowtie2 (56). Cells that did not express either eGFP or tdTom, or exhibited higher levels of alignment to the mouse as compared to the human reference genome, were considered to be mouse-derived stromal cells and were excluded from downstream analyses. This resulted in a total of 13, 32, and 32 primary tumor cells from Mouse 1, Mouse 2, and Mouse 3, respectively. Similarly, the number of lung metastasis cells were 12, 4, and 27 from Mouse 1, Mouse 2, and Mouse 3, respectively. Individual gene definitions were obtained from Ensembl Grch37, followed by the calculation of read-count assessments per cell using featureCounts (version 1.5.0) (57). The SCDE analytics framework (version 1.99.2) (27) was employed to determine differential gene expression between cells from lung metastases as compared to cells from primary xenografts. The SCDE package provides a collection of statistical methods for analyzing single-cell RNA sequencing data. The SCDE method was developed to model the probability of dropout events for each gene within each cell, thus providing a posterior likelihood of true differential expression of genes between cells. In order to increase the sensitivity of the analysis, both the SCDE and KNN models implemented within the SCDE package were used to calculate the differential expression of genes. Genes with <10 reads aligned across all cells from a specific experiment were removed. The error models were fitted independently for each cell group within each mouse experiment, using which the probability of differential expression per gene was estimated after accounting for the dropouts. The resulting gene-specific *p*-values were rescaled to control for false discovery rate to deal with the problem of multiple hypothesis testing. Genes that were identified as significantly differentially expressed in a given mouse experiment (Mouse 1, Mouse 2, and Mouse 3) by either the SCDE or KNN model were prioritized for assessment across both mice. This resulted in 71, 606, and 18 differentially expressed genes in Mouse 1, Mouse 2, and Mouse 3 respectively. We performed pathway enrichment analysis on these lists of genes from individual mice using the National Cancer Institute’s Pathway Interaction Database^58^, a curated collection of known biomolecular interactions and key signaling pathways associated with cancer, to evaluate if genes belonging to specific cancer-related pathways were enriched within the individual gene lists, followed by an assessment of false discovery rate using the Benjamini-Hochberg^59^ methodology. A total of 14 genes were found to be commonly differentially expressed in both PDX models (Mouse 1, Mouse 2) in addition to 10 commonly enriched pathways. The scRNASeq data have been deposited in the National Center for Biotechnology Information Sequence Read Archive (SRA) and are accessible under the Series accession number PRJNA706068.

### Gene set enrichment analysis

Significantly differentially expressed genes from RNA sequencing of MDA-MB-231 wild-type and ICAM1 knockdown cells were analyzed using GSEA. Analysis was performed using a rank-ordered list of differentially expressed genes on the C2 Chemical and Genetic Perturbations curated gene set collection. Analysis was performed using GSEA software version 4.0.3 and MSigDB gene set collections version 7.1^60,61^.

### Breast cancer patient blood sample collection

The blood sample collection from stage III–IV breast cancer patients was permitted by the Institutional Review Board at Northwestern University and complied with NIH guidelines for human subject studies. Blood samples were collected in collected in CellSave preservative tubes for CellSearch platform analyses and in EDTA tubes for flow cytometry analyses. CellSearch kit and anti-ICAM1 antibody (conjugated to PE, BD# 555511) were used to enrich CTCs for immunofluorescence staining. Live blood cell samples were centrifuged and after red blood cell lysis (lysis buffer Sigma cat# R7757), white blood cells were stained with antibodies for lineage markers such as CD45 (leukocytes), EpCAM (epithelial), and candidate markers ICAM1 and CD44 for flow cytometry analysis on FACS LSR (BD Biosciences). Single and clustered tumor cells were gated for ICAM1 expression (%). In some sample processing, CD45^+^ PBMCs were depleted using the kit (Miltenyi Biotec Depletion column cat#130-042-901).

### Flow cytometry and cell sorting

Dissociated PDX tumor cells or MDA-MB-231 cells were resuspended at 10 million per mL in PBS with 2% FBS. Cells were first blocked with IgG for 10 min, then incubated with mouse anti-human ICAM1-APC antibody (BD Biosciences #559771) for 25 min on ice, followed by washing twice with PBS. Finally, cells were diluted in PBS and filtered, prior to analysis on a BD-LSR II flow cytometer (BD Biosciences) or a BDAria cell sorter (BD Biosciences). Flow cytometry data and dot plots have been analyzed by BDAria v8.0.2 or Flow Jo v.

### Western blotting

Cells were lysed by RIPA buffer with protease inhibitor cocktail (1:100 dilution) for 30 min on ice, then centrifuged for 15 min at 4 °C and 10,000×*g*. Equal amounts of protein of each sample (20–40 µg) were subjected to SDS-PAGE, then transferred to nitrocellulose membranes. The primary antibodies used include: ICAM1 (1:3000, Sigma Aldrich, HPA004877), CDK6 (1:1000, Protein Tech, 14052-1-AP), CD44 (1:1000, Thermo Fisher, 156-3C11), EpCAM (1:1000, Thermo Fisher, MA1-10195), Oct3/4 (1:1000, Santa Cruz, sc-5279), c-Myc (1:1000, Thermo Fisher, 13-2500), Flag (1:1000, Sigma-Aldrich, F7425), Sec23a (1:5000, Thermo Fisher, PA5-28984), HIF1a (1:2000, Thermofisher, MA1-516), BMP4 (1:500, Protein Tech, 12492-1-AP), HMGA2 (1:2000, Protein Tech, 20795-1-AP), CD34 (1:500, Protein Tech, 14486-1-AP), N Cadherin (1:4000, Protein Tech, 22018-1-AP), PAWR (1:500, Protein Tech, 20688-1-AP), PAI1 (1:500, Protein Tech, 13801-1-AP), KRT19 (1:2000, Protein Tech, 10712-1-AP), MCM3 (1:2000, Protein Tech, 15597-1-AP), ANKRD1 (1:3000, Protein Tech 11427-1-AP), Notch1 (1:1000, Cell signaling 3608 S), and β-actin (1:1000, Abcam ab8224). Zeb1 antibody was a gift from Dr. Hidayatullah G. Munshi (Northwestern University). The horseradish peroxidase (HRP)-conjugated secondary antibodies used were from Promega (1:10,000, Rabbit W401B and Mouse W402B), and the substrate ECL was detected by Pierce ECL2 solution (Thermo Fisher Scientific, 1896433A). The full blot images of Western blots have been provided in a Supplementary document.

### Immunohistochemistry

Mouse xenograft primary tumors and lung tissues were paraffin-embedded and sectioned by routine techniques. Deparaffinization and rehydration of tissue sections were first achieved. Heat-induced antigen retrieval was done using Decloaker solution for 15–20 min (Biocare Medical, RD913L). Tissue sections were blocked with TBS/10% NGS, then incubated with ICAM1 primary antibody overnight (Sigma Aldrich, HPA004877), followed by Dako envision plus kit and DAB staining. All samples were counterstained with hematoxylin.

### Immunofluorescence

MDA-MB-231 cells were allowed to cluster in suspension in Poly-HEMA coated plates for 24 h. Cells were then collected and spun onto Cell-Tak (Corning) coated cover slides, and fixed with 4% paraformaldehyde for 10 min. Cells were permeabilized using 0.25% Triton X-100 in PBS, followed by blocking with 2% BSA in PBS for 1 h. ICAM1 primary antibody was then incubated with cells overnight at 4 °C. Cells were then washed with PBS and incubated with Alexa 568-conjugated secondary antibody (Thermo Fisher) for 1 h, and finally nuclei were counterstained with DAPI.

### Cell clustering assay

Two different types of cells (PDXs and cell lines) and protocols (collagen-I coated and poly-HEMA treated plates) were used to analyze the tumor cell clustering potential. Primary tumor cells in single-cell suspension were dissociated from PDX models (which were only propagated and maintained in mice) and seeded in 96-well plates coated with collagen type I (which enabled a temporary survival of PDX primary tumor cells with very loose attachment but mobile capability ex vivo for up to one week). MDA-MB-231 cells with a strong adhesive phenotype were trypsinized into single cells first, and then seeded in suspension in 96-well plates pretreated with poly-hydroxyethyl methacrylate (Poly-HEMA, Sigma-Aldrich), which prevented cell attachment to the plate and keeps cells in suspension. The cells were then incubated and monitored by the IncuCyte live cell imaging system (Essen BioScience), and images were acquired every 2 h. Cluster size was analyzed over time by the IncuCyte ZOOM software. In addition, MDA-MB-231 tumor cells were incubated with anti-ICAM1 neutralizing antibody (R&D Systems, AF720) in DMEM/2% FBS media and imaged using the same experimental method.

### Co-immunoprecipitation

For ICAM1 overexpression in HEK-293 cells, the cells were trypsinized after transfection and seeded on pretreated Poly-HEMA-coated 10 cm plates for 3–6 h to form aggregates in suspension. Collected aggregates were then lysed in Pierce IP lysis buffer (Thermo Fisher) with protease inhibitor cocktail for 30 min on ice. For co-immunoprecipitation, anti-Flag magnetic beads were added into samples and incubated overnight at 4 °C. The beads were washed 3–4 times with washing buffer (0.1% TBS-T with 1% Triton-100X), and binding proteins were eluted with 0.1 M glycine (PH 2-3) for 5–10 min, then neutralized with added TBS.

### Structural modeling

The monomeric structure of ICAM1’s (UniProt ID: P05362) ExD (aa 28–477) was retrieved from the Protein Data Bank (PDB ID: 1Z7Z; Chain I). The homodimer models were initially built with rigid docking by the ClusPro webserver^39^ (dimer mode) and then flexible refinement by BAL (Bayesian Active Learning)^38^, a machine learning-assisted protein docking method with uncertainty quantification. BAL predicted not only the refined dimer structure models but also the conditional probabilities of individual models (summing up to 1), interfacial residue-contacts, and interfacial residues.

### Solid-phase homophilic interaction assay

ICAM1 ExD protein (Sino Biological) was denatured before coating and biotinylation to dissociate possibly aggregated ICAM1 molecules. Purified ICAM1 was coated overnight in a high binding EIA/RIA microplate (ICAM1; 1 µg/well). Blocking buffer with 5% BSA/TBS was added the following day for 2 h at room temperature, prior to adding biotin-labeled ICAM1 or BSA (EZ-Link Sulfo-NHS-LC-Biotinylation Kit, Thermo Scientific) in binding buffer (TBS, 0.1% BSA, 1 mM MgCl_2_, 1 mM CaCl_2_) and kept overnight. Bound biotin-labeled ICAM1 was detected with streptavidin-HRP (Thermo Scientific) and quantified upon adding tetramethylbenzidine substrate at 450 nm.

### Mass spectrometry

MDA-MB-231 tumor cell pellets were collected after transfection with either control or ICAM1 siRNAs, and then sent to the Case Western Proteomics Core facility for cell lysis with 2% SDS and protease inhibitor cocktail and physical protein extraction using pulse sonication. In all, 10 µg of sample was digested with LysC/Trypsin, and 300 ng was analyzed via 4 h LC/MS/MS. Data were processed and quantified using Scaffold and PEAKS. Among 1827 proteins identified with two minimal peptides and confidence of 95% at the protein levels and 99% at the peptide level, a total of 170 peptides mapping to 76 proteins passed the filters (*P* value of 0.05 and a minimum fold change of 1.5) for quantification of total unique spectrum counts. The label-free mass spectrometry raw data files have been deposited to jPOST (https://repository.jpostdb.org/entry/JPST001184)^62^ with accession number JPST001184.0 and PXD026234.

### RNA sequencing

Total RNA extraction of MDA-MB-231 cells was isolated using Trizol, phase separated by chloroform, and extracted by alcohol. Samples were sent to Northwestern University’s Center for Genetic Medicine Sequencing core facility for deep sequencing analysis. RNA sequencing was performed on a HiSeq 4000, and a library was made using a TruSeq Total RNA-Seq Library Prep kit. Data were processed and quantified using STAR^63^, DESeq2^64^, and HTSeq^65^. Analysis of differentially expressed genes was set to a cutoff of FDR < 0.05 and Log2 (Fold Change) > 0.48 or <−0.48. Finally, the pathway analysis of significantly differentially expressed genes was obtained using Metascape (http://metascape.org)^66^.

### Cell cycle analysis

Cells were fixed with 70% alcohol after collection, then washed with PBS and incubated with RNAse A for 1 h, and later propidium iodide dye was added. Samples were kept at 4 °C in the dark, until flow cytometry analysis on an LSRII instrument.

### Mammosphere assay

Tumor cells were seeded at a low density of 2000 cells in suspension in a 12-well plate coated with poly-HEMA in PRIME-XV® Tumorsphere serum-free medium (Irvine Scientific, 91130). The total number of mammospheres (diameter >50 µm) was counted for each well after 5–10 days.

### Scratch wound assay

Tumor cells were seeded in an image-locked 96-well plate overnight. On the following day after cells became confluent, a scratch was made using the IncuCyte wound maker. Floating cells were washed with PBS, and fresh culture media was added to the remaining adherent cells. The cells were then incubated in the IncuCyte for real-time imaging of tumor cell migration and filling the wound.

### Transendothelial migration

We started by coating the transwell inserts of a 24-well plate with collagen type I for 1 h, followed by fibronectin (10 µg/mL) for 10 min, and finally seeding HUVECs in the upper insert. When HUVECs formed a confluent monolayer (24–48 h later), MDA-MB-231 tumor cells were added to the top insert with serum-free medium (1/4 EBM2 + ¾ DMEM serum free), and media with serum was added to the lower chamber (1/4 EBM2 + 3/4 DMEM with 10% FBS). The tumor cells that transmigrated to the bottom chamber were collected 24 h later, and centrifuged at 4 °C and 300×*g*. Finally, the transmigrated live cells were counted with Trypan blue staining using a hemocytometer. In addition, MDA-MB-231 tumor cells were incubated with anti-ICAM1 neutralizing antibody (R&D Systems, AF720), then added directly to the top insert to block ICAM1 on both tumor cells and endothelial cells.

### ICAM1 expression association with PAM50 subtypes

The TCGA BRCA Cohort data was acquired from the GDC Pan-Cancer Analysis Project^67^ and the PAM50 subtype annotations were obtained from^68^.

### ICAM1 expression association with patient survival

Affymetrix Human Genome U133A Array data for HER2-negative breast cancer cases treated pre-operatively with taxane-anthracycline chemotherapy were downloaded from Gene Expression Omnibus (GEO; GSE25055; *N* = 310). Similarly, gene expression data from breast cancer tissue in a large population-based cohort of Swedish patients was also downloaded from GEO (GSE1456-GPL96; *N* = 159). The GSE25055 and GSE1456 data referenced during the study are available in a public repository from the Gene Expression Omnibus (https://www.ncbi.nlm.nih.gov/geo/) website. Of note, inflammatory breast cancers were excluded from this analysis. The data were pre-processed with an RMA (Robust multichip averaging) algorithm using the R/Bioconductor package Oligo (Version 1.56)^69^, where background subtraction, quantile normalization, and summarization (via median-polish) were accomplished. ICAM1 expression was obtained for each sample, and patients within each cohort were divided into two groups according to the median expression level of ICAM1. In addition, a 98-gene stemness signature (Supplementary Table 4) was evaluated on a per-sample basis using the single sample Gene Set Enrichment Analysis (ssGSEA) (Version 9) protocol^60,70^], thus providing a Stemness Signature Index per patient tumor. Patients were then divided into groups based on whether their tumors express higher than median levels of ICAM1 expression either alone or in combination with the Stemness Signature Index. Survival differences between these groups were estimated using the Survival package in R^71^ and the Kaplan–Meier survival plots were created using the ggsurvplot command from the Survminer package (Version 0.4.9) in R (Version 3.7)^72^.

### Statistical analysis

Microsoft Excel was used to perform Student’s *t* test and calculate *P* values for all in vitro assays and analyses unless specified otherwise. *P* ≤ 0.05 was considered statistically significant and is represented with one asterisk (*). *P* ≤ 0.01 is represented with two asterisks (**). Likewise, ***P ≤ 0.001 and ****P ≤ 0.0001. Data are presented as mean ± standard deviation (SD) unless specified otherwise. In the box plots, the central line median values are presented.

### Reporting summary

Further information on research design is available in the Nature Research Reporting Summary linked to this article.

## Supplementary information

Supplementary Information

Reporting Summary

## Data Availability

The PDX single-cell and MDA-MB-231 cell RNA sequencing data have been deposited in the National Center for Biotechnology Information Sequence Read Archive (SRA) database under the accession code PRJNA706068, currently released). The GSE25055 and GSE1456 data referenced during the study are available in a public repository from the Gene Expression Omnibus (https://www.ncbi.nlm.nih.gov/geo/) website. The label-free mass spectrometry raw data files have been deposited to jPOST (https://repository.jpostdb.org/entry/JPST001184) with accession number JPST001184.0 and PXD026234 (publicly available). The full blot images of Figs. 2j, l and 3c and Supplementary Figs. 3f, 4c, 5b, 6c, f, 7a, 8e, and 9a are provided in a Supplementary Source Data file. All the other data supporting the findings of this study are available within the article and its supplementary information files and from the corresponding author upon reasonable request. A reporting summary for this article is available as a Supplementary Information file. Source data are provided with this paper.

## Source data

Source Data
