# Supplementary material for: ICAM1 initiates CTC cluster formation and trans-endothelial migration in lung metastasis of breast cancer
Source: Nat Commun. 2021 Aug 11;12:4867. doi: 10.1038/s41467-021-25189-z (PMC8358026; doi:10.1038/s41467-021-25189-z)
Supplement: Supplementary file 1 — Supplementary Information [file 41467_2021_25189_MOESM1_ESM.pdf]

ICAM1 initiates CTC cluster formation and trans-endothelial migration in lung metastasis of breast cancer

Rokana Taftaf[1,2*], Xia Liu[1,3*], Salendra Singh[4], Yuzhi Jia[1], Nurmaa K. Dashzeveg[1], Andrew D. Hoffmann[1], Lamiaa K. M. A. El-Shennawy[1], Erika K. Ramos[1,2], Valery Adorno-Cruz[1], Emma J. Schuster[1,2], David Scholten[1,2], Dhwani Patel[1], Youbin Zhang[5], Andrew A. Davis[5,6], Carolina Reduzzi[5], Yue Cao[7], Paolo D'Amico[5], Yang Shen[7], Massimo Cristofanilli[5,8], William A Muller[8,9], Vinay Varadan[4#], Huiping Liu[1,5,8#]

[1]Department of Pharmacology, [2]Driskill Graduate Program in Life Sciences, Northwestern University Feinberg School of Medicine, Chicago, IL, USA. [3]Department of Toxicology and Cancer Biology, University of Kentucky, KY, USA. [4]Case Comprehensive Cancer Center, Case Western Reserve University, Cleveland, OH, USA. [5]Department of Medicine, the Division of Hematology and Oncology, Northwestern University Feinberg School of Medicine, Chicago, IL, USA. [6]Division of Hematology and Oncology, Department of Medicine, Washington University in St. Louis, St. Louis, Missouri. [7]Department of Electrical and Computer Engineering, TEES-AgriLife Center for Bioinformatics and Genomic Systems Engineering, Texas A&M University, College Station, TX 77843. [8]Lurie Comprehensive Cancer Center, Northwestern University Feinberg School of Medicine, Chicago, IL, USA. [9]Department of Pathology, Northwestern University Feinberg School of Medicine, Chicago, IL, USA.

* These authors contributed equally. [#]These authors jointly supervised this work.

Corresponding author: Huiping Liu, MD, PhD, Northwestern University, 303 E Superior St, Chicago, IL 60611. Email: huiping.liu@northwestern.edu.
Keywords: ICAM1, CTC clusters, TNBC, metastasis

Supplementary Information and Source Data

1. Supplementary Figures 1-10
2. Supplementary Tables 1-4

Supplementary Fig. 1

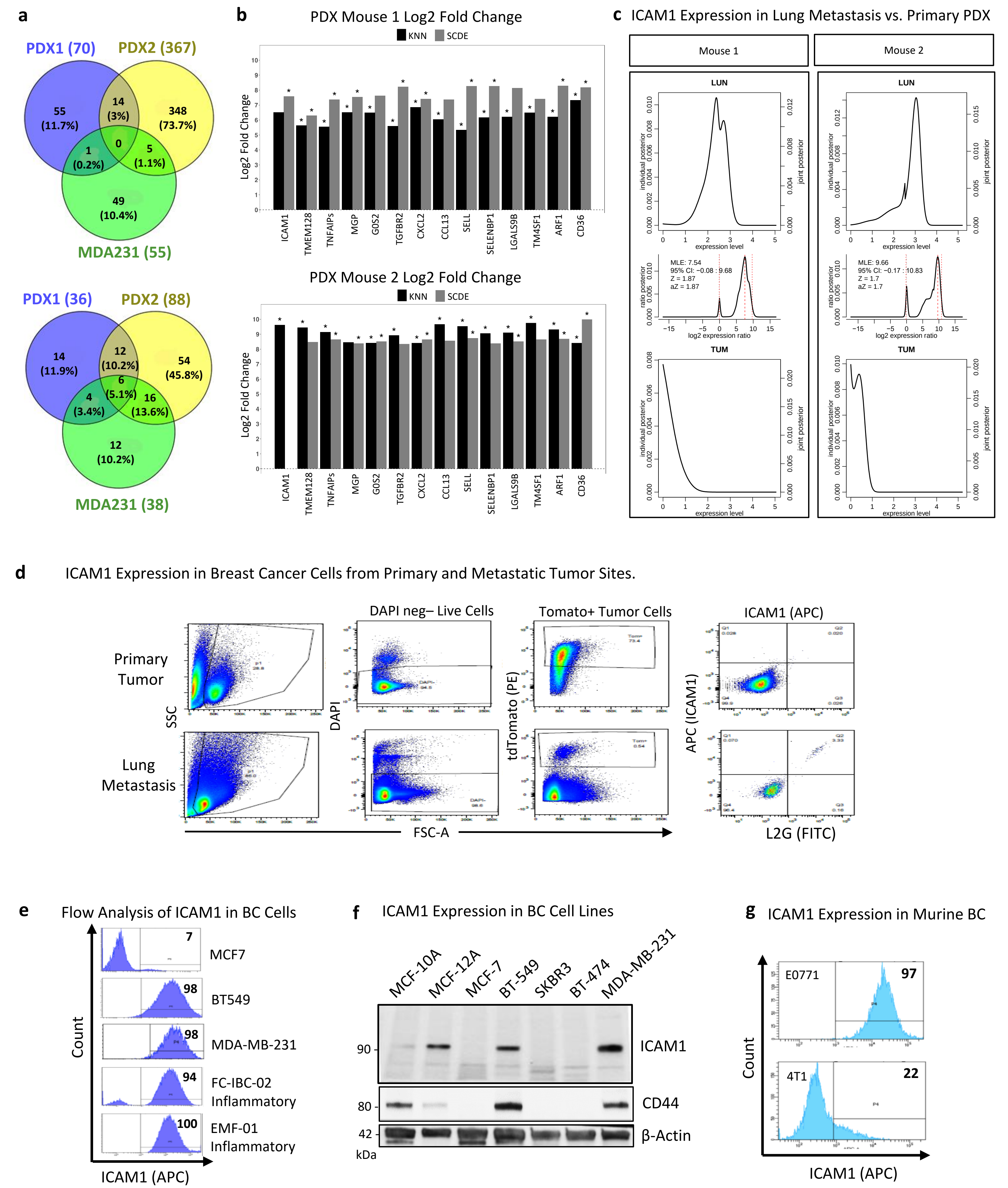

Supplementary Fig. 1. ICAM1 is highly expressed in TNBC (related to Fig 1).

a. Venn diagrams of the significantly differentially expressed genes in 3 paired comparisons between lung metastases and primary tumors for 3 mice (TN PDX in mice 1 and 2, MDA-MB-231 tumor in mouse 3), identified by KNN (top) and SCDE (bottom) platform analyses of single-cell RNA sequencing data.
b. Differential expression levels of the significantly differentially expressed genes in lung metastases compared to primary tumor cells in PDX mouse 1 and mouse 2. Bars denote log2 fold-change estimated by the SCDE (grey) and KNN (black) models. Stars denote statistically significant fold-change (*$P<0.05$, an FDR Corrected 2-tailed p-value from the SCDE pipeline).
c. ICAM1 expression in the lung metastatic cells vs. primary tumor cells of two PDX-bearing mice by single-cell RNA sequencing.
d. Representative flow cytometry gating of PDX L2T+ primary tumor and lung metastasis.
e. ICAM1 expression levels in human TNBC cell lines (top four panels) and MCF7 cells by flow cytometry.
f. ICAM1 and CD44 expression in different breast cancer subtypes by western blotting. N = 1 independent experiment.
g. ICAM1 expression levels in murine TNBC cell lines by flow cytometry.

Supplementary Fig. 2.

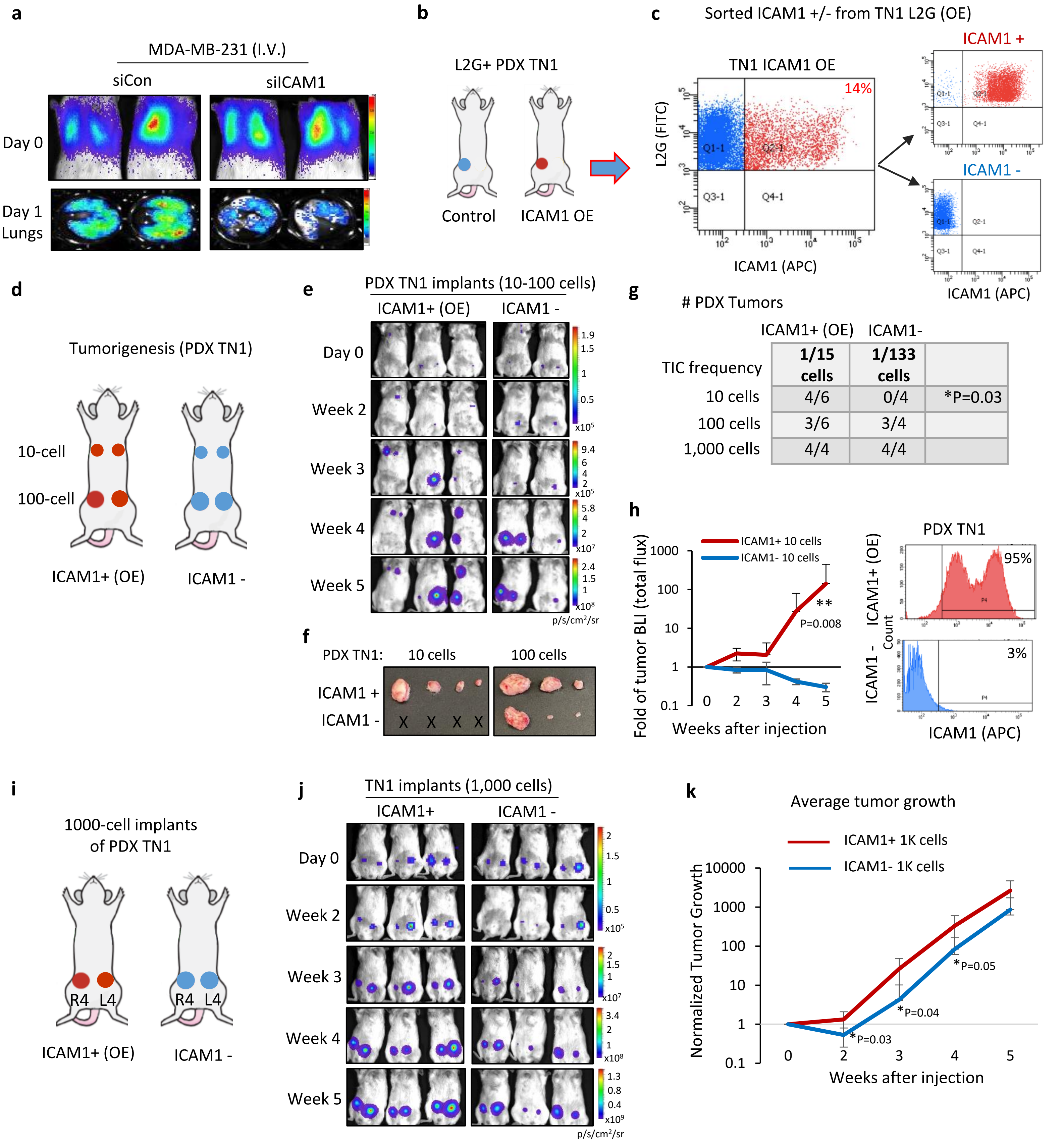

Supplementary Fig. 2. ICAM1 promotes breast cancer cell growth and metastasis (related to Fig 1).

a. Representative BLI images of mice injected with siCon and siICAM1-transfected MDA-MB-231 cells via tail vein on day 0 and 1 (dissected lungs *ex vivo*).

b. Schematic of orthotopic implants of TN1 PDX cells control (blue) and ICAM1 overexpression (OE; red) at 4th mammary fat pads of NSG mice.

c. Flow sorting of $ICAM1^+$ and $ICAM^-$ cells from ICAM1-overexpressing (OE) $L2G^+$ TN1 PDXs.

d-k. Tumorigenic assays with sorted $ICAM1^+$ and $ICAM1^-$ cells in serial dilutions of 10-100 cells (d-h) and 1000 cells (i-k) per injection at the 2nd and 4th mammary fat pads, BLI images of PDX TN1 tumors from day 0 (D0) to 5 weeks (Wk5) post implantation (e and j), table of the tumors grown from each dilution (g), and normalized tumor growth curves of tumor cells (h and k) (N= 4-6 injections per cell group). (h) also confirms the ICAM1 expression levels in the tumors grown from sorted $ICAM1^+$ and $ICAM1^-$ cells. Data are presented as mean values +/- SD. Error bars represent SD values. (h) Two-sided *t* test **P=0.008. (k) Two-sided *t* test *P=0.03; *P=0.04; *P=0.05)

Supplementary Fig. 3

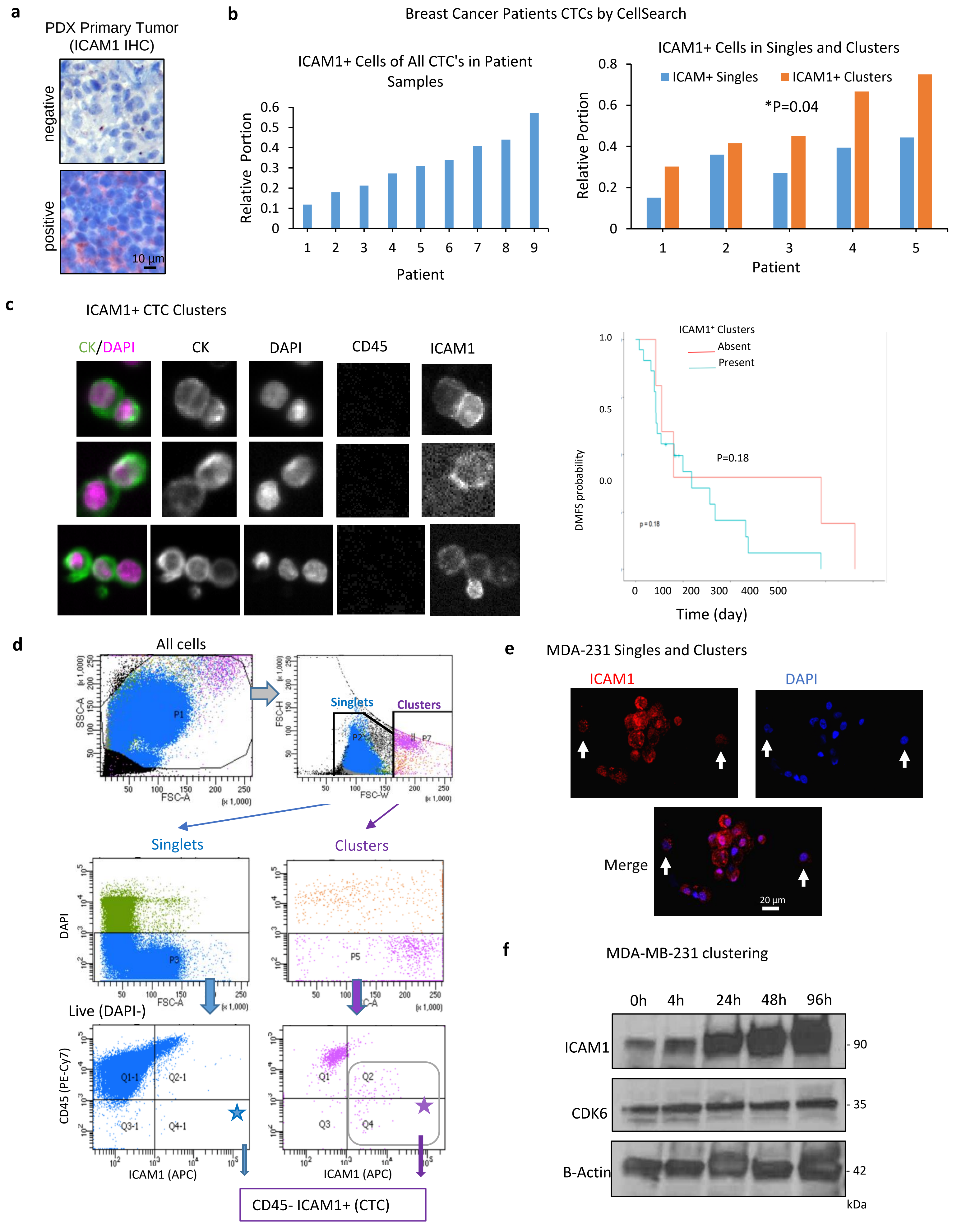

Supplementary Fig. 3. ICAM1 is highly expressed in CTC clusters (related to Fig 2).

a. Negative and positive controls of ICAM1 IHC staining in PDX primary tumors. Minimal 3 sets of the images have shown similar patterns.
b. CellSearch-based ICAM1 expression in CTCs (left panel) and in CTC clusters versus singles (right panel) in breast cancer patients, one-sided *t* test *P = 0.04.
c. Left panel: CellSearch-analyzed representative images of $ICAM1^+$ CTC clusters, green=cytokeratin, purple = DAPI and gray = ICAM1, CD45 negative staining. Right panel: KM-plot in DMFS of breast cancer patients with detectable (present) or absent ICAM1+ CTC clusters analyzed in Fig 2C (N = 50) with a log rank p = 0.18.
d. Representative flow cytometry gating of human $CD45^-$ $ICAM1^+$ CTCs. Side scatters were used to gate singlets and clusters (left two panels) followed by live cell gating (DAPI-) and CD45-ICAM1+ CTCs for both singlets (Q4-1 blue star*) and clusters (rectangle gate purple star*).
e. ICAM1 immunofluorescence of MDA-MB-231 cells during 24 h aggregation assay. Aggregated MDA-MB-231 cells show high expression of ICAM1 at the cytoplasmic membrane. In contrast, the single cells (white arrows) display low levels of ICAM1. Red=ICAM1, blue=DAPI staining. N= 2 independent experiments.
f. Immunoblots of ICAM1 and CDK6 protein levels in clustering MDA-MB-231 cells over time. N = 1 independent experiment.

Supplementary Fig. 4

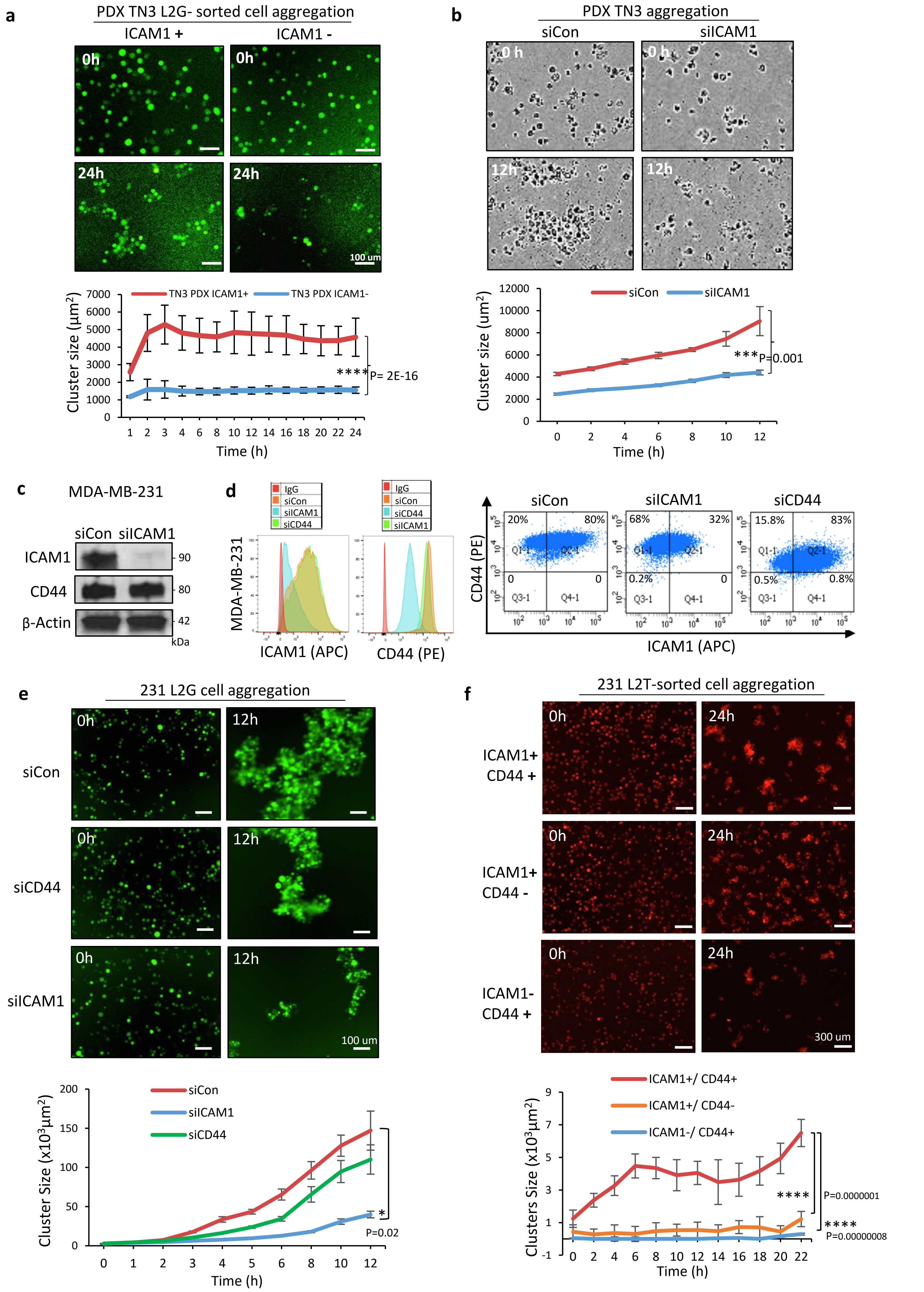

Supplementary Fig. 4. ICAM1 mediates tumor cell clustering independent of CD44 (related to Fig 2).

a. Images (top panels) and quantified cluster sizes (bottom panel) of $ICAM1^+$ and $ICAM1^-$ tumor cells sorted from the PDX TN3 model, and loosely attached for aggregation assays on collagen I-coated plates *ex vivo* (n=8 biological replicates. Data are presented as mean values +/- SD. Error bars represent SD values. Two-sided *t* test ****P = 2E-16).
b. Images (top panels) and cluster size (bottom panel) of PDX TN3 cells transfected with siICAM1 and control, captured by time-lapse IncuCyte live cell imaging microscopy (n=10 biological replicates. Data are presented as mean values +/- SEM. Error bars represent SE values. Two-sided *t* test ***P = 0.001).
c. Immunoblots showing reduced ICAM1 expression and stable CD44 protein levels upon *ICAM1* knockdown in MDA-MB-231 cells. N = 2 independent experiments.
d. Flow cytometry histograms (left two panels) and dot plots (right three panels) of ICAM1 and CD44 expression levels in MDA-MB-231 cells transfected with siICAM1 and siCD44, respectively.
e. Time-lapse aggregation images at 0 and 12 hours (top panels) and cluster size (bottom panel) of L2G MDA-MB-231 cells transfected with siRNA controls (siCon), siCD44, and siICAM1. ICAM1 knockdown had more profound effects than CD44 knockdown on inhibiting the cluster formation (n = 10 biological replicates, Data are presented as mean values +/- SD. Error bars represent SD values. Two-sided *t* test *P=0.02. N = 2 independent experiments)
f. Time-lapse aggregation images at 0 and 24 hours (top panels) and cluster size (bottom panel) of MDA-MB-231 cells sorted based on double positive or single positive cells with ICAM1 and CD44 expression, showing the most efficient cluster formation of double positive cells. Top row, sorted $ICAM1^+CD44^+$double positive cells; middle row, $ICAM1^+CD44^–$; bottom row, $ICAM1^-CD44^+$ cells. (n= 4 biological replicates, Data are presented as mean values +/- SD. Error bars represent SD values. Two-sided *t* test ****P=0.0000001; ****P=0.00000008. N = 1 independent experiment).

Supplementary Fig. 5

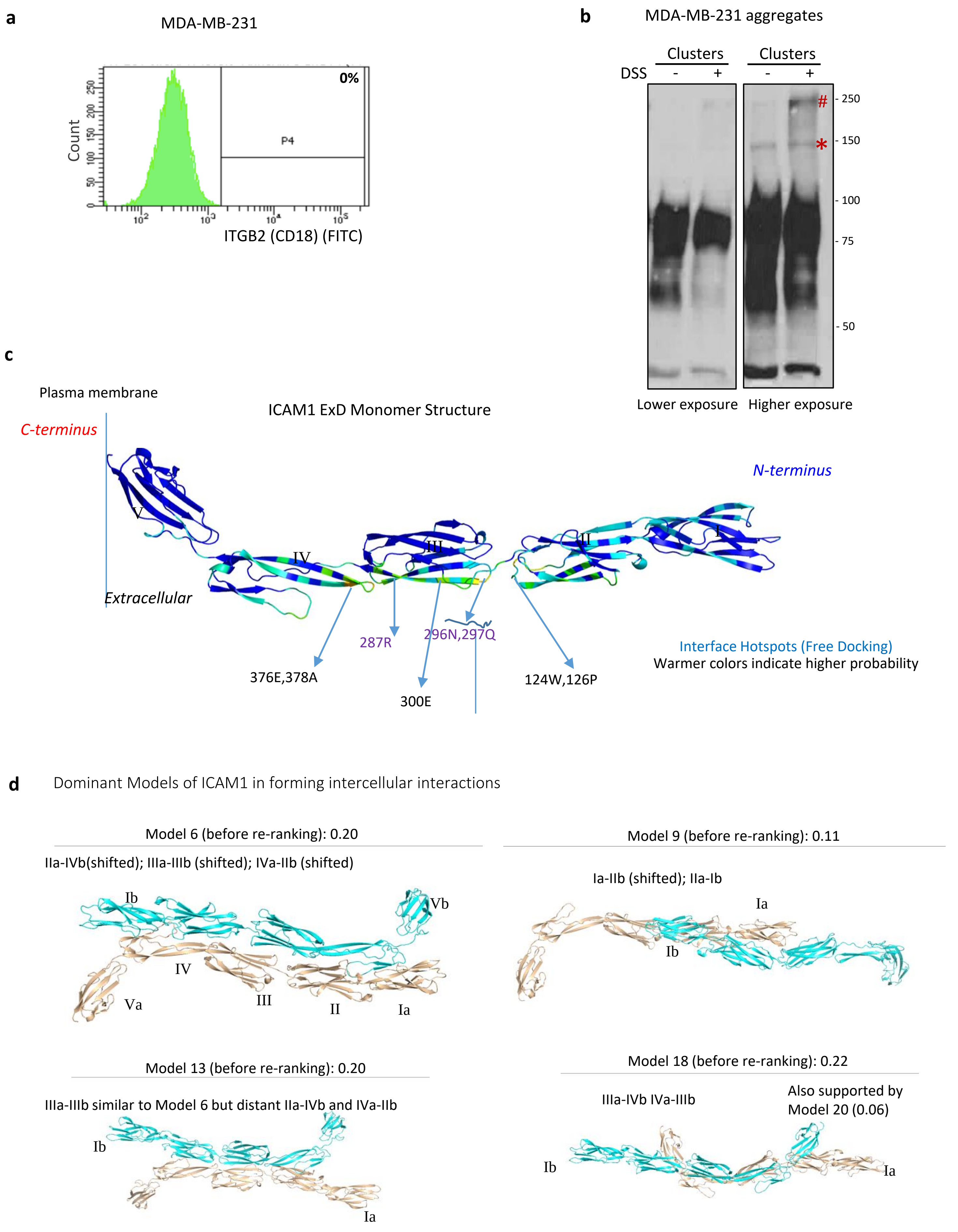

Supplementary Fig. 5. ICAM1 mediates tumor cell clustering through ICAM1-ICAM1 homophilic binding (related to Fig. 2).

a. Flow cytometry of integrin beta-2 (CD18), a beta subunit for LFA-1 and MAC-1, which is not expressed in MDA-MB-231 cells.
b. ICAM1 dimers ~140 kD (*) and possible tetramers (~280 kD) (#) after treatment with the crosslinking agent DSS (left and right panels: lower and higher exposure time of film processing, respectively). N= 2 independent experiments.
c. ICAM1 extracellular domain (ExD) monomer structure with predicted interface hotspots for ICAM1 homodimerization (warmer colors indicate higher probability for binding).
d. Top structural models of ICAM1 homodimerization labeled with conditional probabilities (among 24 total models) and interacting domains.

Supplementary Fig. 6

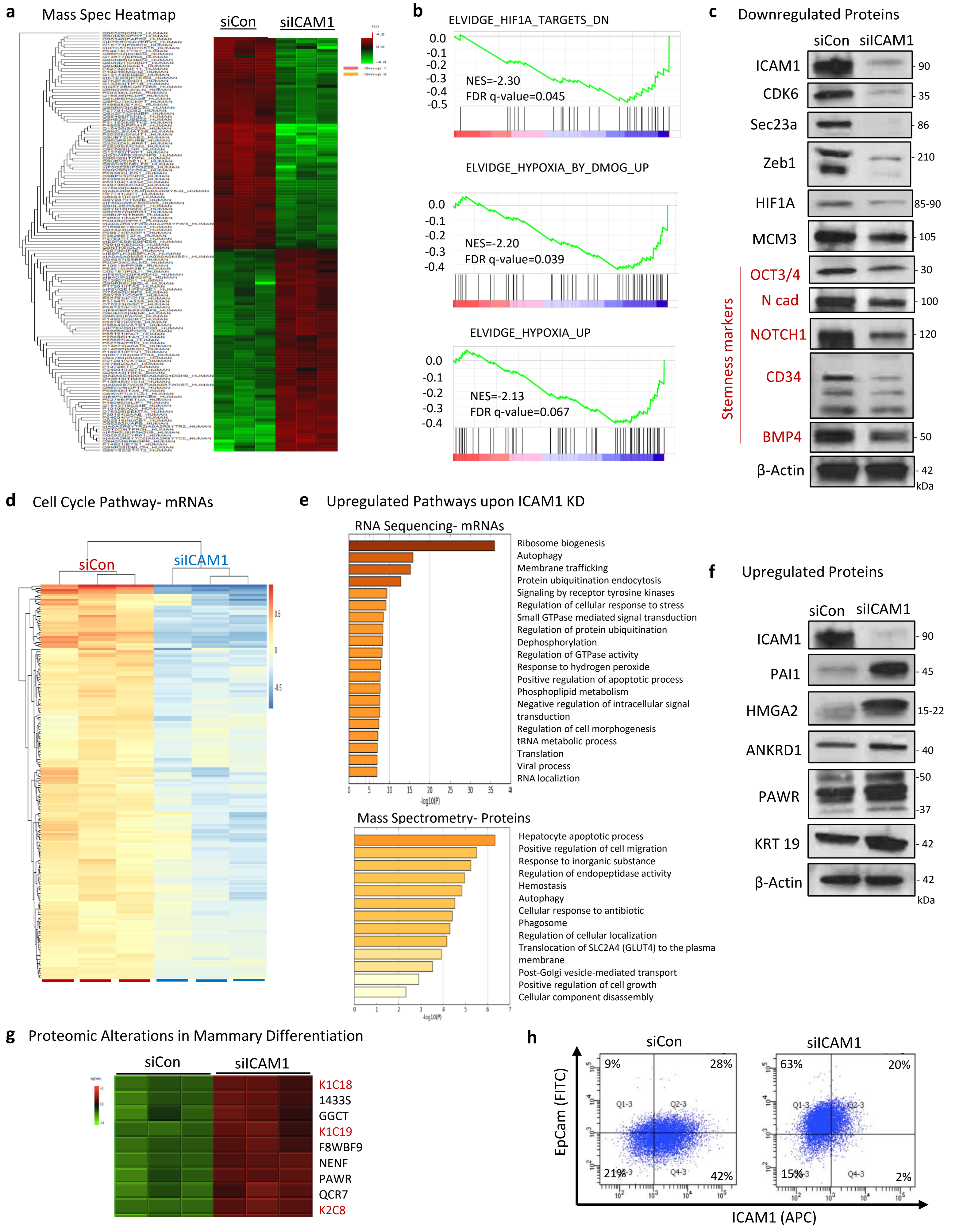

Supplementary Fig. 6. ICAM1 downstream targets and signaling pathways (related to Fig. 3).

a. Heatmap of 76 differentially expressed proteins (>1.5-fold change and two-sided t test $p < 0.05$) upon *ICAM1* knockdown in MDA-MB-231 cells.
b. GSEA of the gene sets for hypoxia and HIF1 targets enriched among the down-regulated genes in MDA-MB-231 *ICAM1* knockdown cells in comparison to siRNA control, identified by RNA sequencing.
c. Immunoblots of ICAM1 targets showing down-regulated proteins and the decrease expression of multiple stemness markers in *ICAM1* knockdown cells. β-Actin serves as a loading control. N= 1-3 independent experiments (3 times for essential targets such as CDK6, Sec23a and Zeb1).
d. Altered cell cycle genes upon *ICAM1* knockdown in MDA-MB-231 cells.
e. Up-regulated pathways upon *ICAM1* knockdown in MDA-MB-231 cells, analyzed by RNA sequencing (top) and mass spectrometry analysis (bottom).
f. Immunoblots of ICAM1 targets showing up-regulated proteins in *ICAM1* knockdown cells. β-Actin serves as a loading control. N= 1 independent experiment.
g. Heatmap of proteomic alterations in mammary epithelial differentiation markers (siCon vs. siICAM1) for mass spectrometry data from three independent experiments.
h. Flow analyses of down-regulated ICAM1 and up-regulated EpCAM levels upon *ICAM1* knockdown.

Supplementary Fig. 7

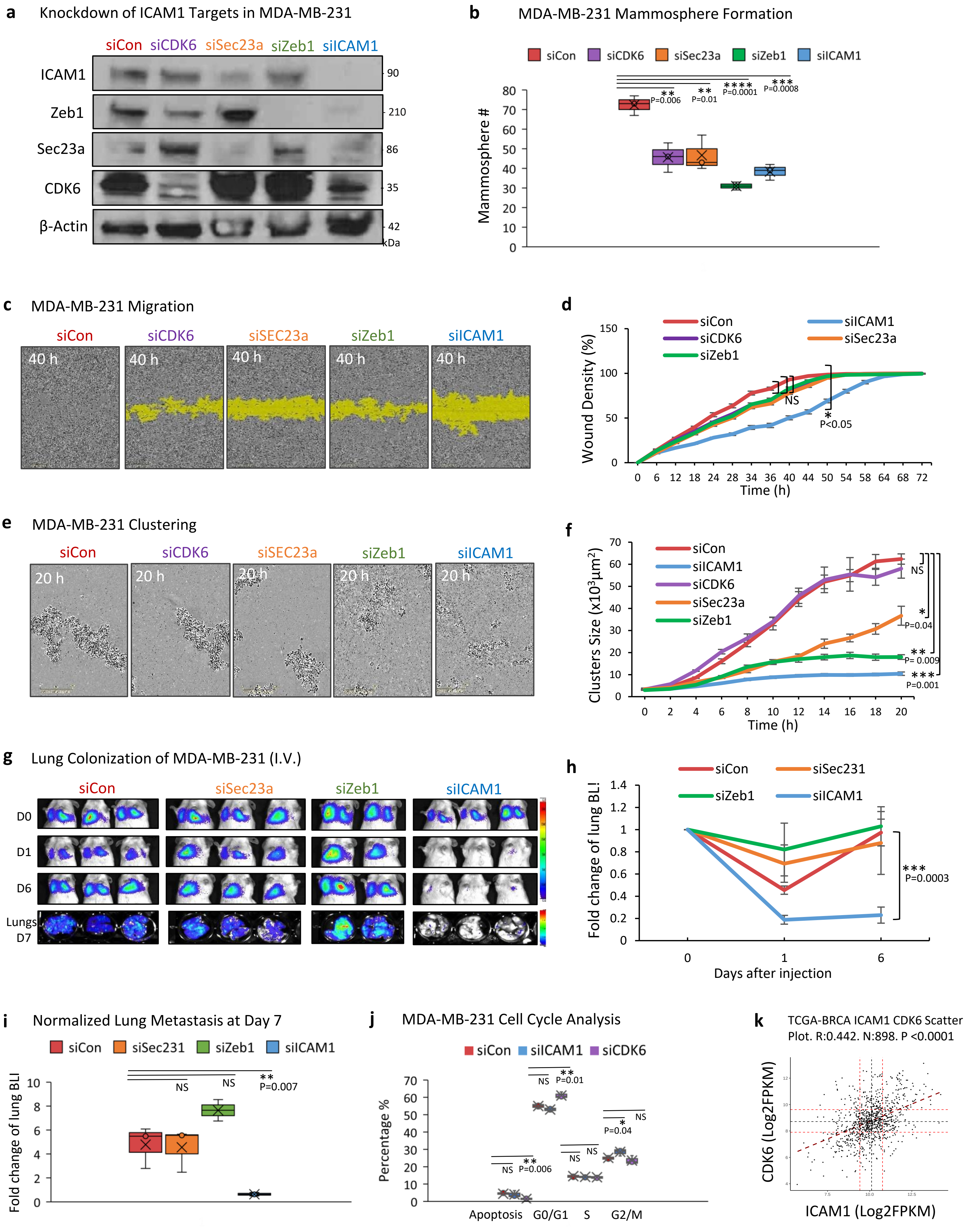

Supplementary Fig. 7. ICAM1 downstream targets partially mimic ICAM1 function (related to Fig 3).

a. Immunoblot validation of the knockdown efficiency of ICAM1, CDK6, Sec23a, and Zeb1 targets in MDA-MB-231. N= 1 independent experiment.

b-f. Effect of knockdown of ICAM1 target genes CDK6, Sec23a, and ZEB1 on tumor mammosphere formation (b), tumor migration (c-d), and tumor cell clustering (e-f), partially mimicking *ICAM1* knockdown (Mammosphere n= 3 biological replicates. Migration n= 10 biological replicates. Clustering n= 10 biological replicates. (b) The boxes range from the first to third quartile with x in a box indicating mean value and whisker lines extending to outliers (minimum and maximum). (d,f) Data are presented as mean values +/- SD. Error bars represent SD values. Two-sided *t* test values shown in b-f. N= 1-4 independent experiments (4 times for essential targets such as ICAM1 and CDK6).

g-i. Representative images (g) and normalized analyses (h-i) of BLI signals of mice injected with MDA-MB-231 cells, which were transfected with siCon, siICAM1, siSec23a, and siZeb1 (n= 3 mice for siCon, siICAM1, siSec23a. n= 2 mice for siZeb1. (h) Data are presented as mean values +/- SD. Error bars represent SD values. Two-sided *t* test ***P=0.0003. (i) two-sided *t* test **P=0.007, NS=not significant. N= 1-4 independent experiments (4 times for essential targets such as ICAM1 and CDK6). (i) The boxes range from the first to third quartile with x in a box indicating mean value and whisker lines extending to outliers (minimum and maximum).

j. Quantification of cell cycle phase percentages using propidium iodide in MDA-MB-231 cells treated with siICAM1 and siCDK6, measured by flow cytometry (n=3 biological replicates. Two-sided *t* test **P=0.006; **P=0.01; *P=0.04. NS= not significant. N= 3 independent experiments). The boxes range from the first to third quartile with x in a box indicating mean value and whisker lines extending to outliers (minimum and maximum).

k. Scatter plot showing positive correlation (Pearson R=0.442; P < 0.0001) between ICAM1 and CDK6 expression in the TCGA breast cancer cohort (N = 898), including all PAM50 subtypes except Normal-Like.

Supplementary Fig. 8

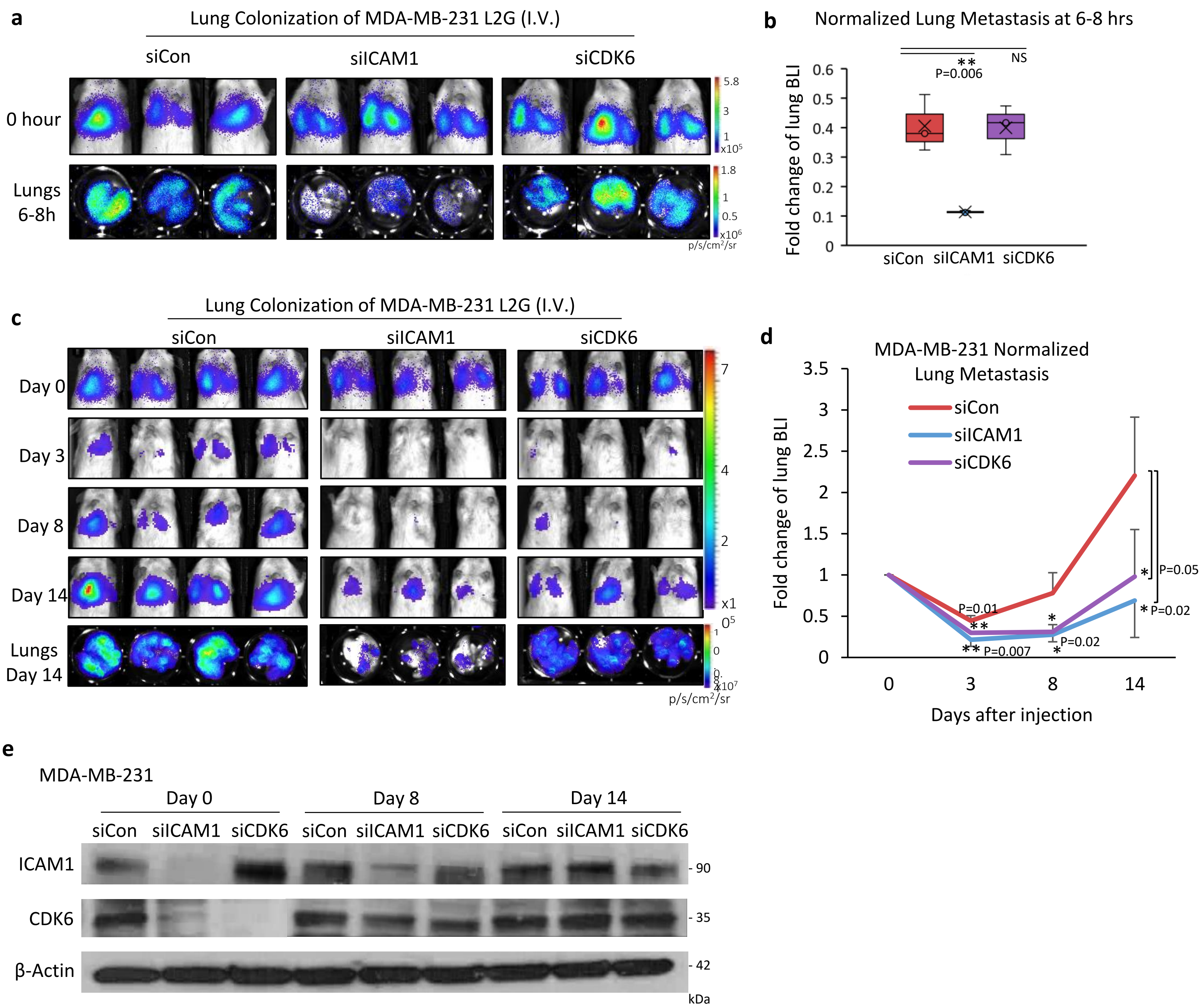

Supplementary Fig. 8. Short-term and long-term effects of gene knockdowns (siICAM1 and siCDK6) on vascular extravasation, metastatic seeding and lung colonization

a-b. Representative images (a) and quantitative data of BLI signals (b) of mice injected with siCon, siICAM1, and siCDK6-transfected MDA-MB-231 cells via tail vein. Lungs were collected 6-8 hours after injection (n=3 mice per cell group. Two-sided *t* test **P=0.006. NS= not significant. N = 4 independent experiments). The boxes range from the first to third quartile with x in a box indicating mean value and whisker lines extending to outliers (minimum and maximum).

c-d. Representative images (d) and quantitative data (e) of BLI signals of mice injected with siCon, siICAM1, and siCDK6-transfected MDA-MB-231 cells via tail vein. Lungs were collected two weeks after injection (n=4 mice for siCon. n= 3 mice for siICAM1, siCDK6. Data are presented as mean values +/- SD. Error bars represent SD values. Two-sided *t* test values are shown. N = 4 independent experiments).

e. Immunoblot validation of the knockdown efficiency of ICAM1 and CDK6 in MDA-MB-231 and their protein level expression over time. N= 1 independent experiment.

Supplementary Fig. 9

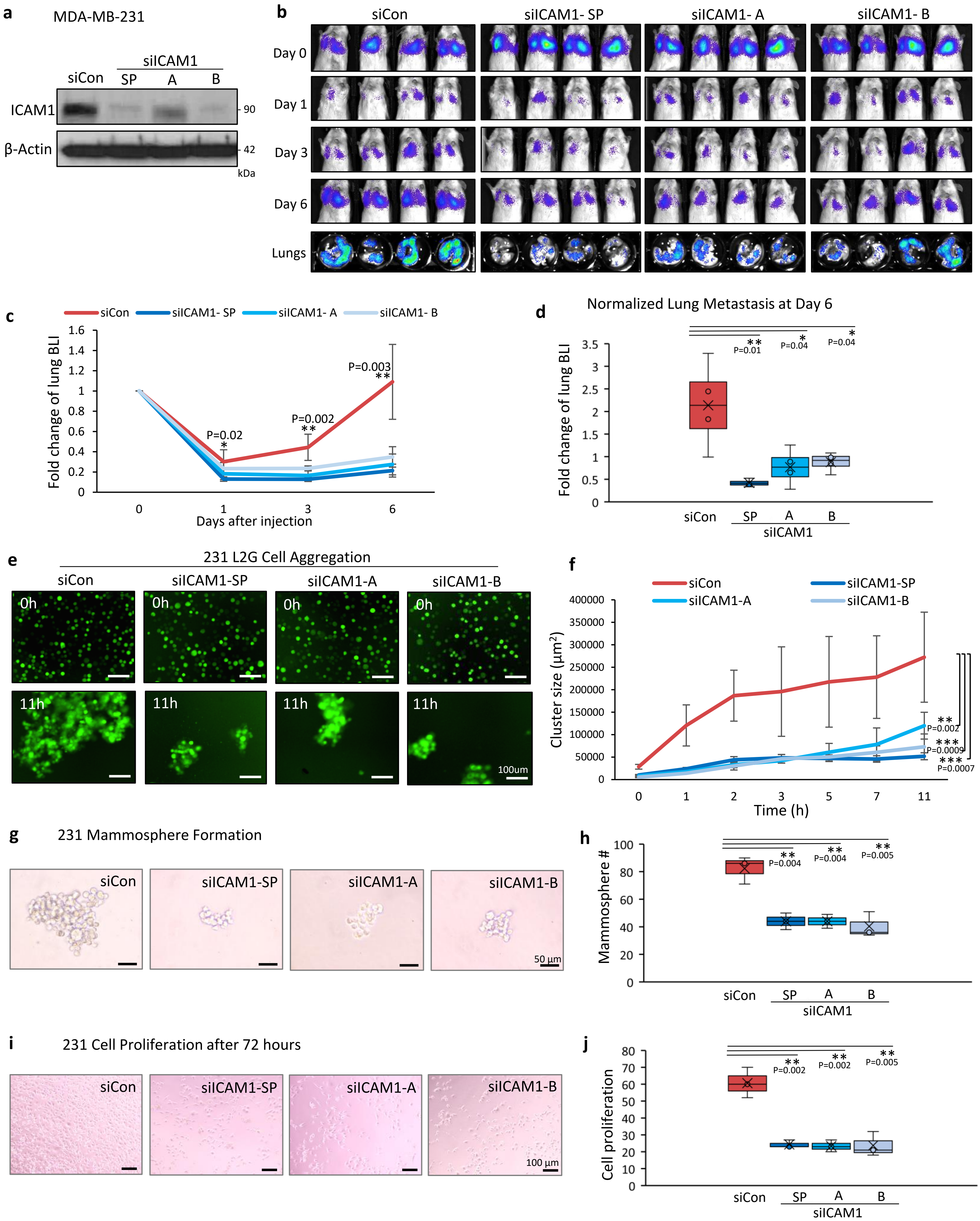

Supplementary Fig. 9. ICAM1 smart pool and individual siRNAs inhibit lung colonization and clustering (related to Fig. 3)

a. Immunoblot validation of the knockdown efficiency of ICAM1 using different ICAM1 siRNAs (Smartpool-SP, Individual-A, Individual-B) in MDA-MB-231 tumor cells. N= 2 independent experiments.

b-d. Representative images (b) and quantitative data (c,d) of BLI signals of mice injected with siCon and different siICAM1 (SP, A, B) -transfected MDA-MB-231 cells via tail vein. (n=4 mice per group. (c) Data are presented as mean values +/- SD. Error bars represent SD values. (d) The boxes range from the first to third quartile with x in a box indicating mean value and whisker lines extending to outliers (minimum and maximum). Two-sided *t* test *P=0.02; **P=0.002; **P=0.003. (d) Two-sided *t* test **P=0.01; *P=0.04; *P = 0.04).

e-j. Effect of knockdown of different ICAM1 siRNAs (SP, A, B) on MDA-MB-231 tumor cell clustering (e-f), tumor mammosphere formation (g-h), and tumor proliferation (i-j). (Clustering n= 10 biological replicates. Mammosphere n= 3 biological replicates. Proliferation n= 3 biological replicates. (f) Data are presented as mean values +/- SD. Error bars represent SD values. (h,j) The boxes range from the first to third quartile and x in a box indicates mean value. Two-sided *t* test values shown in e-j. N = 1-4 independent experiments (4 times for siICAM1-SP).

Supplementary Fig. 10

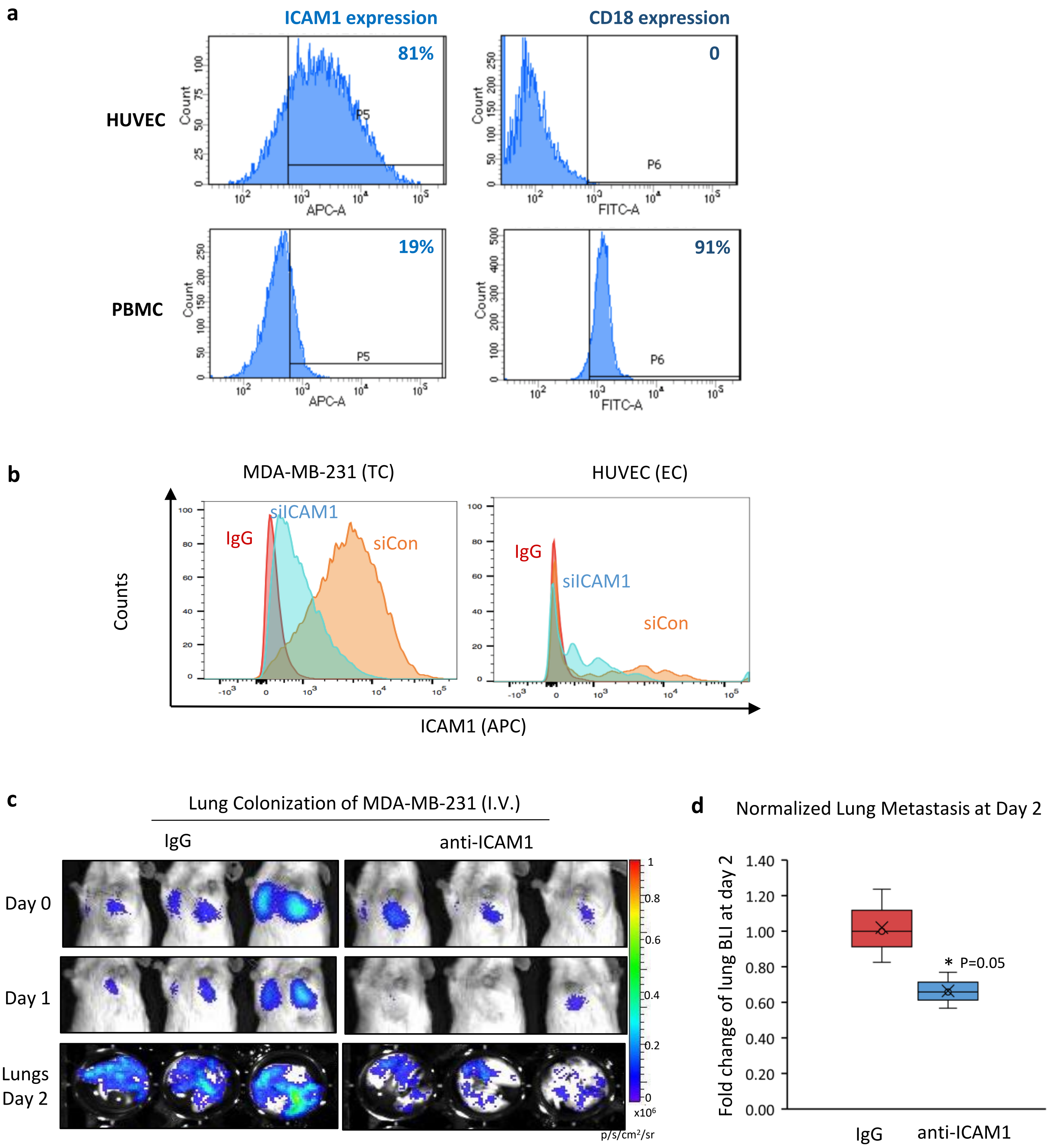

Supplementary Fig. 10. Anti-ICAM1 inhibits lung colonization of TNBC cells (related to Fig. 4)

a. Flow cytometry of ICAM1 and integrin beta-2 (CD18) expression in human HUVECs and PBMC cells.

b. Flow cytometry validation of *ICAM1* knockdowns in breast tumor cells and HUVECs.

c-d. Representative BLI images (c) and quantitative data (d) of mice pretreated with IgG or anti-ICAM1 neutralizing antibody (80ug/mouse) through tail vein, followed 3 hours later with tail vein injection of MDA-MB-231 cells which have been preincubated with IgG or anti-ICAM1 antibody (100ug /1x10^5 cells) for 30min. Lungs were collected 2 days after injection. (n=3 mice per group. Two-sided *t* test *P=0.05. N= 2 independent experiments). The boxes range from the first to third quartile with x in a box indicating mean value and whisker lines extending to outliers (minimum and maximum).

Supplementary. Table 1. LUNG MET SPECIFIC PATHWAYS (related to Fig. 1)

Benjamini-Hochberg's (BH) false discovery rate (FDR) corrected Hypergeometric P-values

	Mouse 1			Mouse 2			Mouse3		
PATHWAY	M1_HyperGeom_PVal	M1_BH	M1_Genes	M2_HyperGeom_PVal	M2_BH	M2_Genes	M3_HyperGeom_PVal	M3_BH	M3_Genes
HIF-1-alpha transcription factor network	0.01513362	0.077043903	NCOA1	0.001064666	0.016532567	ALDOA,CP,LDHA,FOS,ABCG2,PFKL	0.00101922	0.026825267	EGLN3
Canonical NF-kappaB pathway	0.0019306	0.05637933	TNFAIP3	0.017435629	0.073690206	TNFAIP3,TNFRSF1A	-	-	-
Glucocorticoid receptor regulatory network	0.00176703	0.05637933	ICAM1,NCOA1	0.004269218	0.032468243	ICAM1,CDKN1A,IL6,FOS,PPT1,SGK1	-	-	-
Integrin-linked kinase signaling	0.0072568	0.05637933	ZEB1	0.004493373	0.032468243	GIT2,RHOG,LIMS1,TNS1	-	-	-
Regulation of nuclear SMAD2/3 signaling	0.00164899	0.05637933	FOXO3,NCOA1	0.000832729	0.016532567	ATF3,CDK4,CDKN1A,IRF7,FOS,SIN3B,SMA	-	-	-
Thromboxane A2 receptor signaling	0.01222613	0.068466324	ICAM1	0.00298155	0.029037709	ICAM1,VCAM1,TGM2,GNG2,PRKCH	-	-	-
TNF receptor signaling pathway	0.00757283	0.05637933	TNFAIP3	0.000807359	0.016532567	TNFAIP3,MAP3K5,MAP3K3,TNFRSF1A,TX	-	-	-
Validated nuclear estrogen receptor alpha network	0.01513362	0.077043903	NCOA1	0.0052185	0.035422545	C3,CTSD,NEDD8,PCNA,CD82	-	-	-
Validated nuclear estrogen receptor beta network	0.00081307	0.05637933	NCOA1	0.005168398	0.035422545	C3,NEDD8	-	-	-
Validated targets of C-MYC transcriptional repressio	0.01385428	0.075208668	FOXO3	0.004146363	0.032468243	CEBPD,CDKN1A,GBP2,NDRG2,SMAD2	-	-	-
HIF-2-alpha transcription factor network	-	-	-	0.048514504	0.125588936	ABCG2,FLT1	0.00027022	0.020176545	EGLN3
amb2 Integrin signaling	0.0060547	0.05637933	ICAM1	-	-	-	-	-	-
Atypical NF-kappaB pathway	0.00104927	0.05637933	REL	-	-	-	-	-	-
Beta2 integrin cell surface interactions	0.00306427	0.05637933	ICAM1	-	-	-	-	-	-
CD40/CD40L signaling	0.00349676	0.05637933	TNFAIP3	-	-	-	-	-	-
Class I PI3K signaling events	0.00889792	0.056946673	FOXO3	-	-	-	-	-	-
Class I PI3K signaling events mediated by Akt	0.00444175	0.05637933	FOXO3	-	-	-	-	-	-
FoxO family signaling	0.00855758	0.05637933	FOXO3	-	-	-	-	-	-
IL2 signaling events mediated by PI3K	0.00444175	0.05637933	FOXO3	-	-	-	-	-	-
Insulin Pathway	0.0072568	0.05637933	FOXO3	-	-	-	-	-	-
mTOR signaling pathway	0.01691532	0.077327179	RRAGB	-	-	-	-	-	-
Notch-mediated HES/HEY network	0.00822326	0.05637933	NCOA1	-	-	-	-	-	-
p73 transcription factor network	0.02276095	0.096197206	FOXO3	-	-	-	-	-	-
Regulation of Androgen receptor activity	0.0004888	0.05637933	REL,NCOA1	-	-	-	-	-	-
Retinoic acid receptors-mediated signaling	0.00495364	0.05637933	NCOA1	-	-	-	-	-	-
RXR and RAR heterodimerization with other nuclear	0.00372306	0.05637933	NCOA1	-	-	-	-	-	-
Signaling events mediated by HDAC Class III	0.00265888	0.05637933	FOXO3	-	-	-	-	-	-
Signaling events mediated by Stem cell factor recep	0.00959652	0.059711701	FOXO3	-	-	-	-	-	-
Sphingosine 1-phosphate (S1P) pathway	0.00176595	0.05637933	SGPL1	-	-	-	-	-	-
Trk receptor signaling mediated by PI3K and PLC-ga	0.00495364	0.05637933	FOXO3	-	-	-	-	-	-
Validated transcriptional targets of AP1 family mem	0.00495364	0.05637933	MGP	-	-	-	-	-	-
a4b7 Integrin signaling	-	-	-	0.018814343	0.075257371	VCAM1	-	-	-
ALK1 pathway	-	-	-	0.001726884	0.020722563	FKBP1A	-	-	-
ALK2 signaling events	-	-	-	0.027840708	0.091710566	FKBP1A	-	-	-
Alpha-synuclein signaling	-	-	-	0.007973222	0.047000044	PARK7,FKBP1A,PTK2B	-	-	-
Alpha4 beta1 integrin signaling events	-	-	-	0.001107092	0.016532567	JAM2,VCAM1,CD81,PTK2B	-	-	-
Alpha9 beta1 integrin signaling events	-	-	-	0.021853348	0.081585833	VCAM1,TGM2	-	-	-
Arf6 downstream pathway	-	-	-	0.049882177	0.125588936	PLAUR	-	-	-
ATF-2 transcription factor network	-	-	-	0.000539334	0.014560972	JUND,ATF3,PDGFRA,CDK4,IL6,FOS	-	-	-
BARD1 signaling events	-	-	-	0.035312362	0.105466256	PCNA,TOPBP1	-	-	-
BCR signaling pathway	-	-	-	0.021784434	0.081585833	BCL2A1,PAG1,FOS,SHC1	-	-	-
Beta1 integrin cell surface interactions	-	-	-	0.001386041	0.01826313	JAM2,VCAM1,CD81,PLAUR,TGM2,ITGA8	-	-	-
Beta5 beta6 beta7 and beta8 integrin cell surface in	-	-	-	0.008783377	0.047175937	VCAM1,PLAUR	-	-	-
BMP receptor signaling	-	-	-	0.018400866	0.075257371	BMP6,CTDSPL,SMURF2	-	-	-
C-MYB transcription factor network	-	-	-	0.001055667	0.016532567	PRTN3,CSF1R,CEBPD,CDKN1A,ETS2,KITL	-	-	-

LUNG MET SPECIFIC PATHWAYS

PATHWAY	Mouse 1			Mouse 2			Mouse3		
	M1_HyperGeom_PVal	M1_BH	M1_Genes	M2_HyperGeom_PVal	M2_BH	M2_Genes	M3_HyperGeom_PVal	M3_BH	M3_Genes
Calcineurin-regulated NFAT-dependent transcriptio	-	-	-	0.030549671	0.096382061	CDK4,FOS,SLC3A2	-	-	-
Calcium signaling in the CD4+ TCR pathway	-	-	-	0.005652848	0.037242292	FKBP1A,FOS,RCAN1	-	-	-
Cellular roles of Anthrax toxin	-	-	-	0.015433689	0.066483585	VCAM1,IL1B	-	-	-
CXCR3-mediated signaling events	-	-	-	0.026697514	0.090609744	CXCL10,GNG2,ITGAL	-	-	-
CXCR4-mediated signaling events	-	-	-	0.003795429	0.032468243	RGS1,RHOB,PAG1,MMP9,GNG2,ITGA8,P	-	-	-
Direct p53 effectors	-	-	-	0.002098503	0.022384037	BCL2A1,ATF3,CCNG1,CTSD,CDKN1A,E2F	-	-	-
E2F transcription factor network	-	-	-	0.009056095	0.047175937	CES1,DSCR3,CDKN1A,E2F3,TOPBP1	-	-	-
EGF receptor (ErbB1) signaling pathway	-	-	-	0.013084653	0.061061714	SHC1,SHC1,SHC1	-	-	-
EGFR-dependent Endothelin signaling events	-	-	-	0.018814343	0.075257371	SHC1	-	-	-
Endogenous TLR signaling	-	-	-	0.000290686	0.013022711	BGN,LY96,S100A9,TLR6	-	-	-
FGF signaling pathway	-	-	-	0.003828132	0.032468243	PLAUR,MMP9,FOS,PTK2B,SHC1	-	-	-
FOXM1 transcription factor network	-	-	-	0.019910409	0.078244412	CDK4,CENPA,FOS	-	-	-
Glypican 1 network	-	-	-	0.029516771	0.095822559	FLT1,SMAD2	-	-	-
Hedgehog signaling events mediated by Gli proteins	-	-	-	0.030549671	0.096382061	SPOP,GNG2,SIN3B	-	-	-
HIV-1 Nef: Negative effector of Fas and TNF-alpha	-	-	-	0.01084374	0.053936085	MAP3K5,CYTIP,TNFRSF1A	-	-	-
IGF1 pathway	-	-	-	0.006368121	0.039623864	IGF1R,SHC1,SHC1	-	-	-
IL1-mediated signaling events	-	-	-	0.008866662	0.047175937	IL1B,IL1RN,MAP3K3	-	-	-
IL12-mediated signaling events	-	-	-	0.004146363	0.032468243	B2M,GADD45B,HLA-A,IL1B,FOS	-	-	-
IL2-mediated signaling events	-	-	-	0.011316946	0.053936085	MAPKAPK2,FOS,PTK2B,SHC1	-	-	-
IL27-mediated signaling events	-	-	-	0.024269712	0.086292311	IL1B,IL6	-	-	-
IL5-mediated signaling events	-	-	-	0.04392034	0.121458717	SDCBP	-	-	-
IL6-mediated signaling events	-	-	-	0.026697514	0.090609744	CEBPD,IL6,FOS	-	-	-
Integrin family cell surface interactions	-	-	-	0.032346411	0.100633278	ITGA8,ITGAL	-	-	-
Lissencephaly gene (LIS1) in neuronal migration and	-	-	-	0.035312362	0.105466256	LRPAP1,DYNLT1	-	-	-
LKB1 signaling events	-	-	-	0.026697514	0.090609744	CTSD,STK11,MAPT	-	-	-
LPA receptor mediated events	-	-	-	0.001271494	0.017800911	MMP9,HBEGF,GNG2,IL6,FOS,PTK2B	-	-	-
Nongenotropic Androgen signaling	-	-	-	0.041648624	0.118092302	GNG2,FOS	-	-	-
Osteopontin-mediated events	-	-	-	0.006368121	0.039623864	MMP9,FOS,PTK2B	-	-	-
p38 MAPK signaling pathway	-	-	-	0.000424395	0.014560972	MAP3K5,GADD45B,MAP3K3,TXN	-	-	-
p38 signaling mediated by MAPKAP kinases	-	-	-	0.013568784	0.062028727	LSP1,MAPKAPK2	-	-	-
PAR4-mediated thrombin signaling events	-	-	-	0.049882177	0.125588936	GNG2	-	-	-
PDGF receptor signaling network	-	-	-	0.008226034	0.047175937	PDGFRA	-	-	-
PDGFR-alpha signaling pathway	-	-	-	0.001757717	0.020722563	PDGFRA,FOS,SHC1	-	-	-
PDGFR-beta signaling pathway	-	-	-	0.039030678	0.11354379	ACTN4,JUND,PAG1,S1PR1,FOS,SHC1	-	-	-
Plasma membrane estrogen receptor signaling	-	-	-	0.000558662	0.014560972	IGF1R,MMP9,HBEGF,GNG2,SHC1	-	-	-
Plexin-D1 Signaling	-	-	-	0.00245746	0.025021406	ITGA8,NRP1,RRAS	-	-	-
PLK2 and PLK4 events	-	-	-	0.000585039	0.014560972	PLK2	-	-	-
Polo-like kinase signaling events in the cell cycle	-	-	-	0.003398323	0.031717678	PLK2	-	-	-
Rapid glucocorticoid signaling	-	-	-	0.014869658	0.065309868	GNG2	-	-	-
Ras signaling in the CD4+ TCR pathway	-	-	-	0.049882177	0.125588936	FOS	-	-	-
Regulation of cytoplasmic and nuclear SMAD2/3 sig	-	-	-	0.008783377	0.047175937	CTDSPL,SMAD2	-	-	-
Regulation of Ras family activation	-	-	-	0.000958223	0.016532567	RASGRP2,RASA2,GBP2,RRAS	-	-	-
Regulation of retinoblastoma protein	-	-	-	0.020529439	0.079286111	CEBPD,CDK4,CDKN1A,E2F3	-	-	-
Role of Calcineurin-dependent NFAT signaling in lym	-	-	-	0.011316946	0.053936085	FKBP8,FKBP1A,PRKCH,RCAN1	-	-	-
S1P3 pathway	-	-	-	0.035312362	0.105466256	S1PR1,FLT1	-	-	-

LUNG MET SPECIFIC PATHWAYS

PATHWAY	Mouse 1			Mouse 2			Mouse3		
	M1_HyperGeom_PVal	M1_BH	M1_Genes	M2_HyperGeom_PVal	M2_BH	M2_Genes	M3_HyperGeom_PVal	M3_BH	M3_Genes
SHP2 signaling	-	-	-	0.013987103	0.062662221	PAG1,IGF1R,IL6,SHC1	-	-	-
Signaling events mediated by HDAC Class II	-	-	-	0.048514504	0.125588936	GNG2,HDAC5	-	-	-
Signaling events mediated by PTP1B	-	-	-	0.036911283	0.108791151	CSF1R,SHC1,TXN	-	-	-
Signaling mediated by p38-gamma and p38-delta	-	-	-	0.027840708	0.091710566	MAPT	-	-	-
Syndecan-4-mediated signaling events	-	-	-	0.041648624	0.118092302	MMP9,SDCBP	-	-	-
TCR signaling in naïve CD8+ T cells	-	-	-	0.002080467	0.022384037	B2M,PAG1,RASGRP2,HLA-A,SHC1	-	-	-
TGF-beta receptor signaling	-	-	-	0.01051037	0.053507339	FKBP1A,SHC1,SMAD2,SMURF2	-	-	-
Validated targets of C-MYC transcriptional activatio	-	-	-	0.042723246	0.11962509	CDK4,MMP9,E2F3,LDHA	-	-	-
VEGF and VEGFR signaling network	-	-	-	0.007451802	0.045113609	NRP1,FLT1	-	-	-
VEGFR1 specific signals	-	-	-	0.004387242	0.032468243	CD2AP,NRP1,FLT1	-	-	-
Noncanonical Wnt signaling pathway	-	-	-	-	-	-	0.00023912	0.020176545	DAAM1
Regulation of nuclear beta catenin signaling and tar	-	-	-	-	-	-	0.0015673	0.026825267	COX2
Validated transcriptional targets of TAp63 isoforms	-	-	-	-	-	-	0.00076127	0.026825267	DHRS3

Supplementary Table 2. ICAM1 KO_vs_Control_Differential_Expr (related to Fig. 3)

Gene Symbol	Log2 Fold Change	LFC Standard Err	Wald Statistic	p Value	FDR Adj p Va	Significa	Status	Base Mean	KD	Control	siICAM1_1	siICAM1_2	siICAM1_3	siCon_1	siCon_2	siCon_3
ICAM1	-2.899577706	0.133762238	-21.67710225	3.37E-104	4.73E-100	Yes	OK	1368.747468	296.3880598	2441.106876	300.0653501	313.0570444	276.0417849	2934.716474	2235.475921	2153.128233
SEC23A	-2.217949205	0.111011262	-19.97949729	8.31E-89	5.82E-85	Yes	OK	2443.53063	829.1566509	4057.904608	847.2969916	857.5489996	782.6239615	4133.838952	4517.890969	3521.983904
GDAP1	2.726502298	0.137484526	19.83133946	1.60E-87	7.46E-84	Yes	OK	453.6597552	798.4053981	108.9141124	769.7725091	830.6860189	794.7576663	115.3163079	107.959992	103.4660371
IL24	2.407548636	0.121975577	19.73795655	1.02E-86	3.57E-83	Yes	OK	1502.139624	2554.655183	449.6240651	2838.308114	2391.838475	2433.81896	461.2652317	383.7273628	503.8796008
LPGAT1	-1.623400733	0.090765476	-17.88566315	1.53E-71	4.27E-68	Yes	OK	1994.992605	960.2421993	3029.743011	940.3263707	948.4698574	991.9303697	3173.705344	2941.909782	2973.613907
HIF1A	-1.651305298	0.095445807	-17.30097276	4.63E-67	1.08E-63	Yes	OK	6639.442794	3140.845578	10138.04001	3307.10322	3034.483629	3080.949884	11166.46248	9296.294093	9951.363452
GOLGA7B	3.451055543	0.199722725	17.27923318	6.74E-67	1.35E-63	Yes	OK	248.7008034	466.3457602	31.05584661	508.0133739	483.5336528	407.4902538	25.06876259	24.64304165	43.4557356
LSM14A	-1.641524477	0.095423089	-17.20259205	2.54E-66	4.45E-63	Yes	OK	1415.188413	672.7988045	2157.578021	678.5672355	666.4085599	673.420618	2052.296031	2189.710272	2230.727761
SCG2	3.022027272	0.177756184	17.0009684	8.08E-65	1.26E-61	Yes	OK	1102.706308	2004.513526	200.8990895	2324.822424	1756.425662	1932.292494	271.5782614	131.4295555	199.6894517
YOD1	1.887187863	0.111863692	16.87042356	7.43E-64	1.04E-60	Yes	OK	2521.531268	4005.861941	1037.200595	4099.677048	4437.557775	3480.351002	1011.106758	1120.671656	979.8233717
ENTPD7	1.932312834	0.115034794	16.79763805	2.54E-63	3.24E-60	Yes	OK	1122.791517	1796.576732	449.0063017	1960.913382	1849.412903	1579.403912	442.8814725	444.7482279	459.3892049
CDK6	-1.688314736	0.101865913	-16.57389298	1.08E-61	1.26E-58	Yes	OK	3591.984734	1660.505626	5523.463842	1618.893606	1732.662256	1629.961015	5989.763009	4855.852683	5724.775835
GHITM	-1.554850166	0.094236848	-16.49938637	3.71E-61	4.00E-58	Yes	OK	4461.015413	2223.575981	6698.454846	2337.591162	2292.652084	2040.484695	7077.747305	6756.887325	6260.729907
G3BP2	-1.44902114	0.090259404	-16.05396304	5.36E-58	5.37E-55	Yes	OK	4494.474288	2372.526068	6616.422508	2440.653121	2292.652084	2384.272999	7149.611091	6509.28343	6190.373002
PPM1F	-2.014868596	0.127498237	-15.80310941	2.96E-56	2.77E-53	Yes	OK	1597.380043	602.8963418	2591.863745	597.394542	518.662166	692.6323173	2571.219417	2291.802873	2912.568945
PPFIA4	-3.746750364	0.238743083	-15.69364994	1.67E-55	1.38E-52	Yes	OK	140.9394456	10.70424486	271.1746464	12.7687383	6.199149395	13.1448469	291.6332715	292.1960653	229.6946024
HMGA2	1.698586704	0.108418114	15.66700107	2.54E-55	1.98E-52	Yes	OK	2719.394406	4193.250983	1245.53783	4314.921493	3968.488804	4296.342651	1148.149327	1152.355567	1436.108595
CNIH4	-1.69853302	0.115526314	-14.70256395	6.21E-49	4.58E-46	Yes	OK	1095.912836	500.0191112	1691.806562	499.8048992	532.0936564	468.158778	1868.458439	1683.941179	1523.020067
RCSD1	-2.544120902	0.173198813	-14.68902043	7.58E-49	5.31E-46	Yes	OK	269.7716153	68.66606135	470.8771692	80.26064075	64.05787708	61.67966621	498.0327502	506.9425711	407.6561863
LOC105376575	-1.530167506	0.10466617	-14.61950411	2.11E-48	1.35E-45	Yes	OK	1419.093712	713.549572	2124.637851	701.3685539	777.993249	661.2869132	2193.516727	2189.710272	1990.686554
NUCB2	-1.530167506	0.10466617	-14.61950411	2.11E-48	1.35E-45	Yes	OK	1419.093712	713.549572	2124.637851	701.3685539	777.993249	661.2869132	2193.516727	2189.710272	1990.686554
SLCO4A1	-2.087297996	0.142777273	-14.61925944	2.12E-48	1.35E-45	Yes	OK	686.6357753	244.7998701	1128.47168	204.2998128	296.5259794	233.573818	1093.833674	1240.36643	1051.214937
NUDT15	-1.87582143	0.131536123	-14.26088429	3.84E-46	2.34E-43	Yes	OK	566.1437508	231.1651922	901.1223093	225.2770258	239.7004433	228.5181076	1016.956136	821.4347217	864.9760704
YWHAZ	-1.297422352	0.092272223	-14.06081163	6.61E-45	3.86E-42	Yes	OK	7148.702707	4076.745517	10220.6599	4050.4262	4343.537343	3836.27301	11048.6393	9623.694503	9989.645885
TRIM16L	-2.300034993	0.164384561	-13.99179451	1.75E-44	9.81E-42	Yes	OK	609.782051	185.3545436	1034.209558	160.5212815	150.8459686	244.6963807	1048.709902	910.6190629	1143.29971
FAM46C	-1.913337713	0.139164239	-13.74877432	5.18E-43	2.79E-40	Yes	OK	977.9930193	387.7697981	1568.21624	394.0067819	323.3889601	445.9136525	1667.908338	1329.550771	1707.189613
MAT2B	-1.748625604	0.127746231	-13.68827554	1.19E-42	6.19E-40	Yes	OK	925.3379946	407.1667329	1443.509256	403.1273092	443.2391817	375.1337076	1573.482665	1527.868582	1229.176521
TRIM16	-1.890837768	0.138523331	-13.64995889	2.02E-42	1.00E-39	Yes	OK	751.4711661	302.1699806	1200.772352	270.8796625	266.563424	369.0668552	1194.94435	1117.151221	1290.221483
REST	-1.400806533	0.102637103	-13.6481496	2.07E-42	1.00E-39	Yes	OK	1241.397676	669.0654191	1813.729933	643.9092315	723.234096	640.0529297	1891.85595	1735.574219	1813.759631
CCNA1	-2.996963718	0.223380561	-13.41640341	4.85E-41	2.26E-38	Yes	OK	130.9093745	20.60659554	241.2121535	20.97721292	18.59794818	22.24512552	225.6188633	282.8082399	215.2093572
MIR210HG	-3.30872157	0.247459808	-13.37074331	8.96E-41	4.05E-38	Yes	OK	113.3925994	11.92826526	214.8569336	7.296421887	8.265532526	20.22284138	228.961365	215.919984	199.6894517
YKT6	-1.173964728	0.088486997	-13.26708747	3.59E-40	1.57E-37	Yes	OK	1977.813077	1201.178912	2754.447242	1161.043133	1211.933707	1230.559898	2834.441424	2676.703714	2752.196588
SEL1L	-1.284034951	0.097241995	-13.20453111	8.26E-40	3.51E-37	Yes	OK	2213.504133	1268.935958	3158.072308	1278.697936	1196.435833	1331.674105	3400.995458	2963.032389	3110.189076
CPNE2	-1.921991231	0.145793039	-13.18301097	1.10E-39	4.53E-37	Yes	OK	362.2356233	141.9169258	582.5543207	139.5440686	144.6468192	141.5598897	590.7871718	639.5456047	517.3301857
MCM3	-1.281594276	0.097798812	-13.10439511	3.11E-39	1.24E-36	Yes	OK	7316.832865	4198.003661	10435.66207	3940.979872	4438.590967	4214.440144	10152.84885	11566.97436	9587.163001
HIST2H2AB	-1.488563709	0.114095325	-13.04666702	6.64E-39	2.58E-36	Yes	OK	737.6078545	377.6569924	1097.558717	376.6777799	377.1149215	379.1782759	1180.738718	1016.232099	1095.705333
TMEM198	2.63724413	0.203091904	12.98547151	1.48E-38	5.60E-36	Yes	OK	176.7337448	314.0163973	39.45109233	321.9546158	344.0527914	276.0417849	32.58939137	34.03086704	51.73301857
CYR61	1.410744624	0.109451464	12.88922574	5.18E-38	1.91E-35	Yes	OK	6047.407313	8864.352049	3230.462578	8514.012289	9657.241565	8421.802293	3522.161144	2760.020665	3409.205924
MARCH4	2.44779723	0.1915147	12.78124985	2.09E-37	7.50E-35	Yes	OK	301.1646908	522.8136444	79.51573708	473.3553699	616.8153648	478.2701987	53.48002686	95.05173208	90.01545231
STX3	1.221031633	0.096353317	12.67244005	8.41E-37	2.94E-34	Yes	OK	4979.284403	7012.057323	2946.511483	7155.965765	7592.924817	6287.281385	3047.525906	2973.593692	2818.414852
LINC00704	-1.552906707	0.122822692	-12.64348372	1.22E-36	4.15E-34	Yes	OK	557.8862159	274.5109149	841.261517	267.2314516	292.3932131	263.90808	870.7216874	871.8942831	781.1685804
STEAP3	-1.322130832	0.1052462	-12.56226671	3.40E-36	1.14E-33	Yes	OK	1161.58254	650.852303	1672.312778	633.8766514	623.0145142	695.6657435	1591.030799	1794.248128	1631.659406
BLOC1S6	-1.571262557	0.126092209	-12.46121844	1.21E-35	3.96E-33	Yes	OK	676.7551648	329.2677402	1024.242589	315.5702466	323.3889601	348.8440138	1153.163079	929.3947137	990.1699754
ITPA	-1.709547113	0.138669992	-12.32816913	6.39E-35	2.03E-32	Yes	OK	530.7510284	236.7735916	824.7284652	222.5408675	208.7046963	279.0752111	848.9954265	863.6799359	761.5100333
SH3PXD2A	-1.454359707	0.118116016	-12.31297628	7.71E-35	2.40E-32	Yes	OK	1108.37515	576.4617789	1640.288521	523.5182704	548.6247214	657.2423449	1532.53702	1625.267271	1763.061273
ASNSD1	-1.295627756	0.105767868	-12.24972932	1.69E-34	5.13E-32	Yes	OK	1346.065395	764.7444057	1927.386385	762.4760872	799.6902719	732.066858	2056.474158	1984.351592	1741.333405
HSP90AB1	-0.941655441	0.07714066	-12.20699225	2.85E-34	8.50E-32	Yes	OK	25442.19562	17307.64696	33576.74428	17866.20104	17362.78426	16693.95556	34252.28596	32238.96587	34238.98101
CHST15	-1.072107102	0.088019936	-12.1802758	3.96E-34	1.16E-31	Yes	OK	2577.931464	1645.062934	3510.799995	1683.64935	1549.787349	1701.752102	3617.422442	3466.454525	3448.523018
C6orf120	-1.347082703	0.110700786	-12.16868239	4.56E-34	1.31E-31	Yes	OK	800.2722837	441.780431	1158.764136	449.6419988	428.7744998	446.9247945	1202.464979	1203.988606	1069.838824
UBA2	-1.146757182	0.09490621	-12.08305738	1.30E-33	3.64E-31	Yes	OK	2008.93732	1234.174679	2783.699962	1231.271193	1327.651162	1143.60168	2860.345812	2744.765449	2745.988626
AVL9	1.070178848	0.088834759	12.04684811	2.02E-33	5.54E-31	Yes	OK	3262.541374	4441.893714	2083.189034	4591.273472	4129.666688	4604.740982	2135.858573	2019.555937	2094.152592

KO_vs_Control_Differential_Expr

Gene Symbol	Log2 Fold Change	LFC Standard Err	Wald Statistic	p Value	FDR Adj p Va	Significa	Status	Base Mean	KD	Control	siICAM1_1	siICAM1_2	siICAM1_3	siCon_1	siCon_2	siCon_3
LRRFIP1	-1.247225211	0.104096035	-11.98148621	4.44E-33	1.20E-30	Yes	OK	1418.600635	826.4596139	2010.741656	748.7952961	877.1796393	853.4039063	1916.924713	2120.47506	1994.825196
ANKRD1	1.24837005	0.104503606	11.94571265	6.84E-33	1.81E-30	Yes	OK	10025.64355	14215.81921	5835.467886	14307.37127	15910.11692	12429.96945	6174.436227	5469.581768	5862.385664
PFKFB4	-1.661023036	0.139123289	-11.9392163	7.39E-33	1.92E-30	Yes	OK	690.6383533	316.6859335	1064.590773	267.2314516	298.5923625	384.2339862	1071.271788	1123.018612	999.4819187
MRC2	-1.724401078	0.14564411	-11.83982704	2.43E-32	6.19E-30	Yes	OK	560.5457721	246.4145564	874.6769877	242.6060277	197.3395891	299.2980524	869.886062	912.9660192	841.1788819
TCF12	-1.210026961	0.102235737	-11.83565553	2.55E-32	6.39E-30	Yes	OK	1197.865953	711.6445361	1684.08737	725.9939777	682.939625	726.0000056	1804.950907	1587.715969	1659.595236
FAM234B	1.627421554	0.139930253	11.63023376	2.89E-31	7.11E-29	Yes	OK	458.2434475	702.3419904	214.1449046	659.414128	789.3583562	658.253487	205.5638533	238.2160693	198.6547913
RFX5	-1.067904777	0.091909716	-11.61906292	3.30E-31	7.97E-29	Yes	OK	1950.323076	1245.892902	2654.75325	1258.632775	1171.639236	1307.406695	2726.645745	2660.27502	2577.338985
STT3A	-1.054515954	0.091551793	-11.51824471	1.07E-30	2.54E-28	Yes	OK	6675.380742	4292.507201	9058.254283	4463.586089	4037.712639	4376.222875	9750.913023	8389.195465	9034.654363
MAGEF1	-1.522203542	0.132227577	-11.51199754	1.15E-30	2.68E-28	Yes	OK	576.2808531	286.6782498	865.8834565	257.1988715	293.4264047	309.4094731	863.2010586	956.3847117	778.0645993
NUDT3	-1.198840452	0.104371696	-11.48626015	1.55E-30	3.55E-28	Yes	OK	2313.939707	1380.948656	3246.930759	1281.434094	1510.526069	1350.885804	3053.375284	3554.465388	3132.951604
ETS1	1.089290399	0.094944464	11.47292169	1.80E-30	4.08E-28	Yes	OK	7964.316079	10897.06542	5031.566737	11227.36918	11764.95236	9698.874726	4875.038699	5031.874409	5187.787102
ICK	-1.524169507	0.13322998	-11.44013914	2.63E-30	5.86E-28	Yes	OK	1374.637048	682.4695777	2066.804518	622.0199658	790.3915478	634.9972194	1916.924713	2431.446776	1852.042065
S100A2	-1.800813067	0.158023297	-11.39587077	4.38E-30	9.60E-28	Yes	OK	1200.323669	499.3024581	1901.34488	498.8928465	426.7081167	572.3064111	2264.544888	1410.520765	2028.968988
PVR	0.970198324	0.085212029	11.38569677	4.93E-30	1.06E-27	Yes	OK	4378.760489	5822.99942	2934.521557	6013.163687	5856.129795	5599.704778	2949.757732	2778.796316	3075.010624
OLFML2A	-1.726115555	0.151893504	-11.36398535	6.32E-30	1.34E-27	Yes	OK	772.9284258	337.6214484	1208.235403	293.6809809	296.5259794	422.6573849	1036.175521	1336.59164	1251.939049
SNTB2	-1.143047277	0.101597741	-11.25071551	2.30E-29	4.80E-27	Yes	OK	1321.8032	812.0734458	1831.532953	765.2122454	786.2587816	884.7493104	1891.020325	1774.298999	1829.279537
CPA4	1.685748698	0.15092062	11.16977059	5.73E-29	1.18E-26	Yes	OK	1712.770261	2659.728906	765.8116161	3061.761034	2332.946556	2584.479129	869.0504365	564.4430016	863.9414101
DDIT4	-1.903797344	0.170773688	-11.14807188	7.32E-29	1.49E-26	Yes	OK	2049.524678	789.754784	3309.294572	753.3555598	573.421319	1042.487473	3870.616944	2653.234151	3404.032622
HIST1H2BN	-1.241394766	0.111800543	-11.10365595	1.20E-28	2.41E-26	Yes	OK	1041.697814	606.7352193	1476.660409	589.1860674	637.4791961	593.5403945	1432.261969	1638.175531	1359.543728
TMCO3	-1.325596464	0.11984159	-11.06123893	1.93E-28	3.82E-26	Yes	OK	759.5613853	422.6178995	1096.504871	382.1500963	471.135354	414.5682483	1144.806825	1118.3247	1026.383088
TMEM144	1.624602898	0.147084854	11.04534463	2.31E-28	4.49E-26	Yes	OK	357.7968801	549.5804304	166.0133298	598.3065947	514.5293998	535.9052966	173.8100873	143.1643372	181.065565
CANX	-0.898585286	0.08165505	-11.00465047	3.63E-28	6.97E-26	Yes	OK	27531.55847	19090.99379	35972.12315	19017.2116	19009.69162	19246.07814	38492.24934	33756.27315	35667.84698
FNIP2	1.154758745	0.105462533	10.94946906	6.68E-28	1.27E-25	Yes	OK	1127.701734	1567.983419	687.4200493	1585.147655	1678.936294	1439.866306	711.9528576	665.3621245	684.9451658
RAB23	-1.431062541	0.130770171	-10.94334071	7.15E-28	1.34E-25	Yes	OK	599.9944734	314.0266595	885.9622873	274.5278735	340.9532167	326.5988883	933.3935939	924.700801	799.792467
ANKRD13B	1.877675907	0.172459746	10.88761841	1.32E-27	2.43E-25	Yes	OK	269.1793014	433.1425693	105.2160336	436.8732605	428.7744998	433.7799476	88.57629449	91.53129756	135.5405086
LDHA	-1.170213536	0.107513329	-10.88435773	1.37E-27	2.49E-25	Yes	OK	16031.71491	9695.707069	22367.72275	10247.82454	9746.09604	9093.200627	25776.53732	21596.69231	19729.93862
XPOT	-0.87296396	0.080248094	-10.87831389	1.46E-27	2.63E-25	Yes	OK	3636.173011	2551.803033	4720.54299	2543.71508	2623.273385	2488.420632	4740.503006	4771.362255	4649.763709
C5orf15	-1.492189849	0.137191503	-10.87669289	1.49E-27	2.64E-25	Yes	OK	1200.454435	605.0726546	1795.836215	601.9548057	705.6698394	507.5933187	1941.15785	1923.330727	1523.020067
TMEM30A	-1.211844867	0.111593935	-10.8594151	1.80E-27	3.15E-25	Yes	OK	4234.517454	2504.504512	5964.530395	2478.959336	2846.442764	2188.111437	6336.547558	6130.24998	5426.793648
LBH	-2.501389635	0.231018611	-10.82765421	2.55E-27	4.40E-25	Yes	OK	94.41506764	21.00970245	167.8204328	19.15310745	18.59744818	25.27855173	161.275706	172.5012915	169.6843009
P3H2	1.19421742	0.110309649	10.82604677	2.59E-27	4.43E-25	Yes	OK	1508.709761	2116.250185	901.1693366	2223.58457	2213.096334	1912.069653	872.3929382	995.1094914	836.00558
SERPINE1	1.502424263	0.138799385	10.82443023	2.64E-27	4.45E-25	Yes	OK	24740.08147	37095.53281	12384.63012	36217.61414	44242.29604	30826.68826	14175.54962	10010.9423	12967.39843
DNAH11	-2.148968684	0.198951777	-10.80145509	3.39E-27	5.65E-25	Yes	OK	167.715964	52.69442831	282.7374996	54.72316415	57.85872768	45.50139311	295.8113986	322.7064978	229.6946024
DISP2	1.628452373	0.151236905	10.76755948	4.90E-27	8.07E-25	Yes	OK	326.5641047	501.8539902	151.2742192	446.9058406	520.7285491	537.9275807	135.371318	164.2869443	154.1643953
PRKAA1	-1.044345104	0.097223289	-10.74171745	6.48E-27	1.06E-24	Yes	OK	2054.503045	1325.932999	2783.073092	1349.838049	1374.144782	1253.816166	3012.429638	2641.499369	2695.290267
RAB39A	3.028015019	0.281951815	10.73947693	6.64E-27	1.07E-24	Yes	OK	127.9786578	243.6365314	12.32078417	152.3128069	389.5132203	189.0835669	5.013752519	16.42869443	15.51990557
NECAP1	1.177492466	0.110417507	10.66400153	1.50E-26	2.39E-24	Yes	OK	1140.473812	1594.829181	686.1184437	1565.994547	1754.359279	1464.133716	728.665366	674.7499499	654.9400151
YWHAB	-0.936993389	0.087967738	-10.65155717	1.71E-26	2.70E-24	Yes	OK	6965.99007	4739.626654	9192.353486	4899.547297	4815.705888	4503.626776	9876.256836	8683.738486	9017.065136
SPOPL	-1.397620589	0.131431132	-10.63386252	2.07E-26	3.23E-24	Yes	OK	692.1455473	368.5555733	1015.735521	342.9318287	394.6791781	368.0557131	1113.053059	1070.212095	863.9414101
CD82	-1.334363333	0.125964802	-10.59314437	3.21E-26	4.94E-24	Yes	OK	1746.929646	964.0346145	2529.824678	894.7237339	1012.527734	984.8523753	2547.821905	2945.430216	2096.221912
ARPC1A	-1.014587623	0.095797188	-10.59099589	3.28E-26	4.96E-24	Yes	OK	2293.139858	1501.331847	3084.947868	1495.766487	1567.351605	1440.877448	3140.280327	3277.524539	2837.038738
ZBED6CL	-1.429942012	0.135019149	-10.59066084	3.29E-26	4.96E-24	Yes	OK	543.4408104	283.6308131	803.2508076	249.9024496	301.6919372	299.2980524	710.2816068	853.1186324	846.3521838
MAPK11	1.768778498	0.167156011	10.5816027	3.63E-26	5.41E-24	Yes	OK	245.0166999	387.4574681	102.5759317	362.9969889	358.5174733	440.8579421	104.4531775	91.53129756	111.7433201
PURB	-1.171027099	0.110676412	-10.58063845	3.66E-26	5.41E-24	Yes	OK	1226.820058	740.5643113	1713.075804	776.1568782	644.711537	800.8245187	1701.333355	1773.125521	1664.768537
MIR4657	-1.171027099	0.110676412	-10.58063845	3.66E-26	5.41E-24	Yes	OK	1226.820058	740.5643113	1713.075804	776.1568782	644.711537	800.8245187	1701.333355	1773.125521	1664.768537
AXL	-0.988231436	0.09349292	-10.57011963	4.10E-26	5.98E-24	Yes	OK	18706.89481	12408.73336	25005.05625	12208.73792	12071.81025	12945.65191	22332.92497	27047.49843	25634.74536
ICOSLG	-2.186478088	0.207835037	-10.5202574	6.97E-26	1.01E-23	Yes	OK	126.0868964	37.73627836	214.4375145	44.69058406	36.1617048	32.35654621	211.4132312	231.1752002	200.724112
LOC102723996	-2.186478088	0.207835037	-10.5202574	6.97E-26	1.01E-23	Yes	OK	126.0868964	37.73627836	214.4375145	44.69058406	36.1617048	32.35654621	211.4132312	231.1752002	200.724112
ATAD2	-0.903781431	0.086853739	-10.40578616	2.33E-25	3.34E-23	Yes	OK	6404.218888	4426.006191	8382.431585	4579.416787	4489.217353	4209.384433	8941.191991	8267.153734	7938.949029
NNT	-0.900750723	0.086574214	-10.40437662	2.37E-25	3.35E-23	Yes	OK	3430.700722	2374.414086	4486.987358	2422.412066	2465.195076	2235.635115	4631.036076	4496.768362	4333.157635
PARD6B	1.008570757	0.097260136	10.36982672	3.40E-25	4.77E-23	Yes	OK	3108.49069	4176.5298	2040.45158	4249.253696	3747.385809	4532.949896	2005.501007	2028.943763	2086.909969
SEMA7A	1.591260839	0.153895425	10.33988396	4.65E-25	6.45E-23	Yes	OK	3218.845769	4921.748914	1515.942625	5549.840898	3831.074326	5384.331518	1574.318291	1171.131217	1802.378367

KO_vs_Control_Differential_Expr

Gene Symbol	Log2 Fold Change	LFC Standard Err	Wald Statistic	p Value	FDR Adj p Va	Significa	Status	Base Mean	KD	Control	siICAM1_1	siICAM1_2	siICAM1_3	siCon_1	siCon_2	siCon_3
ATP6V0A1	1.236467966	0.119746349	10.32572578	5.39E-25	7.41E-23	Yes	OK	1186.222892	1681.651513	690.7942715	1684.561403	1580.783096	1779.610042	624.2121886	644.2395174	803.9311085
MIR5010	1.236467966	0.119746349	10.32572578	5.39E-25	7.41E-23	Yes	OK	1186.222892	1681.651513	690.7942715	1684.561403	1580.783096	1779.610042	624.2121886	644.2395174	803.9311085
DPYSL2	-0.850038985	0.082931745	-10.24986255	1.19E-24	1.58E-22	Yes	OK	4760.86747	3375.145231	6146.58971	3260.588531	3420.897274	3443.949887	6346.575063	5834.53348	6258.660586
P4HA1	-1.264874803	0.123405002	-10.24978556	1.19E-24	1.58E-22	Yes	OK	2290.150503	1312.174581	3268.126424	1384.496053	1227.43158	1324.59611	3924.096971	2950.124129	2930.158172
PFKFB2	0.928179553	0.09055652	10.2497264	1.19E-24	1.58E-22	Yes	OK	1662.398154	2189.552899	1135.243409	2248.209994	2219.295483	2101.15322	1113.053059	1135.926872	1156.750295
USP22	-0.869353695	0.084872558	-10.24304817	1.27E-24	1.68E-22	Yes	OK	5504.048526	3866.0043	7142.092753	3804.171961	3693.659848	4100.18109	7102.816068	6897.704706	7425.757485
S100A4	-1.280105352	0.125017147	-10.23943824	1.32E-24	1.73E-22	Yes	OK	5472.62246	3108.034491	7837.210428	2788.145213	3360.972163	3174.986097	7739.562638	9259.91627	6512.152377
LRIG1	-1.264648058	0.12377165	-10.21759066	1.65E-24	2.15E-22	Yes	OK	761.2116162	436.2921255	1086.131107	449.6419988	415.3430094	443.8913683	1221.684364	957.5581898	1079.150767
ATP11C	-1.451784563	0.142151443	-10.21294282	1.74E-24	2.23E-22	Yes	OK	1101.672289	565.7034752	1637.641102	649.3815479	482.5004612	565.2284166	1809.964659	1329.550771	1773.407876
EPG5	1.077545183	0.105554612	10.20841403	1.82E-24	2.32E-22	Yes	OK	6586.083911	9000.161179	4172.006644	8410.038277	10404.23907	8186.206191	4071.167045	4346.563156	4098.289731
SCAMP1	-1.477279308	0.145020231	-10.18671184	2.27E-24	2.87E-22	Yes	OK	767.6022579	387.7370575	1147.467458	422.2804167	415.3430094	325.5877462	1161.519333	1324.856858	956.0261831
GFPT2	-1.091154531	0.107696393	-10.13176483	3.99E-24	5.00E-22	Yes	OK	1029.231625	647.0889181	1411.374332	608.3391748	674.6740924	658.253487	1335.329421	1527.868582	1370.924992
PRNP	-1.118031602	0.110402942	-10.12682793	4.20E-24	5.21E-22	Yes	OK	13349.48093	8277.181541	18421.78032	7621.112661	9371.047502	7839.384461	19363.94785	19773.10723	16128.28587
LRRC8A	-0.893727373	0.088533248	-10.09482196	5.82E-24	7.16E-22	Yes	OK	2003.41678	1390.769053	2616.064506	1341.629574	1444.401809	1386.275777	2628.877571	2540.580246	2678.735701
ZNF268	-1.208447274	0.120291773	-10.04596774	9.57E-24	1.17E-21	Yes	OK	649.5606873	383.5687425	915.552632	404.039362	399.845136	346.8217297	979.352992	893.0168903	874.2880138
CAPG	-1.043591448	0.10409464	-10.02541006	1.18E-23	1.42E-21	Yes	OK	4810.029265	3098.661288	6521.397241	2927.689282	3180.163639	3188.130944	6367.465699	7378.830757	5817.895268
UBE2Q2	-1.205629296	0.120333637	-10.01905476	1.26E-23	1.51E-21	Yes	OK	766.7705362	453.5733025	1079.96777	468.7830535	478.367695	413.5571062	1175.724966	979.8542751	1084.324069
MMP24	2.013087141	0.201991652	9.966189804	2.14E-23	2.54E-21	Yes	OK	295.1174768	489.2272842	101.0076693	467.8830535	652.9770696	346.8217297	101.1106758	115.000861	86.91147119
GPR68	-1.629036603	0.163626847	-9.955802698	2.38E-23	2.80E-21	Yes	OK	912.4862932	416.7978422	1408.174744	467.8830535	278.9617228	503.5487504	1448.138852	1376.489898	1399.895482
TWSG1	-1.051043374	0.105644935	-9.948828857	2.55E-23	2.98E-21	Yes	OK	1836.349296	1178.519818	2494.178775	1219.414508	1214.00009	1102.144855	2768.427016	2424.405907	2289.703402
SURF4	-0.921548223	0.092857495	-9.92432781	3.26E-23	3.78E-21	Yes	OK	7121.396575	4875.567904	9367.225247	4885.866506	4666.926303	5073.910903	10203.822	8565.217191	9332.63655
SGCE	-1.296352647	0.131727184	-9.841193058	7.48E-23	8.59E-21	Yes	OK	536.5027313	301.0411145	771.9643482	342.9318287	285.1608722	275.0306428	830.6116672	739.2912495	745.9901277
SMAD2	-0.984231279	0.100194737	-9.823183453	8.95E-23	1.02E-20	Yes	OK	2114.187664	1402.877505	2825.497823	1521.303963	1313.18648	1374.142072	3026.63527	2661.448498	2788.409701
NSMAF	-1.125483315	0.114809083	-9.803086026	1.09E-22	1.23E-20	Yes	OK	981.9142856	605.7587366	1358.069835	589.1860674	547.5915299	680.4986125	1426.412592	1356.540769	1291.256143
SYP	2.392252112	0.24457471	9.781273446	1.35E-22	1.52E-20	Yes	OK	158.7655231	281.1245006	36.40654568	239.8698695	419.4757757	184.0278566	36.76751847	35.20434521	37.24777337
GPR157	1.164342585	0.119472457	9.745698822	1.92E-22	2.14E-20	Yes	OK	865.740573	1209.039551	522.4415955	1254.072512	1084.851144	1288.194996	542.3208974	469.3912695	555.6126194
ITM2B	-0.903802815	0.093075227	-9.710455074	2.72E-22	3.00E-20	Yes	OK	1583.023251	1092.721561	2073.32494	1128.209234	1054.888589	1095.066861	2171.790466	2025.423328	2022.761026
SEPT11	-0.782825885	0.080720716	-9.697955163	3.08E-22	3.37E-20	Yes	OK	5096.019684	3724.651804	6467.387564	3910.882131	3602.73899	3660.33429	6563.837672	6448.262565	6390.062453
TMEM65	-1.508705261	0.155715338	-9.68886738	3.36E-22	3.65E-20	Yes	OK	381.0951981	187.8843285	574.3060677	199.7395491	184.9412903	178.9721462	628.3903157	462.3504005	632.1774869
DNMT1	-0.871064672	0.090031801	-9.675077673	3.85E-22	4.13E-20	Yes	OK	5901.676687	4137.750082	7665.603292	3976.549928	4251.583293	4185.117024	7014.239773	7829.446376	8153.123726
B3GALNT1	-1.214802923	0.12564791	-9.668309795	4.11E-22	4.36E-20	Yes	OK	566.0087489	332.5679235	799.4495743	327.4269322	326.4885348	343.7883035	889.1054466	775.6690729	733.5742033
FAM102B	-1.098945312	0.113862803	-9.651486575	4.84E-22	5.10E-20	Yes	OK	1340.571404	838.6560147	1842.486794	753.3555598	909.2085779	853.4039063	2011.350385	1673.379876	1842.730121
RHOB	1.271691467	0.131843387	9.645470241	5.14E-22	5.37E-20	Yes	OK	680.3587279	973.9233328	386.7941231	924.8214741	1134.444339	862.5041849	389.4014456	403.6764918	367.3044318
SLC36A1	1.460053821	0.152539838	9.571622989	1.05E-21	1.09E-19	Yes	OK	922.27334	1375.764784	468.7818963	1237.655563	1553.920115	1335.718673	349.2914255	530.4121346	526.642129
PHACTR2	0.885402478	0.092514322	9.57043683	1.06E-21	1.10E-19	Yes	OK	2295.077362	2992.62331	1597.531415	3080.002089	3121.27172	2776.596122	1636.990197	1538.429886	1617.17416
EIF1B	-1.600370538	0.167425168	-9.558721428	1.19E-21	1.22E-19	Yes	OK	242.1206599	111.9390639	372.3022559	116.7427502	126.049371	93.02507035	357.6476797	409.5438827	349.7152055
SLC1A4	-1.697290898	0.177732034	-9.549718539	1.30E-21	1.32E-19	Yes	OK	200.7165597	86.89610685	314.5370126	82.08474623	77.48936743	101.1142069	350.1270509	285.1551962	308.3287907
FZD1	-1.180176542	0.123593837	-9.548830062	1.31E-21	1.32E-19	Yes	OK	792.7360584	474.0238774	1111.448239	445.0817351	437.0400323	539.9498649	1018.627387	1138.273829	1177.443503
CUL5	-0.917802764	0.096653502	-9.495804553	2.19E-21	2.19E-19	Yes	OK	1758.073572	1204.761026	2311.386118	1247.688143	1256.360944	1110.233992	2389.888701	2213.179836	2331.089817
ADRB2	1.027610022	0.108545202	9.467116061	2.88E-21	2.86E-19	Yes	OK	1596.862077	2158.325348	1035.398806	2030.22939	2452.796777	1991.949876	1021.134263	1051.436444	1033.625711
ZBTB47	1.038372156	0.109982471	9.441251393	3.68E-21	3.64E-19	Yes	OK	1302.55277	1764.814726	840.2908131	1625.277975	1930.001845	1739.164359	778.8028912	907.0986284	834.9709197
KRTAP2-3	2.819865504	0.298973438	9.431826199	4.03E-21	3.95E-19	Yes	OK	61.40909804	118.0962708	4.721925316	96.67759	178.7421409	78.86908139	2.506876259	2.346956348	9.311943342
FAM171B	-1.339719652	0.142174029	-9.423096872	4.38E-21	4.26E-19	Yes	OK	488.6358133	266.5074641	710.7641624	280.0001899	227.3021445	292.220058	782.9810183	718.1686424	631.1428265
MAPK6	0.948095232	0.10090205	9.39619397	5.66E-21	5.47E-19	Yes	OK	2502.97249	3317.633284	1688.311697	3479.481187	3320.677692	3152.740971	1858.430934	1531.389017	1675.115141
HIST1H2BC	-0.758711028	0.080764884	-9.394070657	5.77E-21	5.54E-19	Yes	OK	4520.894241	3340.485973	5701.302509	3258.764425	3324.810459	3437.883035	5914.556721	5602.184802	5587.166005
WDR17	1.773638259	0.188912627	9.38866973	6.08E-21	5.79E-19	Yes	OK	147.5026353	235.2421011	59.76317152	231.6613949	229.3685276	244.6963807	63.5075319	51.63303965	64.14894302
COL4A1	1.030225762	0.109960303	9.369069899	7.32E-21	6.93E-19	Yes	OK	2491.14733	3369.598647	1612.696013	3293.422429	3850.704966	2964.668546	1545.907027	1634.655096	1657.525915
MIR4523	-0.743648809	0.079432709	-9.361997289	7.82E-21	7.36E-19	Yes	OK	3871.919681	2880.340405	4863.498957	2910.36028	2867.106595	2863.55434	4936.039355	4933.302243	4721.155274
TAOK1	-0.743648809	0.079432709	-9.361997289	7.82E-21	7.36E-19	Yes	OK	3871.919681	2880.340405	4863.498957	2910.36028	2867.106595	2863.55434	4936.039355	4933.302243	4721.155274
P2RX5	1.524955567	0.16336399	9.334710596	1.01E-20	9.46E-19	Yes	OK	215.2089071	325.9481152	104.4696991	324.690774	329.5881095	323.5654621	105.2888029	115.000861	93.11943342
MPZL3	1.864068821	0.199803455	9.32951243	1.06E-20	9.87E-19	Yes	OK	122.0327416	198.036907	46.02857621	193.35518	205.6051216	195.1504193	46.79502351	45.76564878	45.52505634
MICA	1.005807583	0.107873814	9.323927116	1.12E-20	1.03E-18	Yes	OK	2087.503807	2806.929605	1368.078009	2632.184196	3213.22577	2575.37885	1339.507548	1374.142942	1390.583539

Gene Symbol	Log2 Fold Change	LFC Standard Err	Wald Statistic	p Value	FDR Adj p Va	Significa	Status	Base Mean	KD	Control	siICAM1_1	siICAM1_2	siICAM1_3	siCon_1	siCon_2	siCon_3
SDC1	-0.89795996	0.096342461	-9.320500572	1.16E-20	1.06E-18	Yes	OK	2707.912788	1872.969412	3542.856165	1854.203212	1716.131191	2048.573832	3671.738094	3444.15844	3512.671961
FZD7	-1.042217025	0.112094164	-9.297692141	1.44E-20	1.31E-18	Yes	OK	1080.561479	695.0165916	1466.106365	624.7561241	767.6613334	692.6323173	1376.275066	1511.439888	1510.604142
GAN	0.978820264	0.105567474	9.271987159	1.83E-20	1.65E-18	Yes	OK	872.9469584	1166.444711	579.4492054	1193.877031	1139.610297	1165.846806	612.5134327	543.3203945	582.5137891
MIR4720	0.978820264	0.105567474	9.271987159	1.83E-20	1.65E-18	Yes	OK	872.9469584	1166.444711	579.4492054	1193.877031	1139.610297	1165.846806	612.5134327	543.3203945	582.5137891
PNMA2	-1.373040475	0.148377344	-9.253707085	2.17E-20	1.95E-18	Yes	OK	562.6733061	300.1746881	825.171924	275.4399262	340.9532167	284.1309214	753.7341286	989.2421005	732.5395429
MDGA1	-1.62413319	0.175720434	-9.242711023	2.40E-20	2.15E-18	Yes	OK	211.6860002	95.61412755	327.7578728	102.1499064	78.522559	106.1699173	295.8113986	327.4004105	360.0618092
KATNA1	-1.420371652	0.15369913	-9.241247205	2.44E-20	2.16E-18	Yes	OK	295.3249999	152.9789781	437.6710217	168.7297561	142.5804361	147.6267421	479.6489909	441.2277934	392.1362807
SDPR	-1.757399113	0.190668396	-9.217044598	3.05E-20	2.69E-18	Yes	OK	555.0297155	227.5781276	882.4813035	261.7591352	158.0783096	262.896938	1062.079909	640.7190829	944.644919
RHOD	1.274608875	0.138929781	9.174482715	4.54E-20	3.97E-18	Yes	OK	556.1286293	797.1776516	315.079607	742.410927	897.8434707	751.2785573	275.7563885	359.0843212	310.3981114
MGLL	0.942877691	0.102819506	9.170221909	4.72E-20	4.11E-18	Yes	OK	1435.601879	1900.522134	970.6816239	1980.06649	1871.109926	1850.389986	947.599226	897.710803	1066.734843
AGPAT5	0.989259603	0.108039866	9.156431233	5.36E-20	4.64E-18	Yes	OK	1896.410087	2540.395485	1252.42469	2684.171202	2729.692117	2207.323137	1301.068779	1206.335563	1249.869729
TBCC	-0.953059872	0.104480484	-9.121893749	7.38E-20	6.35E-18	Yes	OK	1147.487425	771.9370562	1523.037795	756.091718	830.6860189	729.0334318	1545.907027	1591.236404	1431.969954
NQO1	-1.090542776	0.120032154	-9.085422029	1.03E-19	8.83E-18	Yes	OK	6183.563936	3873.973907	8493.153966	3201.305103	4019.115191	4401.501427	9351.484073	7834.140288	8293.837537
CCDC115	-1.624416033	0.178817028	-9.084235709	1.04E-19	8.87E-18	Yes	OK	224.0375412	100.9255258	347.1495565	113.0945392	75.4229843	114.2590538	371.8533118	325.0534541	344.5419037
RAPGEF2	0.978300623	0.107882877	9.068173304	1.21E-19	1.02E-17	Yes	OK	2355.003884	3146.095399	1563.912369	2986.060657	3606.871756	2845.353782	1535.879522	1601.797707	1554.059878
TNFRSF10A	0.872616798	0.096492673	9.04334776	1.52E-19	1.27E-17	Yes	OK	1319.01573	1715.252456	922.7790036	1720.13146	1794.65375	1630.972157	950.1061023	921.1803664	897.050542
TUBB6	-0.801957419	0.088802642	-9.030783378	1.70E-19	1.42E-17	Yes	OK	3682.48085	2665.2674	4699.6943	2706.97252	2774.119354	2514.710326	4903.449963	4763.147907	4432.485031
ZNHIT6	0.95578897	0.106021807	9.015022425	1.97E-19	1.63E-17	Yes	OK	1275.744233	1695.060345	856.4281205	1821.369313	1651.040122	1612.7716	874.8998145	783.8834201	910.5011268
ANKRD46	1.44168298	0.161014242	8.953760624	3.44E-19	2.83E-17	Yes	OK	591.0402794	881.495715	300.5848438	966.7759	768.6945249	909.0167201	312.523907	222.960853	366.2697715
TMTC4	-1.566131777	0.17513844	-8.942250329	3.81E-19	3.12E-17	Yes	OK	226.6331405	106.1449111	347.1213699	89.38116811	119.8502216	109.2033435	313.3595324	398.9825791	329.0219981
ATP6V1B2	0.948878394	0.106179514	8.936548658	4.02E-19	3.27E-17	Yes	OK	2703.389729	3586.149464	1820.629995	3835.181754	3315.511735	3607.754902	1961.21286	1604.144664	1896.532461
UCN2	-1.882916509	0.211292923	-8.91140357	5.04E-19	4.08E-17	Yes	OK	131.7726576	48.0423362	215.5029791	41.95442585	49.59319516	52.57938759	175.4813381	255.8182419	215.2093572
TET3	1.370607502	0.153861269	8.908073527	5.19E-19	4.17E-17	Yes	OK	766.8269258	1124.86681	408.7870418	985.0169547	1196.435833	1193.147641	311.6882816	420.1051862	494.5676575
PPP2R1B	0.87721521	0.098478197	8.907709862	5.21E-19	4.17E-17	Yes	OK	2733.210403	3559.508854	1906.911952	3745.800586	3427.096424	3505.629553	2105.776058	1774.298999	1840.660801
LGALS1	-1.066118653	0.119772177	-8.90122131	5.52E-19	4.40E-17	Yes	OK	7506.893741	4759.69052	10254.09696	4359.612077	5361.231035	4558.228447	10293.23392	11758.2513	8710.805666
SH2D5	0.967606263	0.108874284	8.887371963	6.26E-19	4.95E-17	Yes	OK	929.9091318	1239.332308	620.4859554	1211.206033	1239.829879	1266.961013	593.294048	583.2186524	684.9451658
TSPAN9	-0.995906984	0.112346587	-8.864594917	7.68E-19	6.04E-17	Yes	OK	866.3504594	569.8159572	1162.884962	536.2870087	579.6204684	593.5403945	1186.588096	1241.539908	1060.526881
ISG20L2	0.972551476	0.110049152	8.837428134	9.79E-19	7.67E-17	Yes	OK	726.1892225	969.3498566	483.0285884	994.1374821	939.1711333	974.7409546	491.3477468	451.7890969	505.9489216
FAM222B	1.545542093	0.174970155	8.833175519	1.02E-18	7.92E-17	Yes	OK	235.1476591	358.2925695	112.0027486	345.6679869	339.9200251	389.2896966	86.90504365	129.0825991	120.0206031
FAM175A	-1.350491113	0.152964844	-8.828767966	1.06E-18	8.19E-17	Yes	OK	244.5192941	131.5924489	357.4461393	141.3681741	123.9829879	129.4261848	360.9901813	354.3904085	356.9578281
TNFSF10	-1.54481133	0.175462955	-8.804202173	1.32E-18	1.01E-16	Yes	OK	932.7116144	442.0219951	1423.401234	589.1860674	320.2893854	416.5905325	1701.333355	1240.36643	1328.503917
MGAT4A	-1.474252082	0.167852326	-8.783030418	1.59E-18	1.22E-16	Yes	OK	418.3331897	208.0905806	628.5757989	225.2770258	170.4766084	228.5181076	776.296015	562.0960453	547.3353364
WWC3	-0.888819962	0.101417361	-8.763982341	1.88E-18	1.44E-16	Yes	OK	1982.195222	1374.93437	2589.456074	1293.290779	1436.136276	1395.376055	2352.285557	2787.010663	2629.072004
PPP1R15B	0.845054291	0.096589313	8.748941905	2.15E-18	1.63E-16	Yes	OK	3751.542401	4844.40762	2658.677183	4851.208502	5321.969755	4360.044602	2626.370694	2697.826322	2651.834532
SLC26A2	-0.841307326	0.096322927	-8.734237593	2.45E-18	1.85E-16	Yes	OK	1361.809278	966.92686	1756.691697	927.5576324	1017.693692	955.5292553	1786.567147	1800.115519	1683.392424
BNIP3	-1.179197765	0.135219989	-8.720587628	2.77E-18	2.07E-16	Yes	OK	1584.63467	943.3874304	2225.88191	976.8084801	944.3370911	909.0167201	2527.766895	2430.273298	1719.605537
EMP2	-1.237583316	0.142036272	-8.71314979	2.96E-18	2.20E-16	Yes	OK	427.2149892	246.2500901	608.1798884	207.9480238	282.0612975	248.740949	641.7603224	596.1269123	586.6524306
ORAI3	-1.170615067	0.134413378	-8.709066649	3.06E-18	2.26E-16	Yes	OK	400.4550917	239.4212184	561.4889651	245.3421859	223.1693782	249.7520911	570.7321617	598.4738686	515.2608649
RNF130	-1.050600022	0.120635692	-8.708865566	3.07E-18	2.26E-16	Yes	OK	606.2779887	387.0160781	825.5398994	397.6549928	378.1481131	385.2451283	818.9129114	902.4047157	755.3020711
TCAF1	-1.2528625	0.143974263	-8.701989327	3.26E-18	2.39E-16	Yes	OK	469.9123566	267.9574107	671.8673026	246.2542387	259.331083	298.2869104	641.7603224	776.8425511	596.9990343
PJA1	-1.204874471	0.138619567	-8.691950899	3.56E-18	2.60E-16	Yes	OK	446.8184011	261.9869798	631.6498225	274.5278735	227.3021445	284.1309214	664.3222087	659.4947337	571.132525
TFPI2	-0.942059432	0.108396655	-8.690853331	3.60E-18	2.61E-16	Yes	OK	1401.512298	947.2847886	1855.739807	887.427312	989.79752	964.6295339	2066.501663	1753.176392	1747.541367
TGFBR2	0.795747365	0.091566398	8.690386208	3.61E-18	2.61E-16	Yes	OK	4847.991654	6178.19972	3517.783587	6315.053143	6059.668533	6159.877485	3173.705344	3703.497117	3676.148299
OTULIN	-0.80640961	0.092845715	-8.685480105	3.77E-18	2.71E-16	Yes	OK	1625.968168	1173.541029	2078.395307	1161.043133	1211.933707	1147.646248	2091.570426	2167.414187	1976.201309
ASAP3	-1.194697595	0.137711961	-8.675336468	4.12E-18	2.95E-16	Yes	OK	763.220997	450.2052966	1076.236697	412.2478366	427.7413082	510.6267449	1096.340551	1223.937735	908.431806
TTC30A	-1.993642847	0.229834124	-8.67426826	4.16E-18	2.96E-16	Yes	OK	100.1489837	32.43949086	167.8584766	31.92184575	31.02893854	33.36768828	194.7007228	179.5421606	129.3325464
ABHD5	1.232228785	0.142087648	8.672314619	4.23E-18	3.00E-16	Yes	OK	2855.677356	4062.492	1648.862713	3894.465182	4957.253133	3335.757686	1536.715147	1938.585943	1471.287048
MTMR9	0.940773534	0.108615888	8.661472596	4.66E-18	3.28E-16	Yes	OK	1010.076657	1338.04933	682.1039848	1348.925996	1414.439254	1250.782739	734.514744	637.1986484	674.5985621
CCNG2	-1.542887219	0.178748745	-8.631597482	6.05E-18	4.24E-16	Yes	OK	249.7297035	118.196923	381.262484	114.9186447	143.6136276	96.05849656	421.990837	398.9825791	322.8140359
DDAH2	-1.103902629	0.128798778	-8.570753878	1.03E-17	7.17E-16	Yes	OK	987.1187098	612.117966	1362.119454	652.1177061	530.0272732	654.2089187	1495.769501	1415.214678	1175.374182
ANTXR1	-1.210515572	0.141254465	-8.569750844	1.04E-17	7.19E-16	Yes	OK	688.5742025	402.2187222	974.9296827	330.1630904	465.9693962	410.52368	887.4341958	1085.467311	951.8875416
GSTP1	-0.750881102	0.087627456	-8.56901633	1.04E-17	7.20E-16	Yes	OK	4173.44884	3091.475905	5255.421775	2964.171391	3066.512567	3243.743758	5571.950299	5176.212225	5018.102801

KO_vs_Control_Differential_Expr

Gene Symbol	Log2 Fold Change	LFC Standard Err	Wald Statistic	p Value	FDR Adj p Va	Significa	Status	Base Mean	KD	Control	siICAM1_1	siICAM1_2	siICAM1_3	siCon_1	siCon_2	siCon_3
C2CD2	0.923261605	0.107794974	8.564978241	1.08E-17	7.43E-16	Yes	OK	1356.901353	1788.510583	925.2921222	1617.981553	1907.27163	1840.278566	866.5435603	991.5890569	917.7437494
C3orf14	1.184070954	0.138470117	8.551093748	1.22E-17	8.33E-16	Yes	OK	819.8456658	1154.093188	485.5981433	1120.912812	1353.480951	987.8858015	546.4990245	470.5647477	439.7306578
CPEB3	1.683323938	0.197035669	8.543244717	1.31E-17	8.88E-16	Yes	OK	133.8166916	210.6434977	56.9898856	190.6190218	210.7710794	230.5403917	50.97315061	61.02086504	58.97564117
PPIF	0.826425074	0.096751431	8.541734924	1.32E-17	8.95E-16	Yes	OK	3976.142462	5110.719143	2841.56578	5340.068768	4737.183329	5254.905333	2831.934548	2615.682849	3077.079944
RAD23B	-0.841298466	0.098625589	-8.530225049	1.46E-17	9.84E-16	Yes	OK	4444.741768	3154.267451	5735.216085	3303.455009	2960.093836	3199.253507	6184.463732	5133.96701	5887.217513
SLC25A1	-0.93067748	0.109160744	-8.525752415	1.52E-17	1.02E-15	Yes	OK	1005.96344	683.1562222	1328.770658	674.0069718	677.7736671	697.6880277	1407.193207	1388.22468	1190.894087
IRF2BPL	-1.298044984	0.152254402	-8.525500524	1.52E-17	1.02E-15	Yes	OK	700.2523053	388.0786467	1012.425964	375.7657272	321.322577	467.1476359	944.2567243	900.0577593	1192.963408
SFXN3	0.729196358	0.085581548	8.52048576	1.59E-17	1.06E-15	Yes	OK	2416.158268	3025.42684	1806.889696	3030.751241	2980.757667	3064.771611	1886.842198	1753.176392	1780.650499
C5	-1.835061493	0.215417621	-8.518622946	1.61E-17	1.07E-15	Yes	OK	151.2067194	56.48556078	245.927878	57.45932236	36.1617048	75.83565518	267.4001343	224.1343312	246.2491684
RASSF4	-1.10908	0.13052354	-8.497164604	1.94E-17	1.27E-15	Yes	OK	437.9027029	270.6074674	605.1979384	286.3845591	267.5966155	257.8412276	634.2396936	627.810823	553.5432987
KDM3A	-0.815219086	0.096098514	-8.48316017	2.19E-17	1.43E-15	Yes	OK	1220.756615	877.2465097	1564.26672	830.8800424	900.9430454	899.9164415	1577.660793	1572.460753	1542.678614
SFTA1P	1.407148138	0.165931659	8.480287284	2.25E-17	1.46E-15	Yes	OK	398.5836912	591.4782608	205.6891217	588.2740146	714.9685635	471.1922042	226.4544888	179.5421606	211.0707158
SLCO4A1-AS1	-1.823968558	0.21516499	-8.477069437	2.31E-17	1.49E-15	Yes	OK	104.8465382	39.40015483	170.2929216	37.39416217	43.39404576	37.41225656	149.5769501	185.4095515	175.8922631
TNFAIP2	-1.030009454	0.122023181	-8.441096596	3.14E-17	2.02E-15	Yes	OK	3749.582232	2417.563186	5081.601278	2373.161219	2310.216341	2569.311997	4777.270525	6112.647807	4354.885503
NLRP3	1.448939922	0.171678644	8.439837851	3.18E-17	2.03E-15	Yes	OK	314.8223724	470.9254589	158.719286	468.7951062	548.6247214	395.356549	132.0288163	188.929986	155.1990557
DCAF12	0.771550872	0.091428171	8.438874595	3.20E-17	2.04E-15	Yes	OK	1750.574439	2217.632884	1283.515995	2228.144834	2274.054636	2150.699181	1301.904404	1218.070344	1330.573238
PKP2	0.92710418	0.109998437	8.428339548	3.51E-17	2.22E-15	Yes	OK	794.759533	1048.881941	540.6371246	1057.069121	1115.846891	973.7298125	523.9371382	552.7082199	545.2660157
REV3L	0.797027078	0.094640421	8.421634951	3.71E-17	2.34E-15	Yes	OK	1610.860298	2054.171445	1167.549151	2030.22939	2167.635905	1964.64904	1116.395561	1192.253825	1193.998069
NFIA	-1.324587881	0.157601414	-8.404670044	4.29E-17	2.70E-15	Yes	OK	280.6202186	152.5366921	408.703745	153.2248596	143.6136276	160.771589	349.2914255	441.2277934	435.5920163
PM20D2	-0.992687459	0.118356864	-8.387240323	4.98E-17	3.11E-15	Yes	OK	575.1611291	378.449796	771.8724621	356.6126197	386.4136456	392.3231228	816.4060351	734.5973368	764.6140144
TMEM263	-0.869795787	0.103919756	-8.369879071	5.77E-17	3.59E-15	Yes	OK	1656.079613	1158.772692	2153.386534	1199.349348	1131.344765	1145.623964	2394.902453	2073.535933	1991.721215
TULP3	-1.190484895	0.14243393	-8.358155202	6.37E-17	3.95E-15	Yes	OK	351.8767205	207.51116	496.242281	197.0033909	229.3685276	196.1615614	489.676496	538.6264818	460.4238652
TBC1D9	0.784377339	0.093860825	8.356812715	6.44E-17	3.98E-15	Yes	OK	2207.951603	2806.175703	1609.727503	2865.669696	2921.865748	2630.991664	1568.468913	1709.757699	1550.955897
RABL3	-1.174238396	0.140555257	-8.35428303	6.58E-17	4.05E-15	Yes	OK	309.5889834	184.3652065	434.8127602	176.9382308	190.1072481	186.0501407	447.895225	444.7482279	411.7948278
PPME1	0.742363477	0.089097258	8.332057489	7.94E-17	4.84E-15	Yes	OK	2601.952522	3269.985295	1933.919749	3045.344085	3414.698125	3349.913675	1921.10284	1956.188116	1924.468291
TMEM200B	-1.279713908	0.153591151	-8.331950747	7.95E-17	4.84E-15	Yes	OK	292.5883513	163.5931953	421.5835072	155.0489651	143.6136276	192.1169931	416.9770845	431.839968	415.9334693
SUCO	0.82805166	0.099419399	8.328874118	8.16E-17	4.95E-15	Yes	OK	3503.731217	4506.883984	2500.57845	4635.052004	4865.299083	4020.300867	2637.233825	2453.742861	2410.758665
ROR1	0.744964505	0.089452183	8.328075199	8.22E-17	4.96E-15	Yes	OK	2671.158907	3359.357993	1982.959822	3375.507175	3208.059812	3494.506991	1953.692231	1901.034642	2094.152592
CDC25A	0.8568482	0.103071596	8.31313604	9.32E-17	5.61E-15	Yes	OK	2362.089653	3061.297784	1662.881522	2990.620921	3412.631742	2780.64069	1601.058304	1741.44161	1646.144651
CX3CL1	-2.27698953	0.274128392	-8.306288563	9.87E-17	5.91E-15	Yes	OK	98.48496872	20.72038454	176.2495529	18.24105472	20.66383132	23.25626759	120.3300604	286.3286744	122.0899238
NR6A1	1.321822839	0.159368617	8.294122533	1.09E-16	6.52E-15	Yes	OK	246.5947541	359.0355695	134.1539388	316.4822993	390.5464119	370.0779973	133.7000672	129.0825991	139.6791501
ZDHHC9	-1.115009807	0.13448802	-8.290774217	1.13E-16	6.67E-15	Yes	OK	647.0444289	397.7367634	896.3520945	435.049155	369.8825805	388.2785545	782.9810183	876.5881958	1029.487069
LBHD1	-0.858448502	0.103547471	-8.29038596	1.13E-16	6.67E-15	Yes	OK	1139.930774	801.7141673	1478.147381	849.1210971	752.1634599	803.8579449	1568.468913	1451.592501	1414.380728
TNFAIP1	0.843870384	0.101915116	8.280129754	1.23E-16	7.24E-15	Yes	OK	2005.565081	2590.357388	1420.772774	2570.16461	2823.712549	2377.195004	1392.987575	1514.960322	1354.370426
CCNDBP1	-0.993696597	0.1201418	-8.271031371	1.33E-16	7.79E-15	Yes	OK	583.209519	382.9319083	783.4871296	393.0947291	396.7455613	358.9554345	795.5153996	829.6490689	725.2969203
NDRG1	-1.0594194	0.128102899	-8.270065765	1.34E-16	7.82E-15	Yes	OK	3605.655575	2286.956079	4924.355071	2250.946152	1901.072481	2708.849603	5505.100265	4447.482279	4820.48267
MFSD6	-1.370901898	0.166034951	-8.256706757	1.50E-16	8.71E-15	Yes	OK	557.2184038	294.0638856	820.3729219	225.2770258	363.6834312	293.2312	787.1591454	961.0786243	712.8809959
ATG13	-0.816935194	0.098964904	-8.254797053	1.52E-16	8.81E-15	Yes	OK	1019.20303	731.2394783	1307.166581	757.9158235	709.8026057	726.0000056	1316.945662	1270.876862	1333.677219
ATP9B	-0.869313081	0.105398152	-8.247896835	1.61E-16	9.30E-15	Yes	OK	895.6049871	626.5044676	1164.705503	621.1079131	594.0851503	664.3203394	1168.204337	1119.498178	1206.413993
HIP1	-1.338999569	0.162407866	-8.244671925	1.66E-16	9.51E-15	Yes	OK	490.9502146	264.0887937	717.8116354	237.1337113	237.6340601	317.4986097	614.1846835	871.8942831	667.3559395
MORF4L2	-0.859832855	0.104418083	-8.234520579	1.80E-16	1.03E-14	Yes	OK	5666.89285	3982.209532	7351.576169	4379.677238	3722.589211	3844.362147	8222.55413	6855.459491	6976.714884
HSDL2	-0.853323579	0.103637911	-8.233701054	1.82E-16	1.03E-14	Yes	OK	1586.137682	1118.410925	2053.864439	1133.681551	1093.116677	1128.434549	2272.065516	2013.688546	1875.839253
MAPK8	0.872148445	0.106082264	8.221435085	2.01E-16	1.14E-14	Yes	OK	2031.476248	2644.30271	1418.649785	2568.340504	2959.060644	2405.506982	1468.193863	1455.112936	1332.642558
COL6A2	-1.120588242	0.136383566	-8.216446272	2.10E-16	1.18E-14	Yes	OK	792.3413855	485.4597196	1099.223051	442.3455769	514.5293998	499.5041821	963.476109	1311.948598	1022.244447
YRDC	1.129807636	0.137512405	8.216041566	2.10E-16	1.18E-14	Yes	OK	1070.243682	1488.328376	652.1589883	1721.955565	1317.319246	1425.710317	710.2816068	537.4530036	708.7423544
YME1L1	-0.71694057	0.087288264	-8.213481842	2.15E-16	1.20E-14	Yes	OK	4439.19308	3338.302762	5540.083399	3380.979492	3355.806206	3278.122588	5978.064253	5271.263957	5370.921988
ST6GALNAC4	0.960429203	0.117095378	8.202110301	2.36E-16	1.32E-14	Yes	OK	754.4048527	1004.95247	503.8572359	1037.003961	903.0094285	1074.844019	503.0465027	478.7790949	529.7461101
RASA2	0.860463747	0.104965073	8.197619655	2.45E-16	1.36E-14	Yes	OK	1257.44002	1632.18357	882.6964694	1701.890405	1679.969486	1514.690819	954.2842294	840.2103724	853.5948064
EFCAB14	0.794294161	0.096964982	8.191556821	2.58E-16	1.43E-14	Yes	OK	2309.063411	2943.762822	1674.364	3118.308304	2749.322757	2963.657404	1676.264592	1563.072928	1783.75448
EOGT	1.124347122	0.137745183	8.162514986	3.28E-16	1.81E-14	Yes	OK	1356.243063	1883.038412	829.4477135	1751.141253	2322.61464	1575.359344	797.1866505	896.5373248	794.6191652
SAMD4A	0.839146236	0.102892349	8.15557465	3.48E-16	1.91E-14	Yes	OK	2710.263198	3496.617682	1923.908714	3292.510376	3839.339858	3358.002811	1797.430278	2100.525931	1873.769933
COL18A1	-0.926186555	0.113593801	-8.153495576	3.54E-16	1.94E-14	Yes	OK	1589.213916	1079.868375	2098.559457	985.0169547	1077.618803	1176.969368	1902.719081	2342.262435	2050.696856

KO_vs_Control_Differential_Expr

Gene Symbol	Log2 Fold Change	LFC Standard Err	Wald Statistic	p Value	FDR Adj p Va	Significa	Status	Base Mean	KD	Control	siICAM1_1	siICAM1_2	siICAM1_3	siCon_1	siCon_2	siCon_3
ARHGEF40	-1.196227081	0.146759722	-8.150922245	3.61E-16	1.97E-14	Yes	OK	597.8672608	350.5283596	845.2061621	312.8340884	360.5838565	378.1671338	717.8022356	1013.885142	803.9311085
MICB	-0.785660906	0.096402168	-8.14982608	3.64E-16	1.98E-14	Yes	OK	1435.245833	1045.156602	1825.335064	1047.036541	1109.647742	978.7855228	1870.129689	1802.462475	1803.413027
IFT172	-1.070173611	0.131412978	-8.143591522	3.84E-16	2.08E-14	Yes	OK	424.0532538	267.2195974	580.8869103	263.5832407	243.8332095	294.2423421	593.294048	558.5756107	590.791072
FAM91A1	-0.692046429	0.085100461	-8.132111383	4.22E-16	2.27E-14	Yes	OK	2752.71923	2093.427996	3412.010464	2115.050294	2148.005265	2017.228428	3536.366776	3325.637145	3374.027471
WDR66	1.182197313	0.145557416	8.121862454	4.59E-16	2.46E-14	Yes	OK	605.2204152	853.058761	357.3820694	912.9647886	761.462184	884.7493104	382.7164422	282.8082399	406.6215259
KLHL5	-0.878713553	0.108474518	-8.10064491	5.47E-16	2.92E-14	Yes	OK	1513.623033	1053.720692	1973.525373	1022.411117	1145.809446	992.9415118	2167.612339	1935.065509	1817.898272
B4GAT1	-1.252433338	0.154859443	-8.087549041	6.09E-16	3.24E-14	Yes	OK	382.5216872	216.7957024	548.247672	217.9806039	172.5429915	259.8635117	540.6496466	525.7182219	578.3751476
BCL2L1	-0.729158213	0.090450751	-8.061383694	7.54E-16	4.00E-14	Yes	OK	4924.966159	3681.109056	6168.823263	3546.97309	3825.908368	3670.445711	5898.679838	6712.295154	5895.494796
PSIP1	-0.955202794	0.118603535	-8.053746388	8.03E-16	4.25E-14	Yes	OK	747.5997869	500.3349761	994.8645976	496.1566883	557.9234455	446.9247945	964.3117344	1037.354706	982.9273528
DRAM2	-1.17130981	0.145588407	-8.045350815	8.60E-16	4.53E-14	Yes	OK	451.2218698	268.4086094	634.0351302	282.7363481	224.2025698	298.2869104	698.5828509	570.3103925	633.2121473
METTL7A	-1.419987353	0.17661406	-8.040058378	8.98E-16	4.71E-14	Yes	OK	200.9134728	102.2616834	299.5652622	82.08474623	105.3855397	119.3147641	288.2907698	314.4921506	295.9128662
ZFP90	-1.141865388	0.142114961	-8.034800712	9.37E-16	4.90E-14	Yes	OK	325.0688619	196.7662687	453.3714552	179.674389	194.2400144	216.3844028	464.6077334	433.0134461	462.493186
COL1A1	1.512260125	0.188755869	8.011725053	1.13E-15	5.89E-14	Yes	OK	144.9085745	220.6260073	69.19114168	206.1239183	224.2025698	231.5515338	61.83628106	77.44955947	68.28758451
FBP1	-0.885471525	0.110572683	-8.008049527	1.17E-15	6.05E-14	Yes	OK	741.3098389	513.7827357	968.8369422	549.055747	490.7659937	501.5264663	981.8598682	998.6299259	926.0210324
MED7	-1.248236291	0.155955721	-8.003786493	1.21E-15	6.24E-14	Yes	OK	252.1957568	143.2131551	361.1783586	150.4887014	145.6800108	133.4707531	391.9083219	362.6047557	329.0219981
VAPB	0.851129239	0.106371926	8.001446149	1.23E-15	6.32E-14	Yes	OK	1356.335741	1756.108886	956.5625956	1586.059708	1927.935462	1754.33149	932.5579685	990.4155787	946.7142398
ANLN	-0.834636931	0.10431403	-8.001195353	1.23E-15	6.32E-14	Yes	OK	12599.57911	8957.48889	16241.66933	9364.957492	9693.40327	7814.10591	17641.72386	15122.61323	15960.67089
ECE2	1.150578926	0.144303864	7.973306437	1.54E-15	7.90E-14	Yes	OK	383.2839647	536.55399	230.0139394	487.0361609	580.65366	541.972149	246.5094988	198.3178114	245.214508
EHHADH	-1.696085009	0.213332695	-7.950422253	1.86E-15	9.47E-14	Yes	OK	112.4269745	46.12580204	178.728147	46.51468953	40.29447107	51.56824552	199.7144753	185.4095515	151.0604142
HADH	-0.90187592	0.113456399	-7.949097022	1.88E-15	9.54E-14	Yes	OK	660.899564	454.6919063	867.1072217	444.1696824	462.8698215	457.0362152	926.7085905	817.9142871	856.6987875
CDK5R1	1.320611704	0.16650964	7.931142628	2.17E-15	1.10E-13	Yes	OK	316.2168933	460.6897513	171.7440354	407.6875729	551.7242961	422.6573849	149.5769501	195.970855	169.6843009
PRR14L	0.704859327	0.088892437	7.929350889	2.20E-15	1.11E-13	Yes	OK	1983.156297	2467.254565	1499.058029	2447.949543	2474.4938	2479.320353	1481.563869	1579.501622	1436.108595
LRRC61	-1.066908764	0.134560287	-7.928853187	2.21E-15	1.11E-13	Yes	OK	776.0004846	489.0178206	1062.983149	456.9384207	425.6749251	584.4401159	1062.915534	1144.141219	981.8926924
LONP1	-0.714038522	0.090138274	-7.921590844	2.34E-15	1.17E-13	Yes	OK	2598.0879	1955.080776	3241.095025	1842.346526	2016.789936	2006.105865	3096.827806	3389.004966	3237.452302
FN3KRP	-0.955051179	0.120654736	-7.915571391	2.46E-15	1.23E-13	Yes	OK	535.4818549	358.0552407	712.908469	387.6224127	330.621301	355.9220083	719.4734864	714.6482078	704.6037129
ST14	1.778148683	0.224732942	7.91227432	2.53E-15	1.26E-13	Yes	OK	91.99203622	149.2146371	34.7694353	145.016385	156.0119264	146.6156	35.93189305	26.989998	41.38641485
IL6R	2.257192049	0.285515729	7.9056662	2.67E-15	1.32E-13	Yes	OK	58.06751959	105.4048457	10.73019344	93.02937906	129.1489457	94.03621242	19.21938465	4.693912695	8.277282971
CGN	1.589520927	0.201562964	7.885977142	3.12E-15	1.54E-13	Yes	OK	278.1535331	431.8250554	124.4820109	356.6126197	593.0519588	345.8105876	120.3300604	145.5112936	107.6046786
TMEM19	-1.038461238	0.131765897	-7.881107766	3.24E-15	1.60E-13	Yes	OK	1015.198074	649.8727391	1380.523409	635.7007569	731.4996286	582.4178318	1557.605782	1411.694243	1172.270201
PDK1	-1.086695919	0.137941907	-7.877924406	3.33E-15	1.63E-13	Yes	OK	919.1695931	572.3848323	1265.954354	591.0101728	603.3838744	522.7604497	1169.039962	1537.256408	1091.566692
SLC4A7	0.761972476	0.096731673	7.87717663	3.35E-15	1.63E-13	Yes	OK	2338.386557	2956.069192	1720.703921	2979.676288	3204.960237	2683.571051	1718.881488	1713.278134	1729.952141
ENO2	-0.834342969	0.105945453	-7.875212623	3.40E-15	1.66E-13	Yes	OK	1250.766316	889.1571919	1612.375439	939.4143179	900.9430454	827.1142125	1748.964004	1563.072928	1525.089387
DDAH1	0.775879463	0.098527903	7.874718125	3.42E-15	1.66E-13	Yes	OK	4687.135992	5948.415743	3425.856242	6408.082522	5739.379148	5697.785559	3637.477452	3075.686294	3564.404979
WBSCR22	-0.830215481	0.10546747	-7.871768212	3.50E-15	1.69E-13	Yes	OK	812.142805	578.4559154	1045.829695	580.9775927	600.2842997	554.1058538	1043.696149	1011.538186	1082.254748
PEA15	0.768472932	0.097651736	7.869526564	3.56E-15	1.71E-13	Yes	OK	4863.789813	6159.971689	3567.607938	6460.981581	6230.145142	5788.788345	3643.32683	3201.248458	3858.248525
KLHL8	-0.954127273	0.121319203	-7.864602234	3.70E-15	1.78E-13	Yes	OK	521.9235917	349.5307166	694.3164668	331.0751431	360.5838565	356.9331504	740.3641219	670.0560372	672.5292414
PPP2CB	0.948519226	0.12064969	7.861762631	3.79E-15	1.81E-13	Yes	OK	1474.941245	1959.483645	990.3988453	2103.193609	2068.449515	1706.807813	869.0504365	1020.926011	1081.220088
BRWD3	0.746684094	0.095092004	7.852227979	4.09E-15	1.95E-13	Yes	OK	1207.476805	1519.866272	895.0873379	1537.720913	1543.588199	1478.289705	869.0504365	895.3638466	920.8477305
CASP7	0.911191912	0.116151361	7.844866398	4.33E-15	2.06E-13	Yes	OK	1219.246493	1605.200747	833.2922392	1720.13146	1533.256284	1562.214497	953.4486039	783.8834201	762.5446937
MAK16	0.835494688	0.106525158	7.843167768	4.39E-15	2.08E-13	Yes	OK	1260.904057	1626.471762	895.3363522	1715.571196	1704.766084	1459.078006	929.2154668	856.6390669	900.1545231
CHIC2	0.915821116	0.116777076	7.842473432	4.42E-15	2.08E-13	Yes	OK	729.8068781	961.8849756	497.7287805	899.2839975	1065.220504	921.1504249	506.3890044	478.7790949	508.0182423
GNPDA1	0.741317365	0.09523776	7.783859699	7.03E-15	3.31E-13	Yes	OK	2418.041416	3040.02128	1796.061552	2915.832596	3262.818965	2941.412279	1873.472191	1836.493342	1678.219122
IGF2BP2	0.994581225	0.127807811	7.781850058	7.15E-15	3.35E-13	Yes	OK	1337.173717	1798.045694	876.3017414	1727.427882	2004.391638	1662.317562	770.446637	1033.834271	824.624316
ANKRD13C	0.692677038	0.089019013	7.781225812	7.18E-15	3.35E-13	Yes	OK	2416.160972	2996.713778	1835.608165	3052.640507	3130.570444	2806.930384	1860.93781	1808.329866	1837.55682
DUSP2	2.08639806	0.268262958	7.777436265	7.40E-15	3.45E-13	Yes	OK	53.75429134	93.72171045	13.78687223	84.82090443	103.3191566	93.02507035	10.86313046	12.90825991	17.58922631
CORO1C	-0.7912786	0.101875991	-7.767076328	8.03E-15	3.73E-13	Yes	OK	5007.794344	3633.191474	6382.397214	4022.152565	3441.561106	3435.860751	6736.812134	5778.206528	6632.17298
NEURL1B	-1.529075477	0.197027019	-7.760740052	8.44E-15	3.90E-13	Yes	OK	183.5688709	85.65894304	281.4787989	76.61242981	112.6178807	67.74651863	261.5507564	318.0125851	264.8730551
F11R	0.987813558	0.127380854	7.75480401	8.85E-15	4.08E-13	Yes	OK	1102.845418	1481.838155	723.8526807	1632.574397	1466.098832	1346.841236	842.3104231	636.0251702	693.2224488
ABL2	0.659408011	0.085109385	7.747770791	9.35E-15	4.30E-13	Yes	OK	8614.498971	10584.83917	6644.158768	10722.09196	11165.70125	9866.72431	6649.071465	6406.017351	6877.387488
FBXO45	0.881368743	0.113906458	7.737653842	1.01E-14	4.64E-13	Yes	OK	1939.940617	2533.899483	1345.981751	2647.689092	2767.920205	2186.089153	1430.590719	1233.325561	1374.028973
KCNN4	-1.132824806	0.146511646	-7.731977858	1.06E-14	4.83E-13	Yes	OK	1050.172241	636.7619067	1463.582576	601.0427529	512.4630166	796.7799504	1480.728244	1316.642511	1593.376972
MIR5572	-0.926694937	0.119877407	-7.73035518	1.07E-14	4.87E-13	Yes	OK	741.9849766	503.0706963	980.8992569	527.1664813	437.0400323	545.0055752	985.2023699	1013.885142	943.6102587

KO_vs_Control_Differential_Expr

Gene Symbol	Log2 Fold Change	LFC Standard Err	Wald Statistic	p Value	FDR Adj p Va	Significa	Status	Base Mean	KD	Control	siICAM1_1	siICAM1_2	siICAM1_3	siCon_1	siCon_2	siCon_3
ARNT2	-0.926694937	0.119877407	-7.73035518	1.07E-14	4.87E-13	Yes	OK	741.9849766	503.0706963	980.8992569	527.1664813	437.0400323	545.0055752	985.2023699	1013.885142	943.6102587
VPS33A	0.820407676	0.1061319	7.730076189	1.07E-14	4.87E-13	Yes	OK	1569.84819	2017.128129	1122.568251	2101.369503	1823.583114	2126.431771	1155.669956	1031.487315	1180.547484
GJB3	1.600765439	0.20713677	7.728060244	1.09E-14	4.94E-13	Yes	OK	156.7781303	244.9646032	68.59165732	244.4301332	239.7004433	250.7632331	78.54878946	43.41869243	83.80749008
DDX21	0.805476947	0.104251113	7.72631507	1.11E-14	4.99E-13	Yes	OK	13098.95661	16760.94186	9436.971363	17833.36714	17951.70346	14497.75499	10039.20379	8751.80022	9519.910077
REPIN1	-0.794669124	0.103106164	-7.70729015	1.29E-14	5.77E-13	Yes	OK	2885.58206	2089.52592	3681.6382	2001.955755	1971.329507	2295.292497	3358.378562	3717.578855	3968.957184
ELL2	0.79467265	0.103386987	7.686389519	1.51E-14	6.78E-13	Yes	OK	2259.961846	2883.661195	1636.262496	2882.998698	3194.628321	2573.356566	1694.648351	1595.930316	1618.208821
PPM1E	1.950781601	0.254107285	7.676999894	1.63E-14	7.27E-13	Yes	OK	82.03239512	138.6145794	25.45021087	115.8306975	175.6425662	124.3704745	18.38375923	35.20434521	22.76252817
LIMA1	0.811346255	0.105827796	7.666664954	1.77E-14	7.85E-13	Yes	OK	5337.83656	6841.467913	3834.205208	6711.796083	7836.758026	5975.849628	3809.616289	3877.171886	3815.82745
MTMR2	0.688427351	0.089927571	7.655353581	1.93E-14	8.55E-13	Yes	OK	3232.822582	4005.414008	2460.231155	4096.940889	4218.521163	3700.779973	2519.410641	2391.548518	2469.734306
FGD5-AS1	-1.087448257	0.142118745	-7.651687712	1.98E-14	8.77E-13	Yes	OK	3043.537452	1892.114278	4194.960626	1713.747091	2270.955062	1691.640682	3995.125132	5072.946145	3516.810602
FAM196B	1.230398987	0.160883367	7.647769989	2.04E-14	9.01E-13	Yes	OK	330.1403803	471.4755402	188.8052204	445.0817351	505.2306757	464.1142097	158.7688298	174.8482479	232.7985836
COL6A1	-0.771918633	0.100989774	-7.643532613	2.11E-14	9.28E-13	Yes	OK	1867.337774	1367.073828	2367.60172	1234.007352	1498.12777	1369.086362	2313.846787	2436.140689	2352.817684
ZNF367	0.731175441	0.096069375	7.610910775	2.72E-14	1.19E-12	Yes	OK	1625.28563	2038.08014	1212.491119	1994.659333	2188.299736	1931.281352	1243.410625	1214.54991	1179.512823
RFTN1	0.943256026	0.124004178	7.606647111	2.81E-14	1.23E-12	Yes	OK	1865.177399	2476.676698	1253.678099	2374.985324	2906.367875	2148.676897	1248.424377	1368.275551	1144.334371
TADA2B	0.800624838	0.105475538	7.590621052	3.18E-14	1.39E-12	Yes	OK	816.557232	1043.701677	589.4127865	990.4892711	1091.050293	1049.565468	582.4309176	586.7390869	599.068355
DAGLB	0.889021658	0.117260995	7.581563309	3.41E-14	1.48E-12	Yes	OK	1076.226127	1409.147919	743.3043356	1337.981363	1545.654582	1343.80781	737.8572456	665.3621245	826.6936367
PRKCE	1.028989948	0.135778797	7.578428802	3.50E-14	1.51E-12	Yes	OK	921.0328574	1250.685215	591.3805	1134.593603	1498.12777	1119.33427	574.0746634	654.800821	545.2660157
F3	0.889264128	0.117459727	7.570800249	3.71E-14	1.60E-12	Yes	OK	13018.64542	17044.25456	8993.036278	17975.64737	17471.26938	15685.84692	10040.87504	7336.585543	9601.648246
MARCKSL1	-1.112030113	0.146951804	-7.567311746	3.81E-14	1.64E-12	Yes	OK	370.6637943	227.0822229	514.2453656	202.4757074	260.3642746	218.4066869	494.6902485	567.9634361	480.0824123
HIST1H4H	-0.662171631	0.087574566	-7.561232217	3.99E-14	1.71E-12	Yes	OK	3573.063218	2751.995892	4394.130543	2604.822614	2695.596795	2955.568268	4478.116624	4344.216199	4360.058805
SLC8B1	0.683547572	0.090476538	7.554970468	4.19E-14	1.79E-12	Yes	OK	2054.262015	2541.290097	1567.233933	2620.32751	2577.812957	2425.729824	1494.098251	1619.39988	1588.20367
HSPB8	1.129581299	0.150154701	7.522783459	5.36E-14	2.28E-12	Yes	OK	387.3350149	540.4430402	234.2269896	559.0883271	546.5583383	515.6824552	256.5370039	185.4095515	260.7344136
MAPK9	-0.792472253	0.1054129	-7.517792	5.57E-14	2.37E-12	Yes	OK	1086.66058	787.5329741	1385.788186	753.3555598	829.6528273	779.5905352	1438.111347	1444.551632	1274.701577
COX7A2L	-0.883904743	0.117602552	-7.51603369	5.65E-14	2.39E-12	Yes	OK	1478.069503	1023.723181	1932.415825	963.127689	1128.24519	979.7966649	1984.610372	2133.38332	1679.253783
SH3D21	-1.350980102	0.179966981	-7.506822058	6.06E-14	2.56E-12	Yes	OK	165.4203041	87.27059256	243.5700157	74.78832434	92.98724092	94.03621242	246.5094988	253.4712855	230.7292628
CEP57	-0.75070746	0.100120283	-7.498055727	6.48E-14	2.73E-12	Yes	OK	1803.883575	1333.603306	2274.163844	1373.55142	1388.609464	1238.649035	2456.738734	2208.485923	2157.266874
WNT9A	-1.764342699	0.235324613	-7.497484769	6.51E-14	2.73E-12	Yes	OK	184.5952537	69.09155121	300.0989563	63.84369151	40.29447107	103.136491	288.2907698	240.5630256	371.4430733
PDE4DIP	-0.715553521	0.095701153	-7.476958172	7.61E-14	3.18E-12	Yes	OK	2969.321829	2230.975455	3707.668202	2255.506416	2014.723553	2422.696397	3646.669332	3681.201031	3795.134242
FPR1	-1.638284842	0.219179431	-7.474628586	7.74E-14	3.23E-12	Yes	OK	134.6885559	56.60324703	212.7738648	59.28342783	61.99149395	48.53481931	198.0432245	275.7673708	164.510999
LRPAP1	-0.789278262	0.105670524	-7.4692377	8.07E-14	3.35E-12	Yes	OK	2492.243755	1808.85539	3175.63212	1759.349727	1696.500551	1970.715893	3530.517398	2960.685433	3035.69353
BTN3A1	-1.032591285	0.138250351	-7.468995755	8.08E-14	3.35E-12	Yes	OK	547.7884241	350.5511049	745.0257434	337.4595123	299.6255541	414.5682483	734.514744	789.750811	710.8116751
FLVCR2	1.430185632	0.191587805	7.464909517	8.34E-14	3.44E-12	Yes	OK	160.1375632	240.2352934	80.03983349	230.7493422	215.9370372	274.0195007	66.85003358	91.53129756	81.73816934
F2RL3	-1.866082877	0.25020608	-7.458183585	8.77E-14	3.60E-12	Yes	OK	65.62097447	21.77190622	109.4700427	17.32900198	21.69702288	26.2896938	101.1106758	119.6947737	107.6046786
C2orf69	1.0260262	0.137614805	7.455783572	8.93E-14	3.66E-12	Yes	OK	1182.767775	1605.736896	759.7986553	1553.225809	1943.433335	1320.551542	765.4328845	815.5673308	698.3957507
ATP6V1G1	0.812798606	0.109177687	7.444731852	9.71E-14	3.97E-12	Yes	OK	2411.514224	3094.036041	1728.992406	3320.784011	3062.379801	2898.944312	1966.226613	1581.848578	1638.902028
ZNF107	0.957508212	0.128661491	7.442073033	9.91E-14	4.04E-12	Yes	OK	466.6675367	622.0315282	311.3035452	579.1534873	651.943878	634.9972194	284.1126427	323.879976	325.918017
EIF5A2	0.889386774	0.119710122	7.429503508	1.09E-13	4.43E-12	Yes	OK	974.291842	1276.400499	672.1831851	1356.222418	1171.639236	1301.339843	706.1034797	582.0451742	728.4009014
SGK1	0.913989631	0.123162304	7.421017668	1.16E-13	4.71E-12	Yes	OK	1216.475436	1603.794688	829.1561838	1590.619971	1860.77801	1359.986083	848.1598011	834.3429816	804.9657689
UGP2	-0.880308018	0.118757939	-7.412624568	1.24E-13	5.00E-12	Yes	OK	2086.954576	1447.420497	2726.488655	1431.010743	1619.011164	1292.239564	2879.565196	2941.909782	2357.990986
BSN	2.020958454	0.272893482	7.405667744	1.30E-13	5.25E-12	Yes	OK	69.33206869	120.4346643	18.22947306	76.61242981	168.4102252	116.2813379	19.21938465	19.94912895	15.51990557
DNMT3B	0.976538024	0.132042105	7.395656312	1.41E-13	5.65E-12	Yes	OK	409.584232	548.9013802	270.2670838	529.9026395	592.0187672	524.7827338	266.5645089	288.6756308	255.5611117
ARID3A	1.490654896	0.201619027	7.39342372	1.43E-13	5.73E-12	Yes	OK	125.2627084	191.1474003	59.37801644	172.3779671	220.0698035	180.9944304	57.65815396	56.32695234	64.14894302
RASAL2	0.798754251	0.108081329	7.390307445	1.46E-13	5.85E-12	Yes	OK	2899.294956	3705.283919	2093.305993	3926.387028	3375.436845	3814.027884	2104.940432	1855.268993	2319.708553
LAMA1	1.659608082	0.224664547	7.387049298	1.50E-13	5.98E-12	Yes	OK	116.2482365	184.735646	47.76082687	215.2444457	152.9123517	186.0501407	42.61689641	37.55130156	63.11428265
GPCPD1	1.099880829	0.149115943	7.376010938	1.63E-13	6.47E-12	Yes	OK	752.5336897	1041.415438	463.6519419	993.2254293	1257.394136	873.6267477	442.8814725	536.2795254	411.7948278
POLR3G	1.049121027	0.142240187	7.375700584	1.63E-13	6.47E-12	Yes	OK	439.9299816	601.3141962	278.5457671	591.0101728	690.1719659	522.7604497	297.4826494	272.2469363	265.9077154
RARRES3	-1.427569877	0.193945079	-7.360691403	1.83E-13	7.22E-12	Yes	OK	413.7666838	205.5725763	621.9607912	201.5636546	216.9702288	198.1838455	542.3208974	876.5881958	446.9732804
ZCCHC2	1.030908247	0.140091396	7.358826264	1.86E-13	7.30E-12	Yes	OK	374.153444	509.0684523	239.2384357	488.8602664	516.5957829	521.7493076	239.8244955	208.8791149	269.0116965
ZDHHC20	-0.807704946	0.109888811	-7.350201925	1.98E-13	7.75E-12	Yes	OK	761.4333512	547.7395221	975.1271804	528.9905868	566.188978	548.0390014	1036.175521	917.6599319	971.5460887
CST1	1.426072852	0.194053112	7.348879066	2.00E-13	7.80E-12	Yes	OK	398.8537313	599.7239781	197.9834845	771.5966145	533.1268479	494.4484718	263.2220072	163.1134662	167.6149802
KRCC1	-1.656196081	0.225557743	-7.342670037	2.09E-13	8.15E-12	Yes	OK	283.304841	116.3888422	450.2208399	178.7623362	72.3234096	98.0807807	542.3208974	369.6456247	438.6959974
ETV1	-0.809877492	0.110335475	-7.340136919	2.13E-13	8.28E-12	Yes	OK	1349.453429	969.2968516	1729.610006	1005.082115	892.6775128	1010.130927	1865.951562	1544.297277	1778.581178

Gene Symbol	Log2 Fold Change	LFC Standard Err	Wald Statistic	p Value	FDR Adj p Va	Significa	Status	Base Mean	KD	Control	siICAM1_1	siICAM1_2	siICAM1_3	siCon_1	siCon_2	siCon_3
BDNF	0.762009622	0.1038308	7.338955518	2.15E-13	8.33E-12	Yes	OK	1076.971332	1362.803184	791.1394792	1312.443887	1439.235851	1336.729815	793.8441488	740.4647277	839.1095612
POFUT1	-0.615636592	0.084045429	-7.325045498	2.39E-13	9.22E-12	Yes	OK	3496.146875	2748.45145	4243.8423	2781.760844	2624.306577	2839.28693	4341.074056	4096.612305	4293.840541
MIR1825	-0.615636592	0.084045429	-7.325045498	2.39E-13	9.22E-12	Yes	OK	3496.146875	2748.45145	4243.8423	2781.760844	2624.306577	2839.28693	4341.074056	4096.612305	4293.840541
RNF213	-0.777145562	0.106154192	-7.320912575	2.46E-13	9.48E-12	Yes	OK	5400.249722	3939.35235	6861.147093	3736.680059	3663.697292	4417.6797	6145.189337	7194.594684	7243.65726
PNP	0.942920867	0.128835622	7.318790054	2.50E-13	9.61E-12	Yes	OK	3050.673898	4053.864234	2047.483563	4054.074411	4746.482053	3361.036238	2089.063549	2253.078094	1800.309046
CTNNB1	-0.63990577	0.087462937	-7.316307854	2.55E-13	9.76E-12	Yes	OK	14455.38774	11240.59558	17670.17991	11644.17728	11055.14975	11022.45969	18707.14627	18177.17691	16126.21655
SNRNP27	-1.230729047	0.168271687	-7.31394015	2.59E-13	9.91E-12	Yes	OK	222.4711254	126.3215192	318.6207316	131.335594	137.4144782	110.2144855	312.523907	356.7373648	286.6009229
LMBR1L	1.036709065	0.141767877	7.312721923	2.62E-13	9.97E-12	Yes	OK	324.0115011	441.5613144	206.4616878	435.049155	418.4425841	471.1922042	221.4407362	206.5321586	191.4121687
CRY2	0.897824886	0.12278424	7.312216032	2.63E-13	9.98E-12	Yes	OK	503.2636211	660.7699826	345.7572596	686.7757101	628.180472	667.3537656	338.428295	327.4004105	371.4430733
UBAC2	-0.858681037	0.117536483	-7.305655375	2.76E-13	1.04E-11	Yes	OK	598.8006979	419.9143554	777.6870404	417.720153	414.3098179	427.7130952	843.981674	745.1586404	743.920807
FAT3	-1.479477636	0.202645316	-7.300823268	2.86E-13	1.08E-11	Yes	OK	253.202296	120.783928	385.620664	143.1922795	82.65532526	136.5041793	427.0045895	292.1960653	437.6613371
PUDP	0.809272356	0.110919372	7.296041596	2.96E-13	1.12E-11	Yes	OK	841.4438473	1078.487296	604.4003983	1063.45349	1140.643489	1031.36491	613.3490581	644.2395174	555.6126194
TNRC6A	0.690100682	0.094592985	7.295474223	2.98E-13	1.12E-11	Yes	OK	1737.554033	2154.404441	1320.703625	2152.444457	2097.378879	2213.389989	1263.465635	1279.091209	1419.554029
PDGFRB	-1.82577601	0.250516459	-7.288048125	3.14E-13	1.18E-11	Yes	OK	90.99479834	31.09190169	150.897695	22.8013184	33.0621301	37.41225656	129.5219401	204.1852022	118.9859427
CEP164	-0.69105825	0.09483035	-7.287310945	3.16E-13	1.18E-11	Yes	OK	1724.821577	1310.215987	2139.427168	1313.35594	1253.261369	1364.030651	2073.186666	2068.84202	2276.252817
LAMC2	0.789007098	0.108358203	7.281470834	3.30E-13	1.23E-11	Yes	OK	4811.521742	6133.444974	3489.598511	6446.388737	6738.475392	5215.470792	3624.943071	3547.424519	3296.427943
PRTG	0.847181778	0.116352139	7.281187819	3.31E-13	1.23E-11	Yes	OK	930.9357817	1205.452769	656.4187771	1116.352549	1348.314993	1151.690817	612.5134327	693.5256007	663.217298
GLUL	-0.872284946	0.119869344	-7.276964351	3.41E-13	1.27E-11	Yes	OK	1809.920346	1259.68565	2360.155042	1430.09869	1140.643489	1208.314773	2672.330092	2155.679405	2252.455628
PRKCDBP	-0.867206919	0.119316696	-7.268110393	3.65E-13	1.35E-11	Yes	OK	1479.274271	1031.766736	1926.781807	985.9290074	953.6358152	1155.735385	1789.909649	2193.230707	1797.205065
ATP13A3	0.688794499	0.094863706	7.260885444	3.85E-13	1.42E-11	Yes	OK	10104.82942	12526.5875	7683.071337	13262.15883	12491.28603	11826.31764	8469.063629	7117.145124	7463.005259
NFATC2	1.490474871	0.205329791	7.258931431	3.90E-13	1.43E-11	Yes	OK	311.0015817	474.8984719	147.1046915	359.3487779	644.711537	420.6351007	111.9738062	151.3786844	177.9615839
DHTKD1	-1.040884183	0.143556643	-7.250686312	4.15E-13	1.52E-11	Yes	OK	780.6361112	496.2497747	1065.022448	555.4401161	380.2144962	553.0947118	1092.162424	1114.804265	988.1006546
NONO	-0.568325074	0.078425701	-7.246668694	4.27E-13	1.56E-11	Yes	OK	6545.62993	5252.975067	7838.284792	5487.821312	5160.791871	5110.312017	7904.180845	7869.344634	7741.328898
PIKFYVE	-0.684609636	0.09452086	-7.242947575	4.39E-13	1.60E-11	Yes	OK	1320.638224	1006.246256	1635.030192	966.7759	1059.021355	992.9415118	1627.798318	1648.736834	1628.555424
IDH2	-0.989635675	0.136741107	-7.237294615	4.58E-13	1.67E-11	Yes	OK	445.5425124	291.6565868	599.4284379	274.5278735	323.3889601	277.0529269	642.5959478	620.7699539	534.919412
NHLRC3	0.80600912	0.111533478	7.22661153	4.95E-13	1.80E-11	Yes	OK	812.0414435	1040.465966	583.6169207	1093.55123	1018.726884	1009.119785	634.2396936	570.3103925	546.3006761
TMTC3	0.658772546	0.091216548	7.22207277	5.12E-13	1.85E-11	Yes	OK	3454.601526	4246.702148	2662.500904	4396.094187	4362.134791	3981.877468	2838.619551	2531.192421	2617.690739
LIPH	1.200674402	0.166547204	7.209213801	5.63E-13	2.03E-11	Yes	OK	587.2225409	835.4909029	338.954179	967.6879527	702.5702647	836.2144911	403.6070777	251.1243292	362.13113
FAM136A	-0.67525669	0.093726168	-7.204569483	5.82E-13	2.10E-11	Yes	OK	1556.434274	1190.484565	1922.383983	1221.238613	1199.535408	1150.679675	1880.99282	2028.943763	1857.215367
MORC4	-0.78714028	0.109269997	-7.203626805	5.86E-13	2.11E-11	Yes	OK	5577.793325	4048.32065	7107.266	4037.657462	3504.585791	4602.718698	7554.053795	6660.662115	7107.082091
RARRES2	-1.339627165	0.185994777	-7.202498825	5.91E-13	2.12E-11	Yes	OK	394.2987478	207.953875	580.6436206	231.6613949	136.3812867	255.8189435	664.3222087	529.2386564	548.3699968
PJA2	-0.852291717	0.118532157	-7.190383923	6.46E-13	2.31E-11	Yes	OK	2477.258115	1740.851001	3213.665229	1640.782872	1956.864826	1624.905305	3278.158522	3592.01669	2770.820474
SACS	0.699466342	0.097357533	7.184511776	6.74E-13	2.40E-11	Yes	OK	8675.767269	10788.64999	6562.884543	10873.49272	11590.34298	9902.114282	7040.979787	5982.39173	6665.282112
ALDOA	-0.655327426	0.091217011	-7.184267693	6.76E-13	2.40E-11	Yes	OK	13162.91613	10162.62152	16163.21075	10192.18932	9477.466233	10818.209	17471.25628	15684.70927	15333.6667
FAM20C	-1.257797716	0.175317388	-7.17440368	7.26E-13	2.58E-11	Yes	OK	663.3929982	369.570448	957.2155483	407.6875729	251.0655505	449.9582207	1001.079253	803.8325491	1066.734843
SCN5A	-0.946658708	0.131955696	-7.174064736	7.28E-13	2.58E-11	Yes	OK	526.9019795	352.7946621	701.009297	331.9871958	350.2519408	376.1448497	693.5690984	623.1169103	786.3418822
COL6A3	-1.092337862	0.152381912	-7.168422072	7.59E-13	2.68E-11	Yes	OK	763.3277837	470.9785017	1055.677066	383.9742018	550.6911046	478.2701987	859.0229315	1160.569914	1147.438352
CTSW	-1.918608798	0.267839848	-7.163268696	7.88E-13	2.77E-11	Yes	OK	62.3233735	18.30960422	106.3371428	18.24105472	13.43149036	23.25626759	97.76817411	138.4704245	82.77282971
SFT2D2	-0.747650172	0.104395822	-7.161686736	7.97E-13	2.80E-11	Yes	OK	2225.794309	1645.49381	2806.094808	1680.913192	1643.807781	1611.760458	2468.43749	3092.114988	2857.731946
CDKN1B	0.909930024	0.12708808	7.159837659	8.08E-13	2.83E-11	Yes	OK	903.3928016	1190.838996	615.9466074	1180.19624	1372.078399	1020.242348	655.1303291	592.6064778	600.1030154
FAM179A	-1.664852589	0.232870793	-7.149254613	8.73E-13	3.05E-11	Yes	OK	127.0072393	51.10585092	202.9086277	34.65800396	64.05787708	54.60167173	155.4263281	254.6447637	198.6547913
BARD1	-0.818341313	0.114526811	-7.145412545	8.97E-13	3.13E-11	Yes	OK	971.1309355	694.3835946	1247.878276	670.3587608	768.6945249	644.097498	1349.535053	1171.131217	1222.968559
DCBLD1	0.899909115	0.126195086	7.131094742	9.96E-13	3.46E-11	Yes	OK	916.4649466	1205.203119	627.7267746	1269.577408	1098.282634	1247.749313	691.8978476	528.0651782	663.217298
GTF2E1	0.96783637	0.13597174	7.11792294	1.10E-12	3.80E-11	Yes	OK	454.3831733	608.051335	300.7150117	588.2740146	675.707284	560.1727063	274.9207631	299.2369343	327.9873377
TGFB2	0.763564506	0.107281215	7.117411075	1.10E-12	3.81E-11	Yes	OK	2231.695208	2826.322709	1637.067707	3041.695874	2812.347442	2624.924811	1793.252151	1456.286414	1661.664556
VPS36	0.823344086	0.115714797	7.115287841	1.12E-12	3.85E-11	Yes	OK	1055.030313	1358.042541	752.0180838	1366.254998	1517.75841	1190.114215	751.2272524	773.3221165	731.5048825
ADAM19	1.059845415	0.149025217	7.111852862	1.14E-12	3.94E-11	Yes	OK	2153.433961	2953.216926	1353.650996	2555.571766	3724.655595	2579.423418	1116.395561	1477.409021	1467.148407
PIGV	-1.026306699	0.144340749	-7.110304671	1.16E-12	3.98E-11	Yes	OK	339.1111816	217.1506575	461.0717057	229.8372894	188.040865	233.573818	471.2927367	431.839968	480.0824123
MEST	1.269456353	0.178689967	7.104239687	1.21E-12	4.15E-11	Yes	OK	890.2950601	1288.635225	491.954895	1106.319969	1714.064808	1045.520899	428.6758403	621.9434321	425.2454126
HIST1H2AI	-0.763297726	0.107511421	-7.099689672	1.25E-12	4.27E-11	Yes	OK	1666.54513	1223.419794	2109.670467	1107.232021	1363.812867	1199.214494	2180.14672	2176.802012	1972.062668
CALHM2	-1.006428382	0.141776134	-7.098715091	1.26E-12	4.29E-11	Yes	OK	304.8837763	197.4024137	412.3651388	197.9154437	187.0076734	207.2841242	425.3333387	424.7990989	386.9629789
RBMX2	-0.995410545	0.140320499	-7.09383558	1.30E-12	4.44E-11	Yes	OK	464.6063341	302.8765937	626.3360745	280.9122426	274.8289565	352.8885821	655.9659545	569.1369143	653.9053547

KO_vs_Control_Differential_Expr

Gene Symbol	Log2 Fold Change	LFC Standard Err	Wald Statistic	p Value	FDR Adj p Va	Significa	Status	Base Mean	KD	Control	siICAM1_1	siICAM1_2	siICAM1_3	siCon_1	siCon_2	siCon_3
WARS	-0.651088645	0.091805552	-7.09204	1.32E-12	4.48E-11	Yes	OK	2361.6077	1826.651307	2896.564093	1794.007731	1721.297149	1964.64904	2968.977116	2825.735443	2894.979719
HIST1H2AG	-0.587247439	0.082824151	-7.090292323	1.34E-12	4.53E-11	Yes	OK	6212.106306	4943.781686	7480.430927	4929.645037	5050.240373	4851.459647	7568.259427	7030.307739	7842.725615
PRKAG2	0.714955011	0.100890447	7.086449027	1.38E-12	4.65E-11	Yes	OK	1769.01295	2209.622003	1328.403896	2197.135041	2411.469115	2020.261854	1343.685675	1364.755116	1276.770898
C7orf43	0.92499838	0.130765882	7.073698185	1.51E-12	5.08E-11	Yes	OK	501.4289153	663.6702381	339.1875924	658.5020753	655.0434527	677.4651863	320.0445358	307.4512815	390.06696
MIR4658	0.92499838	0.130765882	7.073698185	1.51E-12	5.08E-11	Yes	OK	501.4289153	663.6702381	339.1875924	658.5020753	655.0434527	677.4651863	320.0445358	307.4512815	390.06696
STX12	0.810834784	0.11465747	7.071800784	1.53E-12	5.14E-11	Yes	OK	903.4116006	1159.072692	647.7505088	1299.675149	1068.320079	1109.22285	668.5003358	630.1577793	644.5934113
ANGPTL4	1.544496483	0.218523964	7.067858634	1.57E-12	5.27E-11	Yes	OK	183.9954844	285.7933377	82.19763105	208.8600765	396.7455613	251.7743752	81.89129114	71.5821686	93.11943342
LINC01505	-2.052218945	0.290709556	-7.059344635	1.67E-12	5.59E-11	Yes	OK	44.68092533	9.704083077	79.65776759	11.85668557	3.099574697	14.15598897	86.06941824	68.06173408	84.84215045
GBP4	-1.537918362	0.217927881	-7.057006	1.70E-12	5.68E-11	Yes	OK	188.1837663	84.86647288	291.5010598	82.08474623	76.45617587	96.05849656	380.209566	190.1034642	304.1901492
POP1	0.828347399	0.11768711	7.03855673	1.94E-12	6.47E-11	Yes	OK	1214.599032	1566.411228	862.7868361	1673.61677	1576.650329	1448.966585	904.1467042	741.6382058	942.5755983
TPM1	0.644302588	0.091691522	7.026850184	2.11E-12	7.01E-11	Yes	OK	3049.262611	3733.739192	2364.78603	3841.566123	3816.609644	3543.04181	2446.711229	2186.189838	2461.457023
GPX8	-0.684377655	0.097555716	-7.015249201	2.30E-12	7.60E-11	Yes	OK	1355.695204	1032.57981	1678.810598	1015.114695	1026.992416	1055.63232	1746.457127	1729.706828	1560.26784
CSNK1G1	-0.901312972	0.128484672	-7.014945475	2.30E-12	7.60E-11	Yes	OK	686.7759502	470.0985872	903.4533133	521.6941649	417.4093926	471.1922042	888.2698212	822.6081998	999.4819187
BHLHE40	-1.241373118	0.177208562	-7.005153161	2.47E-12	8.13E-11	Yes	OK	818.7075311	459.5568635	1177.858199	387.6224127	621.9813226	369.0668552	1048.709902	1439.857719	1045.006975
B3GNT5	0.819522637	0.117111231	6.997814245	2.60E-12	8.52E-11	Yes	OK	1289.354744	1659.000687	919.7088013	1814.984944	1666.537996	1495.47912	1031.997393	850.771676	876.3573345
ZNF185	0.661889351	0.094587475	6.997642666	2.60E-12	8.52E-11	Yes	OK	2215.326822	2726.032057	1704.621587	2793.61753	2897.06915	2487.40949	1708.853983	1705.063787	1699.94699
VEZF1	-0.631717066	0.090391338	-6.988690289	2.77E-12	9.06E-11	Yes	OK	1752.310178	1367.013093	2137.607264	1375.375526	1309.053714	1416.610039	2166.776713	2071.188977	2174.856101
ZFYVE28	-1.018228487	0.145860393	-6.980842877	2.93E-12	9.56E-11	Yes	OK	580.6793931	372.9237854	788.4350008	358.4367252	329.5881095	430.7465214	667.6647104	802.6590709	894.9812212
NAB1	-0.798685888	0.114507715	-6.974952605	3.06E-12	9.95E-11	Yes	OK	1162.740486	838.2835654	1487.197407	844.5608334	894.743896	775.545967	1492.427	1641.695965	1327.469256
CCHCR1	-0.714928934	0.10254702	-6.971718289	3.13E-12	1.02E-10	Yes	OK	1143.901765	858.6241597	1429.17937	826.3197787	865.8145321	883.7381684	1403.01508	1538.429886	1346.093143
ZNF267	0.796206364	0.114235717	6.969854863	3.17E-12	1.03E-10	Yes	OK	724.9006253	926.3987128	523.4025377	990.4892711	947.4366658	841.2702015	528.9508907	528.0651782	513.1915442
RBM24	1.272092213	0.182550497	6.968440146	3.20E-12	1.03E-10	Yes	OK	297.5041225	431.898049	163.1101961	399.4790983	537.2596142	358.9554345	172.9744619	129.0825991	187.2735272
CDON	-1.003872103	0.14407346	-6.967779522	3.22E-12	1.04E-10	Yes	OK	585.2933622	379.208436	791.3782883	335.6354068	338.8868336	463.1030676	767.1041353	745.1586404	861.8720893
B3GNT3	1.674715837	0.240667307	6.95863456	3.44E-12	1.10E-10	Yes	OK	116.9019679	187.8488622	45.95507366	151.4007541	265.5302324	146.6156	37.60314389	51.63303965	48.62903745
ALDH16A1	-0.749639989	0.107761515	-6.956472225	3.49E-12	1.12E-10	Yes	OK	984.6730198	726.964095	1242.381945	697.7203429	702.5702647	780.6016773	1212.492484	1333.071205	1181.582144
CAV1	-0.644322827	0.092683543	-6.951857987	3.61E-12	1.15E-10	Yes	OK	13317.67259	10329.37978	16305.96539	10697.46654	10285.42204	10005.25077	18128.05786	15475.83016	15314.00816
STMN3	1.117728577	0.160818546	6.950246756	3.65E-12	1.16E-10	Yes	OK	1173.111133	1634.355241	711.8670251	1441.955375	1893.84014	1567.270207	604.9928039	938.782539	591.8257324
YEATS2	-0.669244357	0.0965183	-6.933859735	4.10E-12	1.30E-10	Yes	OK	1598.463073	1225.830232	1971.095914	1163.779291	1185.070726	1328.640679	1972.911616	1936.238987	2004.137139
GMFB	-0.876131297	0.1264724	-6.927450555	4.28E-12	1.36E-10	Yes	OK	3195.625987	2215.989983	4175.261992	2267.363101	2479.659758	1900.94709	4789.804906	4170.54143	3565.43964
WT1	-1.614044047	0.233266528	-6.919312679	4.54E-12	1.44E-10	Yes	OK	94.82566698	39.45142579	150.1999082	33.74595123	32.02893854	52.57938759	167.125084	157.2460753	126.2285653
DYNLL2	-0.704741275	0.101857283	-6.918909028	4.55E-12	1.44E-10	Yes	OK	1470.648137	1109.390443	1831.905831	1101.759705	1131.344765	1095.066861	1819.992164	1675.726832	1999.998498
TCEA3	-1.26998556	0.183717401	-6.912712422	4.75E-12	1.50E-10	Yes	OK	191.8236523	105.1874699	278.4598348	103.0619592	102.285965	110.2144855	228.961365	333.2678014	273.150338
GNG11	-0.949992633	0.137442227	-6.911941505	4.78E-12	1.50E-10	Yes	OK	1534.054394	1023.232141	2044.876647	1044.300383	856.515808	1168.880232	2414.121838	1791.901171	1928.606932
BTBD10	0.73369434	0.106198337	6.908717786	4.89E-12	1.53E-10	Yes	OK	2425.799046	3047.421671	1804.176421	3151.142202	3274.184072	2716.93874	1967.897864	1742.615088	1702.016311
CTGF	0.975663238	0.141411424	6.899465472	5.22E-12	1.63E-10	Yes	OK	2744.145669	3684.276989	1804.014348	3103.71546	4667.959494	3281.156014	1748.128378	1654.604225	2009.310441
ACBD3	-0.678472424	0.098364896	-6.897505611	5.29E-12	1.65E-10	Yes	OK	1170.42457	893.6576654	1447.191475	869.1862573	939.1711333	872.6156056	1468.193863	1487.970324	1385.410237
ARL3	-0.985570228	0.142972543	-6.893423076	5.45E-12	1.70E-10	Yes	OK	364.4721585	238.5351124	490.4092046	247.1662914	250.0323589	218.4066869	549.8415262	450.6156187	470.770469
MARS2	0.856269926	0.124375748	6.884540897	5.80E-12	1.80E-10	Yes	OK	604.5487483	785.8793821	423.2181146	735.1145051	883.3787887	739.1448525	419.4839607	408.3704045	441.7999786
IPO11	-0.933329169	0.135588153	-6.883559881	5.84E-12	1.81E-10	Yes	OK	1227.388204	825.7202601	1629.056149	767.0363508	960.8681562	749.2562732	1710.525234	1821.238126	1355.405086
IPO11-LRRC70	-0.933329169	0.135588153	-6.883559881	5.84E-12	1.81E-10	Yes	OK	1227.388204	825.7202601	1629.056149	767.0363508	960.8681562	749.2562732	1710.525234	1821.238126	1355.405086
ATL1	1.006779652	0.14632525	6.880423274	5.97E-12	1.85E-10	Yes	OK	405.6808427	549.6470902	261.7145952	587.3619619	515.5625913	546.0167173	305.8389036	255.8182419	223.4866402
GDPD5	0.984877823	0.143443766	6.865950686	6.60E-12	2.04E-10	Yes	OK	452.8530396	609.0897799	296.6162994	556.3521689	659.176219	611.7409518	269.0713852	349.6964958	271.0810173
FLOT1	-0.643166952	0.093689757	-6.864858808	6.66E-12	2.05E-10	Yes	OK	1998.829249	1550.572523	2447.085975	1533.160649	1620.044375	1498.512546	2379.861195	2616.856328	2344.540401
SERBP1	0.634433921	0.092463634	6.861442646	6.82E-12	2.09E-10	Yes	OK	7155.341381	8736.940534	5573.742228	8859.680276	9473.333467	7877.80786	5475.01775	5724.226532	5521.982402
NEK7	-0.82400917	0.120095811	-6.861264852	6.83E-12	2.09E-10	Yes	OK	2074.882917	1477.14412	2672.621713	1599.740499	1491.928621	1339.763242	3114.375939	2380.987215	2522.501985
PLAGL2	1.014688152	0.14833941	6.840314072	7.90E-12	2.42E-10	Yes	OK	358.2327423	485.7268991	230.7385856	441.4335241	500.0647178	515.6824552	195.5363482	245.2569383	251.4224702
MAPK8IP2	2.091031967	0.305733871	6.839386036	7.95E-12	2.43E-10	Yes	OK	39.32501976	72.24473123	6.405308301	43.77853132	97.12000718	75.83565518	3.342501679	11.73478174	4.138641485
PAWR	0.728742983	0.106564095	6.838541483	8.00E-12	2.44E-10	Yes	OK	2710.449647	3400.840942	2020.058352	3469.448607	3500.453025	3232.621195	2152.571081	1737.921175	2169.682799
ATXN1	0.642563254	0.093964623	6.83835295	8.01E-12	2.44E-10	Yes	OK	1690.823153	2069.185503	1312.460802	2023.845021	2018.85632	2164.85517	1241.739374	1323.68338	1371.959652
TAF5	0.780134582	0.114101833	6.837178358	8.08E-12	2.45E-10	Yes	OK	1161.68824	1478.480747	844.8957324	1440.13127	1672.737145	1322.573826	823.0910385	833.1695034	878.4266553
RAI14	0.714400646	0.104560094	6.832440773	8.35E-12	2.53E-10	Yes	OK	4611.434786	5761.803702	3461.065869	6318.701354	5252.74592	5713.963832	3624.107445	3093.288466	3665.801696
EPAS1	0.785511675	0.114980887	6.831671727	8.39E-12	2.53E-10	Yes	OK	1011.303052	1288.467435	734.1386687	1269.577408	1384.476698	1211.348199	684.3772188	823.781678	694.2571092

KO_vs_Control_Differential_Expr

Gene Symbol	Log2 Fold Change	LFC Standard Err	Wald Statistic	p Value	FDR Adj p Va	Significa	Status	Base Mean	KD	Control	siICAM1_1	siICAM1_2	siICAM1_3	siCon_1	siCon_2	siCon_3
RSL1D1	0.625328927	0.0915406	6.831164805	8.42E-12	2.54E-10	Yes	OK	3665.058784	4463.981426	2866.136143	4742.674226	4404.495645	4244.774406	2927.195845	2669.662845	3001.549737
C21orf58	-1.109590252	0.162448163	-6.830426586	8.47E-12	2.55E-10	Yes	OK	257.2170204	156.4218984	358.0121424	136.8079104	179.7753324	152.6824524	325.8939137	394.2886664	353.853847
USP53	0.746260153	0.109300947	6.827572608	8.64E-12	2.59E-10	Yes	OK	1536.97978	1938.056483	1135.903078	2135.115455	1872.143117	1806.910877	1245.917501	1087.814267	1073.977465
HIST1H2BL	-1.045065141	0.153160772	-6.823321186	8.90E-12	2.66E-10	Yes	OK	679.033893	429.040663	929.027123	392.1826764	524.8613154	370.0779973	921.694838	1074.906007	790.4805237
FNDC3A	0.58603401	0.085922054	6.82053073	9.07E-12	2.71E-10	Yes	OK	2553.433985	3074.21628	2032.651689	3105.539566	3113.006188	3004.103087	2010.51476	2129.862885	1957.577423
IL10RB	-0.83847025	0.122938722	-6.820229106	9.09E-12	2.71E-10	Yes	OK	571.8064775	404.1847332	739.4282219	381.2380436	386.4136456	444.9025104	787.1591454	727.5564678	703.5690525
TMX4	-0.753387703	0.110471855	-6.819725264	9.12E-12	2.71E-10	Yes	OK	730.4272798	538.1473383	922.7072214	539.9352196	552.7574877	521.7493076	945.9279752	963.4255807	858.7681082
GGCT	0.675572448	0.099137807	6.814478403	9.46E-12	2.81E-10	Yes	OK	1506.478186	1861.620383	1151.33599	1925.343325	1951.698868	1707.818955	1158.176832	1110.110352	1185.720786
SGMS2	0.772920273	0.113429867	6.814080753	9.49E-12	2.81E-10	Yes	OK	2703.47838	3434.208535	1972.748226	3521.435613	3812.476878	2968.713115	2119.98169	2003.127243	1795.135744
DLC1	0.570552901	0.083764406	6.811400312	9.67E-12	2.86E-10	Yes	OK	3197.752116	3832.87724	2562.626993	3702.022055	4012.916041	3783.693622	2520.246266	2610.988937	2556.645778
CEP19	-1.135147228	0.166713826	-6.808956727	9.83E-12	2.90E-10	Yes	OK	217.3511311	130.1089323	304.5933298	133.1596994	110.5514975	146.6156	323.3870374	302.7573688	287.6355832
BMP4	-0.955745925	0.140476953	-6.803578118	1.02E-11	3.00E-10	Yes	OK	1918.888288	1274.383277	2563.393299	1171.075713	1035.257949	1616.816168	2405.765583	2706.040669	2578.373645
BIRC2	-0.649163456	0.095416003	-6.803507116	1.02E-11	3.00E-10	Yes	OK	3897.351099	3015.549293	4779.152905	3152.966308	2972.492135	2921.189438	5274.46765	4577.738356	4485.25271
TWF1	-0.925373422	0.136141629	-6.797137858	1.07E-11	3.13E-10	Yes	OK	810.4116543	547.5197386	1073.30357	543.5834306	609.5830238	489.3927614	1262.630009	1003.323839	953.9568624
BAK1	0.725814832	0.106868851	6.791640626	1.11E-11	3.24E-10	Yes	OK	1156.760441	1450.644476	862.8764056	1462.020536	1377.244357	1512.668535	949.2704768	808.5264618	830.8322782
FBXW7	0.700989678	0.103309018	6.78536774	1.16E-11	3.38E-10	Yes	OK	1128.313904	1404.597292	852.0305155	1423.714321	1437.169468	1352.908088	843.1460485	792.0977673	920.8477305
STK4	0.574054432	0.084730879	6.775032167	1.24E-11	3.62E-10	Yes	OK	3689.553082	4427.446645	2951.65952	4629.579687	4331.139044	4321.621203	3022.457143	2817.521095	3015.000322
AIG1	-1.075900323	0.158821475	-6.774274836	1.25E-11	3.64E-10	Yes	OK	470.7651823	292.1177209	649.4126437	310.0979302	237.6340601	328.6211724	743.7066236	535.1060473	669.4252603
SKP2	0.69126149	0.10212991	6.76845294	1.30E-11	3.78E-10	Yes	OK	1946.4037	2416.761114	1476.046286	2450.685701	2544.750826	2254.846814	1581.83892	1337.765118	1508.534821
SPAG4	-1.309594662	0.193502483	-6.76784424	1.31E-11	3.78E-10	Yes	OK	171.2197077	91.07591337	251.3635021	88.46911538	81.6221337	103.136491	249.8520005	300.4104125	203.8280932
CHSY1	-0.814017644	0.120626419	-6.748253427	1.50E-11	4.32E-10	Yes	OK	1037.207236	741.8512055	1332.563266	778.8930364	697.4043069	749.2562732	1455.659481	1135.926872	1406.103445
MIR711	-1.73725187	0.257665138	-6.742285291	1.56E-11	4.50E-10	Yes	OK	3562.887985	1272.3457	5853.430271	872.8344682	1646.907356	1297.295275	4649.419836	7455.106838	5455.764138
COL7A1	-1.73725187	0.257665138	-6.742285291	1.56E-11	4.50E-10	Yes	OK	3562.887985	1272.3457	5853.430271	872.8344682	1646.907356	1297.295275	4649.419836	7455.106838	5455.764138
DAAM1	0.795329623	0.11800591	6.739744009	1.59E-11	4.57E-10	Yes	OK	1311.654246	1676.168375	947.1401166	1576.93918	1825.649497	1625.916447	860.6941824	1087.814267	892.9119005
SSR3	0.777840615	0.115503056	6.734372604	1.65E-11	4.73E-10	Yes	OK	8386.106169	10669.4435	6102.768841	11056.81532	11543.84936	9407.665811	6971.622877	5317.029606	6019.65404
WWC1	0.99648422	0.148247358	6.721767122	1.80E-11	5.14E-10	Yes	OK	2505.333883	3384.465333	1626.202432	3269.709058	4114.168815	2769.518127	1493.262625	1976.137245	1409.207426
ITPR2	0.72743996	0.108238168	6.720734218	1.81E-11	5.17E-10	Yes	OK	1203.284703	1509.254145	897.3152602	1551.401704	1637.608632	1338.752099	904.9823296	893.0168903	893.9465608
SS18	-0.6103167	0.09084832	-6.717974505	1.84E-11	5.26E-10	Yes	OK	2304.597217	1814.551959	2794.642476	1788.535415	1935.167803	1719.952659	2888.757076	2747.112405	2748.057946
ZSCAN18	-0.991463573	0.147804419	-6.707942676	1.97E-11	5.62E-10	Yes	OK	483.4966488	314.6162822	652.3770153	307.361772	259.331083	377.1559918	650.1165766	605.5147377	701.4997318
ANKRD28	-0.767350506	0.114455293	-6.704368917	2.02E-11	5.75E-10	Yes	OK	4772.457736	3491.151724	6053.763749	3277.917533	4133.799455	3061.738185	6050.763664	6330.914748	5779.612834
LACTB	0.670708577	0.100122926	6.698851122	2.10E-11	5.96E-10	Yes	OK	1430.682968	1765.905016	1095.460921	1877.916583	1758.492045	1661.306419	1159.012457	1085.467311	1041.902994
PHF14	0.701967581	0.104821641	6.696781093	2.13E-11	6.03E-10	Yes	OK	1583.723266	1971.563789	1195.883463	2081.304343	1902.105673	1931.281352	1082.134919	1194.600781	1310.91469
RPL39L	-0.869778019	0.129976032	-6.6918339	2.20E-11	6.23E-10	Yes	OK	442.5428725	307.4503566	577.6353884	312.8340884	274.8289565	334.6880249	596.6365497	570.3103925	565.9592231
F2R	-0.747125179	0.111790084	-6.683286655	2.34E-11	6.59E-10	Yes	OK	696.3207259	514.384348	878.2571037	495.2446356	538.2928058	509.6156028	889.1054466	920.0068883	825.6589763
POLR1C	0.637024146	0.095380564	6.678762653	2.41E-11	6.78E-10	Yes	OK	1415.009977	1729.738682	1100.281272	1762.997938	1775.02311	1651.194999	1148.149327	1098.375571	1054.318918
TRIM47	-0.888280283	0.13302344	-6.677622231	2.43E-11	6.82E-10	Yes	OK	581.2648938	399.9620282	762.5677594	356.6126197	378.1481131	465.1253518	722.8159881	747.5055967	817.3816934
ITGA5	0.627179761	0.09401468	6.671083295	2.54E-11	7.12E-10	Yes	OK	5435.819072	6625.932699	4245.705444	6586.844858	6609.326446	6681.626792	4498.171635	3769.211894	4469.732804
CAPN15	0.987424127	0.14809874	6.66733643	2.60E-11	7.29E-10	Yes	OK	1437.081386	1936.714325	937.4484464	1672.704718	2314.349107	1823.089151	759.5835066	1119.498178	933.263655
GAPDH	-0.574759151	0.086276991	-6.66178948	2.71E-11	7.55E-10	Yes	OK	37145.34474	29705.69303	44584.99645	28844.57982	31192.05337	29080.44591	47157.68494	45519.21836	41078.08606
PDHX	0.736531089	0.110586074	6.660251739	2.73E-11	7.62E-10	Yes	OK	1722.264647	2166.326582	1278.202712	2089.512818	2457.962735	1951.504193	1348.699427	1265.009471	1220.899238
DUSP22	0.714586531	0.107312806	6.658911993	2.76E-11	7.67E-10	Yes	OK	847.5460312	1058.832355	636.2597073	1049.772699	1103.448592	1023.275774	594.1296734	670.0560372	644.5934113
E2F7	0.772734657	0.116070565	6.657455834	2.79E-11	7.73E-10	Yes	OK	4651.98116	5910.92463	3393.03769	6276.746928	5107.06591	6348.961052	3438.598602	2971.246736	3769.267733
ADIRF	-1.031014071	0.154893428	-6.656280283	2.81E-11	7.78E-10	Yes	OK	231.7447056	147.3134029	316.1760084	140.4561213	149.812777	151.6713104	311.6882816	336.7882359	300.0515077
ATP9A	-1.300344652	0.195470403	-6.652386402	2.88E-11	7.97E-10	Yes	OK	157.7487559	84.40663488	231.090877	62.01958604	99.18639031	92.01392828	220.6051108	234.6956348	237.9718854
CLCN5	0.910963217	0.136949605	6.651813409	2.90E-11	7.97E-10	Yes	OK	1002.41727	1323.879172	680.9553671	1433.746901	1180.93796	1356.952657	690.2265967	553.881698	798.7578067
ATP8B1	0.921139992	0.138483241	6.651635134	2.90E-11	7.97E-10	Yes	OK	1609.570255	2131.576169	1087.564341	2516.353498	1783.288643	2095.086367	1184.08122	909.4455847	1169.16622
DNAJB12	-0.645542653	0.097052273	-6.651494446	2.90E-11	7.97E-10	Yes	OK	1214.865667	940.9577864	1488.773547	966.7759	883.3787887	972.7186704	1477.385742	1511.439888	1477.49501
ZMYM3	-0.737413632	0.111022655	-6.642010417	3.09E-11	8.49E-10	Yes	OK	959.4210678	711.7927043	1207.049431	653.9418116	716.0017551	765.4345463	1118.066812	1261.489037	1241.592446
HNRNPH2	-0.756608666	0.113930955	-6.640940286	3.12E-11	8.53E-10	Yes	OK	1568.900239	1153.756316	1984.044162	1198.437295	1084.851144	1177.98051	2278.75052	1865.830296	1807.551669
R3HDM2	-1.210484941	0.182295964	-6.640217986	3.13E-11	8.56E-10	Yes	OK	195.6749768	111.3430316	280.0069221	92.11732632	106.4187313	135.4930373	265.7288835	262.8591109	311.4327718
GYG2	-1.6034148	0.241868345	-6.629287517	3.37E-11	9.20E-10	Yes	OK	74.97609624	30.8319831	119.1202094	21.88926566	39.2612795	31.34540414	121.1656859	116.1743392	120.0206031
DCP2	0.894769737	0.135196486	6.618291389	3.63E-11	9.89E-10	Yes	OK	3166.057473	4162.806154	2169.308793	3714.790793	5209.351875	3564.275793	2379.02557	2043.025501	2085.875309

Supplementary Table 3. Mass Spec Analysis of ICAM1-Regualted Proteins (related to Fig. 3)

#	Identified Proteins (2282/2286)	Accession I	Alternate ID	Molecular	siCON1	siCON2	siCON 3	siICAM1	siICAM2	siICAM3	Fold(con/si	Ttest 2-side
1776	Exosome complex component RRP45 OS=Homo sapiens OX=9606 GN=EXOSC9 PI	EXOS9_HU	EXOSC9	49 kDa		3	2	0	0		#DIV/0!	0.0377
1252	Pinin OS=Homo sapiens OX=9606 GN=PNN PE=1 SV=5	PININ_HUI	PNN	82 kDa	1	2	3	0	0	0	#DIV/0!	0.0257
717	DNA replication licensing factor MCM3 OS=Homo sapiens OX=9606 GN=MCM3 F	MCM3_HU	MCM3	91 kDa	10	16	19		1	1	15.00	0.0263
1216	Anillin OS=Homo sapiens OX=9606 GN=ANLN PE=1 SV=2	ANLN_HUI	ANLN	124 kDa	4	7	6		0	1	11.33	0.0230
1219	Condensin complex subunit 1 OS=Homo sapiens OX=9606 GN=NCAPD2 PE=1 SV=	CND1_HUI	NCAPD2	157 kDa	7	6	4		1	0	11.33	0.0230
809	UTP--glucose-1-phosphate uridylyltransferase OS=Homo sapiens OX=9606 GN=U	UGPA_HUI	UGP2	57 kDa	7	7	6	2	0	0	10.00	0.0013
1259	Cluster of Metastasis-associated protein MTA2 OS=Homo sapiens OX=9606 GN=I	MTA2_HUI	MTA2	75 kDa	5	6	6	1	0	1	8.50	0.0004
1095	Major prion protein OS=Homo sapiens OX=9606 GN=PRNP PE=1 SV=1	PRIO_HUM	PRNP	28 kDa	8	4	4	0	2	0	8.00	0.0352
1261	Nuclear cap-binding protein subunit 1 OS=Homo sapiens OX=9606 GN=NCBP1 PI	NCBP1_HU	NCBP1	92 kDa	7	8	4	1	1	1	6.33	0.0114
690	Protein transport protein Sec23A OS=Homo sapiens OX=9606 GN=SEC23A PE=1 S	SC23A_HU	SEC23A	86 kDa	22	17	16	4	4	1	6.11	0.0019
1331	SUMO-activating enzyme subunit 2 OS=Homo sapiens OX=9606 GN=UBA2 PE=1 S	SAE2_HUM	UBA2	71 kDa	5	3	4	1	1	0	6.00	0.0075
1562	Histone-arginine methyltransferase CARM1 OS=Homo sapiens OX=9606 GN=CAF	CARM1_HU	CARM1	66 kDa	3	3	2	1	0		5.33	0.0319
1033	N(G),N(G)-dimethylarginine dimethylaminohydrolase 2 OS=Homo sapiens OX=96	DDAH2_HU	DDAH2	30 kDa	5	7	4	1	1	1	5.33	0.0080
1559	Unconventional myosin-Ib OS=Homo sapiens OX=9606 GN=MYO1B PE=1 SV=3	MYO1B_HU	MYO1B	132 kDa	2	3	3	0	1		5.33	0.0319
1403	Cleavage stimulation factor subunit 1 OS=Homo sapiens OX=9606 GN=CSTF1 PE=	CSTF1_HUI	CSTF1	48 kDa		3	3	0	1	1	4.50	0.0123
1289	Zinc finger Ran-binding domain-containing protein 2 OS=Homo sapiens OX=9606	ZRAB2_HU	ZRANB2	37 kDa	4	4	5	0	2	1	4.33	0.0075
1242	Protein phosphatase 1F OS=Homo sapiens OX=9606 GN=PPM1F PE=1 SV=3	PPM1F_HU	PPM1F	50 kDa	6	4	7	1	1	2	4.25	0.0101
917	NAD(P) transhydrogenase, mitochondrial OS=Homo sapiens OX=9606 GN=NNT P	NNTM_HU	NNT	114 kDa	9	10	6	3	1	2	4.17	0.0090
571	Cluster of Leucine-rich repeat flightless-interacting protein 1 OS=Homo sapiens (	LRRF1_HUI	LRRFIP1	89 kDa	10	13	12	4	2	3	3.89	0.0012
997	Adenine phosphoribosyltransferase OS=Homo sapiens OX=9606 GN=APRT PE=1	APT_HUM/	APRT	20 kDa	10	8	4	2	3	1	3.67	0.0453
1305	Lymphokine-activated killer T-cell-originated protein kinase OS=Homo sapiens O	TOPK_HUN	PBK	36 kDa	4	5	5	2	1	1	3.50	0.0021
228	Cluster of Microtubule-associated protein 1B OS=Homo sapiens OX=9606 GN=M	MAP1B_HU	MAP1B	271 kDa	25	36	40	10	9	11	3.37	0.0064
1458	Thymidylate synthase OS=Homo sapiens OX=9606 GN=TYMS PE=1 SV=3	TYSY_HUM	TYMS	36 kDa	4	5	4	2	1	1	3.25	0.0031
1353	Protein KRI1 homolog OS=Homo sapiens OX=9606 GN=KRI1 PE=1 SV=3	KRI1_HUM	KRI1	83 kDa		5	7	3	2	1	3.00	0.0321
987	Structural maintenance of chromosomes protein 4 OS=Homo sapiens OX=9606 (	SMC4_HUI	SMC4	147 kDa	8	7	6	3	2	2	3.00	0.0022
901	1,4-alpha-glucan-branching enzyme OS=Homo sapiens OX=9606 GN=GBE1 PE=1	GLGB_HUN	**GBE1**	80 kDa	8	5	4	2	2	2	2.83	0.0380
1188	Cleavage stimulation factor subunit 3 OS=Homo sapiens OX=9606 GN=CSTF3 PE=	CSTF3_HUI	CSTF3	83 kDa	6	5	6	2	0	4	2.83	0.0380
1153	Protein mago nashi homolog 2 OS=Homo sapiens OX=9606 GN=MAGOHB PE=1 S	MGN2_HU	MAGOHB	17 kDa	6	4	4	1	1	3	2.80	0.0335
1455	Kinesin-like protein KIF11 OS=Homo sapiens OX=9606 GN=KIF11 PE=1 SV=2	KIF11_HUN	KIF11	119 kDa	4	4	4	2	1		2.67	0.0068
1332	ATP-dependent RNA helicase DDX18 OS=Homo sapiens OX=9606 GN=DDX18 PE=	DDX18_HU	DDX18	75 kDa	5	5	3	2	2	1	2.60	0.0232
1073	Glycerol-3-phosphate dehydrogenase, mitochondrial OS=Homo sapiens OX=960(	GPDM_HU	GPD2	81 kDa	7	7	4	2	2	3	2.57	0.0254
655	UV excision repair protein RAD23 homolog B OS=Homo sapiens OX=9606 GN=RA	RD23B_HU	RAD23B	43 kDa	17	9	15	6	6	4	2.56	0.0288
1265	Adenylate kinase isoenzyme 6 OS=Homo sapiens OX=9606 GN=AK6 PE=1 SV=1	KAD6_HUN	AK6	20 kDa	5	6	4	2	2	2	2.50	0.0065
961	Prolyl 4-hydroxylase subunit alpha-1 OS=Homo sapiens OX=9606 GN=P4HA1 PE=	P4HA1_HU	P4HA1	61 kDa	10	10	10	6	2	4	2.50	0.0065
1170	Transcription elongation factor A protein 1 OS=Homo sapiens OX=9606 GN=TCE/	TCEA1_HU	TCEA1	34 kDa	4	5	6	3	1	2	2.50	0.0213
1066	Cluster of DNA replication licensing factor MCM4 OS=Homo sapiens OX=9606 GN	MCM4_HU	MCM4	97 kDa	8	11	8	3	4	4	2.45	0.0072
1129	Exosome complex component RRP4 OS=Homo sapiens OX=9606 GN=EXOSC2 PE=	EXOS2_HU	EXOSC2	33 kDa	8	7	7	3	3	3	2.44	0.0002
1408	Omega-amidase NIT2 OS=Homo sapiens OX=9606 GN=NIT2 PE=1 SV=1	NIT2_HUM	NIT2	31 kDa	4	3	4	1	2		2.44	0.0319
830	SUMO-activating enzyme subunit 1 OS=Homo sapiens OX=9606 GN=SAE1 PE=1 S	SAE1_HUM	SAE1	38 kDa	8	8	8	2	4	4	2.40	0.0022
947	Tyrosine-protein kinase receptor UFO OS=Homo sapiens OX=9606 GN=AXL PE=1	UFO_HUM	AXL	98 kDa	6	11	7	4	2	4	2.40	0.0488
395	Alanine--tRNA ligase, cytoplasmic OS=Homo sapiens OX=9606 GN=AARS PE=1 SV	SYAC_HUM	AARS	107 kDa	21	17	18	9	4	11	2.33	0.0114
1093	S-adenosylmethionine synthase isoform type-2 OS=Homo sapiens OX=9606 GN=	METK2_HU	MAT2A	44 kDa	4	6	4	2	2	2	2.33	0.0161
1030	ATP synthase subunit O, mitochondrial OS=Homo sapiens OX=9606 GN=ATP5O F	ATPO_HUN	ATP5O	23 kDa	5	4	7	2	2	3	2.29	0.0335
814	Cytoskeleton-associated protein 5 OS=Homo sapiens OX=9606 GN=CKAP5 PE=1 S	CKAP5_HU	CKAP5	226 kDa	5	8	5	2	3	3	2.25	0.0341
1506	Deoxycytidylate deaminase OS=Homo sapiens OX=9606 GN=DCTD PE=1 SV=2	DCTD_HUN	DCTD	20 kDa	3	3	3	2	1	1	2.25	0.0075
926	Chromodomain-helicase-DNA-binding protein 4 OS=Homo sapiens OX=9606 GN=	CHD4_HUN	CHD4	218 kDa	7	7	6	3	2	4	2.22	0.0053
860	tRNA (cytosine(34)-C(5))-methyltransferase OS=Homo sapiens OX=9606 GN=NSU	NSUN2_HU	NSUN2	86 kDa	7	6	7	2	5	2	2.22	0.0254
1421	Sphingolipid delta(4)-desaturase DES1 OS=Homo sapiens OX=9606 GN=DEGS1 PI	DEGS1_HU	DEGS1	38 kDa	4	3	4	2	2	1	2.20	0.0132
1205	pre-rRNA processing protein FTSJ3 OS=Homo sapiens OX=9606 GN=FTSJ3 PE=1 S	SPB1_HUM	FTSJ3	97 kDa	4	5	6	3	1	3	2.14	0.0390
1154	NHL repeat-containing protein 2 OS=Homo sapiens OX=9606 GN=NHLRC2 PE=1 S	NHLC2_HU	NHLRC2	79 kDa	5	6	6	4	2	2	2.13	0.0158

Mass Spec Analysis of ICAM1-Regualted Proteins

#	Identified Proteins (2282/2286)	Accession I	Alternate ID	Molecular	siCON1	siCON2	siCON 3	siICAM1	siICAM2	siICAM3	Fold(con/si	Ttest 2-side
1206	U3 small nucleolar RNA-interacting protein 2 OS=Homo sapiens OX=9606 GN=RR	U3IP2_HUI	RRP9	52 kDa	6	7	6	4	3	2	2.11	0.0075
620	Probable ubiquitin carboxyl-terminal hydrolase FAF-X OS=Homo sapiens OX=960	USP9X_HU	USP9X	292 kDa	11	9	13	5	7	4	2.06	0.0175
1560	Actin-related protein 2/3 complex subunit 5-like protein OS=Homo sapiens OX=9	ARP5L_HU	ARPC5L	17 kDa	2	3	3	1	1	2	2.00	0.0474
1155	ATP-dependent 6-phosphofructokinase, liver type OS=Homo sapiens OX=9606 G	PFKAL_HUI	PFKL	85 kDa	9	9	8	6	2	5	2.00	0.0255
1696	Carbonyl reductase [NADPH] 3 OS=Homo sapiens OX=9606 GN=CBR3 PE=1 SV=3	CBR3_HUM	CBR3	31 kDa	6	10	8	4	4	4	2.00	0.0257
1376	Cleavage and polyadenylation specificity factor subunit 2 OS=Homo sapiens OX=	CPSF2_HUI	CPSF2	88 kDa	2	3	3	1	1	2	2.00	0.0474
1069	Cold-inducible RNA-binding protein OS=Homo sapiens OX=9606 GN=CIRBP PE=1	CIRBP_HUI	CIRBP	19 kDa	6	9	9	3	4	5	2.00	0.0257
1627	General transcription factor IIF subunit 1 OS=Homo sapiens OX=9606 GN=GTF2F	T2FA_HUM	GTF2F1	58 kDa	3	2	3	1	1	2	2.00	0.0474
1156	Phosphoserine aminotransferase OS=Homo sapiens OX=9606 GN=PSAT1 PE=1 S\	SERC_HUM	PSAT1	40 kDa	4	4	6	2	2	3	2.00	0.0352
618	Phosphoribosylformylglycinamidine synthase OS=Homo sapiens OX=9606 GN=PF	PUR4_HUM	PFAS	145 kDa	7	11	9	5	5	4	1.93	0.0226
1079	Small glutamine-rich tetratricopeptide repeat-containing protein alpha OS=Hom	SGTA_HUM	SGTA	34 kDa	5	6	6	4	2	3	1.89	0.0161
721	Cluster of Cullin-4B OS=Homo sapiens OX=9606 GN=CUL4B PE=1 SV=4 (CUL4B_H	CUL4B_HU	CUL4B	104 kDa	11	8	9	3	5	7	1.87	0.0406
707	Lon protease homolog, mitochondrial OS=Homo sapiens OX=9606 GN=LONP1 PE	LONM_HU	LONP1	106 kDa	8	12	8	5	6	4	1.87	0.0406
1282	Polyadenylate-binding protein-interacting protein 1 OS=Homo sapiens OX=9606	PAIP1_HUI	PAIP1	54 kDa	4	4	5	2	2	3	1.86	0.0132
1323	Small nuclear ribonucleoprotein E OS=Homo sapiens OX=9606 GN=SNRPE PE=1 S	RUXE_HUM	SNRPE	11 kDa	5	4	4	3	2	2	1.86	0.0132
1228	Structural maintenance of chromosomes protein 1A OS=Homo sapiens OX=9606	SMC1A_HU	SMC1A	143 kDa	4	3	4	2	2	2	1.83	0.0075
771	Deoxyuridine 5'-triphosphate nucleotidohydrolase, mitochondrial OS=Homo sap	DUT_HUM	DUT	27 kDa	10	10	11	4	7	6	1.82	0.0078
1130	Golgi resident protein GCP60 OS=Homo sapiens OX=9606 GN=ACBD3 PE=1 SV=4	GCP60_HU	ACBD3	61 kDa	6	8	6	3	3	5	1.82	0.0335
1023	Histone H1.0 OS=Homo sapiens OX=9606 GN=H1F0 PE=1 SV=3	H10_HUM	H1F0	21 kDa	5	6	7	3	4	3	1.80	0.0161
227	E3 ubiquitin-protein ligase HUWE1 OS=Homo sapiens OX=9606 GN=HUWE1 PE=	HUWE1_H	HUWE1	482 kDa	25	18	27	17	11	11	1.79	0.0379
142	Pre-mRNA-processing-splicing factor 8 OS=Homo sapiens OX=9606 GN=PRPF8 PE	PRP8_HUM	PRPF8	274 kDa	32	29	27	14	20	16	1.76	0.0052
646	Serpin B6 OS=Homo sapiens OX=9606 GN=SERPINB6 PE=1 SV=3	SPB6_HUM	SERPINB6	43 kDa	10	10	8	6	4	6	1.75	0.0132
855	Cluster of Hypoxanthine-guanine phosphoribosyltransferase OS=Homo sapiens (	HPRT_HUM	HPRT1	25 kDa	13	12	11	9	5	7	1.71	0.0179
1369	Peptidyl-prolyl cis-trans isomerase NIMA-interacting 4 OS=Homo sapiens OX=96	PIN4_HUM	PIN4	14 kDa	4	4	4	2	3	2	1.71	0.0075
1055	Serine/threonine-protein phosphatase 4 regulatory subunit 1 OS=Homo sapiens	PP4R1_HU	PPP4R1	107 kDa	6	6	5	3	3	4	1.70	0.0078
220	Galectin-1 OS=Homo sapiens OX=9606 GN=LGALS1 PE=1 SV=2	LEG1_HUM	LGALS1	15 kDa	25	29	24	14	17	15	1.70	0.0038
671	40S ribosomal protein S15a OS=Homo sapiens OX=9606 GN=RPS15A PE=1 SV=2	RS15A_HU	RPS15A	15 kDa	9	7	7	6	4	4	1.64	0.0335
302	Transcription intermediary factor 1-beta OS=Homo sapiens OX=9606 GN=TRIM2	TIF1B_HUM	TRIM28	89 kDa	25	22	25	12	16	16	1.64	0.0050
47	DNA-dependent protein kinase catalytic subunit OS=Homo sapiens OX=9606 GN	PRKDC_HU	PRKDC	469 kDa	75	56	49	35	37	39	1.62	0.0429
98	U5 small nuclear ribonucleoprotein 200 kDa helicase OS=Homo sapiens OX=960	U520_HUM	SNRNP200	245 kDa	39	45	44	30	26	23	1.62	0.0040
929	Double-strand break repair protein MRE11 OS=Homo sapiens OX=9606 GN=MRE	MRE11_HU	MRE11	81 kDa	7	8	6	4	4	5	1.62	0.0161
319	Splicing factor 3B subunit 2 OS=Homo sapiens OX=9606 GN=SF3B2 PE=1 SV=2	SF3B2_HUI	SF3B2	100 kDa	14	11	12	6	9	8	1.61	0.0201
15	Cluster of Glyceraldehyde-3-phosphate dehydrogenase OS=Homo sapiens OX=9	G3P_HUM	GAPDH	36 kDa	133	159	109	102	70	78	1.60	0.0440
434	Splicing factor 3A subunit 1 OS=Homo sapiens OX=9606 GN=SF3A1 PE=1 SV=1	SF3A1_HUI	SF3A1	89 kDa	12	12	12	8	7	8	1.57	0.0002
130	Non-POU domain-containing octamer-binding protein OS=Homo sapiens OX=96	NONO_HU	NONO	54 kDa	40	27	31	23	20	20	1.56	0.0425
77	Moesin OS=Homo sapiens OX=9606 GN=MSN PE=1 SV=3	MOES_HUI	MSN	68 kDa	55	46	41	37	25	30	1.54	0.0362
352	Serine/threonine-protein kinase PAK 2 OS=Homo sapiens OX=9606 GN=PAK2 PE	PAK2_HUM	PAK2	58 kDa	16	12	12	9	8	9	1.54	0.0274
878	Nuclear pore complex protein Nup160 OS=Homo sapiens OX=9606 GN=NUP160	NU160_HU	NUP160	162 kDa	9	8	9	6	6	5	1.53	0.0031
386	Delta-1-pyrroline-5-carboxylate synthase OS=Homo sapiens OX=9606 GN=ALDH	P5CS_HUM	ALDH18A1	87 kDa	15	16	16	14	8	9	1.52	0.0474
565	BAG family molecular chaperone regulator 3 OS=Homo sapiens OX=9606 GN=BA	BAG3_HUM	BAG3	62 kDa	9	10	11	7	6	7	1.50	0.0075
195	Cluster of Lamina-associated polypeptide 2, isoform alpha OS=Homo sapiens OX	LAP2A_HU	TMPO	75 kDa	29	36	29	20	17	26	1.49	0.0428
173	Thioredoxin reductase 1, cytoplasmic OS=Homo sapiens OX=9606 GN=TXNRD1 P	TRXR1_HU	TXNRD1	71 kDa	33	24	31	22	17	20	1.49	0.0353
69	Transketolase OS=Homo sapiens OX=9606 GN=TKT PE=1 SV=3	TKT_HUM	TKT	68 kDa	49	49	37	29	34	29	1.47	0.0297
786	Pyridoxal kinase OS=Homo sapiens OX=9606 GN=PDXK PE=1 SV=1	PDXK_HUM	PDXK	35 kDa	7	8	7	6	4	5	1.47	0.0249
920	40S ribosomal protein S21 OS=Homo sapiens OX=9606 GN=RPS21 PE=1 SV=1	RS21_HUM	RPS21	9 kDa	6	7	6	5	4	4	1.46	0.0132
549	Nuclear pore complex protein Nup155 OS=Homo sapiens OX=9606 GN=NUP155	NU155_HU	NUP155	155 kDa	13	13	12	9	9	8	1.46	0.0011
1159	Cluster of Golgi phosphoprotein 3 OS=Homo sapiens OX=9606 GN=GOLPH3 PE=1	GOLP3_HU	GOLPH3	34 kDa	5	6	5	4	3	4	1.45	0.0241
1274	E3 UFM1-protein ligase 1 OS=Homo sapiens OX=9606 GN=UFL1 PE=1 SV=2	UFL1_HUM	UFL1	90 kDa	5	4	4	3	3	3	1.44	0.0161
633	Protein NDRG1 OS=Homo sapiens OX=9606 GN=NDRG1 PE=1 SV=1	NDRG1_HU	NDRG1	43 kDa	12	11	10	8	9	6	1.43	0.0341
815	Cell growth-regulating nucleolar protein OS=Homo sapiens OX=9606 GN=LYAR P	LYAR_HUM	LYAR	44 kDa	7	7	6	5	5	4	1.43	0.0132

Mass Spec Analysis of ICAM1-Regualted Proteins

#	Identified Proteins (2282/2286)	Accession I	Alternate ID	Molecular	siCON1	siCON2	siCON 3	siICAM1	siICAM2	siICAM3	Fold(con/si	Ttest 2-side
413	Cluster of Histone H1.3 OS=Homo sapiens OX=9606 GN=HIST1H1D PE=1 SV=2 (H	H13_HUMA	HIST1H1D	22 kDa	11	11	8	7	7	7	1.43	0.0399
525	Proteasome subunit beta type-5 OS=Homo sapiens OX=9606 GN=PSMB5 PE=1 SV	PSB5_HUM	PSMB5	28 kDa	15	13	13	9	11	9	1.41	0.0132
842	Eukaryotic translation initiation factor 1 OS=Homo sapiens OX=9606 GN=EIF1 PE	EIF1_HUM	EIF1	13 kDa	8	8	8	5	7	5	1.41	0.0249
819	Replication protein A 32 kDa subunit OS=Homo sapiens OX=9606 GN=RPA2 PE=1	RFA2_HUM	RPA2	29 kDa	6	7	8	5	5	5	1.40	0.0257
326	Pre-mRNA-splicing factor ATP-dependent RNA helicase DHX15 OS=Homo sapiens	DHX15_HU	DHX15	91 kDa	15	15	17	12	11	11	1.38	0.0044
628	Cdc42-interacting protein 4 OS=Homo sapiens OX=9606 GN=TRIP10 PE=1 SV=3	CIP4_HUM	TRIP10	68 kDa	10	10	9	7	8	6	1.38	0.0161
564	Sodium/potassium-transporting ATPase subunit beta-1 OS=Homo sapiens OX=96	AT1B1_HU	ATP1B1	35 kDa	9	8	9	7	6	6	1.37	0.0078
197	Nuclear mitotic apparatus protein 1 OS=Homo sapiens OX=9606 GN=NUMA1 PE	NUMA1_H	NUMA1	238 kDa	18	21	21	13	15	16	1.36	0.0161
87	Cluster of Importin-5 OS=Homo sapiens OX=9606 GN=IPO5 PE=1 SV=4 (IPO5_HU	IPO5_HUM	IPO5	124 kDa	49	45	44	38	31	33	1.35	0.0097
236	Lamin-B2 OS=Homo sapiens OX=9606 GN=LMNB2 PE=1 SV=4	LMNB2_HU	LMNB2	70 kDa	20	18	20	16	13	14	1.35	0.0106
117	Programmed cell death 6-interacting protein OS=Homo sapiens OX=9606 GN=PD	PDC6I_HUI	PDCD6IP	96 kDa	36	37	37	27	28	27	1.34	0.0000
213	Cluster of ATP-dependent 6-phosphofructokinase, platelet type OS=Homo sapien	PFKAP_HU	PFKP	86 kDa	26	25	29	21	22	17	1.33	0.0265
581	Carbonyl reductase [NADPH] 1 OS=Homo sapiens OX=9606 GN=CBR1 PE=1 SV=3	CBR1_HUM	CBR1	30 kDa	16	18	18	15	11	13	1.33	0.0314
17	Cluster of Talin-1 OS=Homo sapiens OX=9606 GN=TLN1 PE=1 SV=3 (TLN1_HUMA	TLN1_HUM	TLN1	270 kDa	131	123	110	91	91	92	1.33	0.0081
216	Macrophage-capping protein OS=Homo sapiens OX=9606 GN=CAPG PE=1 SV=2	CAPG_HUM	CAPG	38 kDa	26	25	27	18	22	20	1.30	0.0097
71	ATP-dependent RNA helicase A OS=Homo sapiens OX=9606 GN=DHX9 PE=1 SV=4	DHX9_HUM	DHX9	141 kDa	47	43	39	35	29	36	1.29	0.0384
246	Calponin-3 OS=Homo sapiens OX=9606 GN=CNN3 PE=1 SV=1	CNN3_HUM	CNN3	36 kDa	23	20	22	16	17	18	1.27	0.0114
1048	Hydroxysteroid dehydrogenase-like protein 2 OS=Homo sapiens OX=9606 GN=H	HSDL2_HU	HSDL2	45 kDa	7	6	6	5	5	5	1.27	0.0161
185	Cluster of Polypyrimidine tract-binding protein 1 OS=Homo sapiens OX=9606 GN	PTBP1_HU	PTBP1	57 kDa	24	23	27	19	20	20	1.25	0.0160
264	Eukaryotic translation initiation factor 3 subunit M OS=Homo sapiens OX=9606 G	EIF3M_HU	EIF3M	43 kDa	23	24	24	19	19	19	1.25	0.0002
499	Ribosomal L1 domain-containing protein 1 OS=Homo sapiens OX=9606 GN=RSL1	RL1D1_HU	RSL1D1	55 kDa	12	11	13	9	10	10	1.24	0.0249
560	Gamma-enolase OS=Homo sapiens OX=9606 GN=ENO2 PE=1 SV=3	ENOG_HUI	ENO2	47 kDa	48	45	41	40	32	36	1.24	0.0478
251	Cluster of Calnexin OS=Homo sapiens OX=9606 GN=CANX PE=1 SV=2 (CALX_HUM	CALX_HUM	CANX	68 kDa	28	29	28	24	22	23	1.23	0.0013
44	Elongation factor 1-gamma OS=Homo sapiens OX=9606 GN=EEF1G PE=1 SV=3	EF1G_HUM	EEF1G	50 kDa	37	34	37	31	26	31	1.23	0.0265
5	Cluster of Heat shock protein HSP 90-beta OS=Homo sapiens OX=9606 GN=HSP9	HS90B_HU	HSP90AB1	83 kDa	310	264	274	232	225	241	1.21	0.0273
13	Cluster of Spectrin beta chain, non-erythrocytic 1 OS=Homo sapiens OX=9606 GN	SPTB2_HUI	SPTBN1	275 kDa	119	111	105	95	87	94	1.21	0.0146
32	Protein-glutamine gamma-glutamyltransferase 2 OS=Homo sapiens OX=9606 GN	TGM2_HUI	TGM2	77 kDa	44	45	49	39	37	38	1.21	0.0080
8	Cluster of 14-3-3 protein epsilon OS=Homo sapiens OX=9606 GN=YWHAE PE=1 S	1433E_HUI	YWHAE	29 kDa	183	175	187	145	152	156	1.20	0.0030
147	Coatomer subunit beta OS=Homo sapiens OX=9606 GN=COPB1 PE=1 SV=3	COPB_HUM	COPB1	107 kDa	28	28	28	23	23	24	1.20	0.0002
210	40S ribosomal protein S2 OS=Homo sapiens OX=9606 GN=RPS2 PE=1 SV=2	RS2_HUMA	RPS2	31 kDa	15	14	15	12	13	12	1.19	0.0078
609	Cluster of Scaffold attachment factor B1 OS=Homo sapiens OX=9606 GN=SAFB P	SAFB1_HU	SAFB	103 kDa	9	9	9	8	8	7	1.17	0.0161
605	Cytochrome c OS=Homo sapiens OX=9606 GN=CYCS PE=1 SV=2	CYC_HUMA	CYCS	12 kDa	10	9	10	8	8	9	1.16	0.0474
61	Cluster of Probable ATP-dependent RNA helicase DDX5 OS=Homo sapiens OX=96	DDX5_HUM	DDX5	69 kDa	65	62	64	54	57	54	1.16	0.0029
39	Cluster of Elongation factor 1-alpha 1 OS=Homo sapiens OX=9606 GN=EEF1A1 PE	EF1A1_HUI	EEF1A1	50 kDa	212	226	217	199	181	186	1.16	0.0117
657	Protein SEC13 homolog OS=Homo sapiens OX=9606 GN=SEC13 PE=1 SV=3	SEC13_HUI	SEC13	36 kDa	12	12	13	11	11	10	1.16	0.0241
125	4F2 cell-surface antigen heavy chain OS=Homo sapiens OX=9606 GN=SLC3A2 PE	4F2_HUMA	SLC3A2	68 kDa	29	26	26	24	24	23	1.14	0.0341
133	Coatomer subunit gamma-1 OS=Homo sapiens OX=9606 GN=COPG1 PE=1 SV=1	COPG1_HU	COPG1	98 kDa	38	40	40	34	34	36	1.13	0.0078
252	Caveolae-associated protein 1 OS=Homo sapiens OX=9606 GN=CAVIN1 PE=1 SV=	CAVN1_HU	CAVIN1	43 kDa	22	25	23	21	21	20	1.13	0.0474
375	Lysine--tRNA ligase OS=Homo sapiens OX=9606 GN=KARS PE=1 SV=3	SYK_HUMA	KARS	68 kDa	12	12	12	11	11	10	1.13	0.0161
321	Ras GTPase-activating protein-binding protein 1 OS=Homo sapiens OX=9606 GN	G3BP1_HU	G3BP1	52 kDa	18	19	19	17	16	17	1.12	0.0132
92	Glycine--tRNA ligase OS=Homo sapiens OX=9606 GN=GARS PE=1 SV=3	GARS_HUM	GARS	83 kDa	32	34	33	35	35	36	0.93	0.0249
129	Cathepsin D OS=Homo sapiens OX=9606 GN=CTSD PE=1 SV=1	CATD_HUM	CTSD	45 kDa	28	25	27	29	30	30	0.90	0.0335
16	60 kDa heat shock protein, mitochondrial OS=Homo sapiens OX=9606 GN=HSPD	CH60_HUM	HSPD1	61 kDa	102	105	107	124	116	112	0.89	0.0294
74	Stress-induced-phosphoprotein 1 OS=Homo sapiens OX=9606 GN=STIP1 PE=1 SV	STIP1_HUM	STIP1	63 kDa	24	25	23	27	27	29	0.87	0.0142
279	Integrin alpha-6 OS=Homo sapiens OX=9606 GN=ITGA6 PE=1 SV=5	ITA6_HUM	ITGA6	127 kDa	16	18	16	19	20	19	0.86	0.0232
701	Protein transport protein Sec23B OS=Homo sapiens OX=9606 GN=SEC23B PE=1 S	SC23B_HU	SEC23B	86 kDa	9	10	10	11	12	11	0.85	0.0241
25	Calreticulin OS=Homo sapiens OX=9606 GN=CALR PE=1 SV=1	CALR_HUM	CALR	48 kDa	51	56	61	73	66	70	0.80	0.0179
82	Integrin beta-1 OS=Homo sapiens OX=9606 GN=ITGB1 PE=1 SV=2	ITB1_HUM	ITGB1	88 kDa	33	30	27	36	38	41	0.78	0.0211
274	Succinate dehydrogenase [ubiquinone] flavoprotein subunit, mitochondrial OS=H	SDHA_HUM	SDHA	73 kDa	18	18	17	22	26	22	0.76	0.0146
1106	60S acidic ribosomal protein P1 OS=Homo sapiens OX=9606 GN=RPLP1 PE=1 SV=	RLA1_HUM	RPLP1	12 kDa	5	5	5	6	7	7	0.75	0.0075

Mass Spec Analysis of ICAM1-Regualted Proteins

#	Identified Proteins (2282/2286)	Accession I	Alternate ID	Molecular	siCON1	siCON2	siCON 3	siICAM1	siICAM2	siICAM3	Fold(con/si	Ttest 2-side
1087	Cofilin-2 OS=Homo sapiens OX=9606 GN=CFL2 PE=1 SV=1	COF2_HUM	CFL2	19 kDa	12	15	15	19	18	19	0.75	0.0114
342	LIM domain and actin-binding protein 1 OS=Homo sapiens OX=9606 GN=LIMA1 I	LIMA1_HU	LIMA1	85 kDa	11	14	10	16	17	15	0.73	0.0314
20	Cluster of Keratin, type I cytoskeletal 18 OS=Homo sapiens OX=9606 GN=KRT18 I	K1C18_HU	KRT18	48 kDa	106	81	101	134	129	132	0.73	0.0101
728	Protein PBDC1 OS=Homo sapiens OX=9606 GN=PBDC1 PE=1 SV=1	PBDC1_HU	PBDC1	26 kDa	6	7	7	9	9	10	0.71	0.0048
116	Major vault protein OS=Homo sapiens OX=9606 GN=MVP PE=1 SV=4	MVP_HUM	MVP	99 kDa	18	21	23	31	27	30	0.70	0.0101
542	Paraspeckle component 1 OS=Homo sapiens OX=9606 GN=PSPC1 PE=1 SV=1	PSPC1_HU	PSPC1	59 kDa	7	9	9	12	12	12	0.69	0.0053
1121	Protein transport protein Sec24C OS=Homo sapiens OX=9606 GN=SEC24C PE=1 S	SC24C_HU	SEC24C	118 kDa	3	4	3	5	5	5	0.67	0.0075
1393	Vitronectin OS=Homo sapiens OX=9606 GN=VTN PE=1 SV=1	VTNC_HUM	VTN	54 kDa	2	2	3	4	3	4	0.64	0.0474
493	Cluster of V-type proton ATPase subunit B, brain isoform OS=Homo sapiens OX=9	VATB2_HU	ATP6V1B2	57 kDa	7	11	14	19	17	16	0.62	0.0394
1223	Programmed cell death protein 10 OS=Homo sapiens OX=9606 GN=PDCD10 PE=1	PDC10_HU	PDCD10	25 kDa	2	2	2	3	3	4	0.60	0.0161
320	Zinc finger protein 185 OS=Homo sapiens OX=9606 GN=ZNF185 PE=1 SV=3	ZN185_HU	ZNF185	74 kDa	9	15	12	19	19	22	0.60	0.0161
941	Diablo homolog, mitochondrial OS=Homo sapiens OX=9606 GN=DIABLO PE=1 SV	DBLOH_HU	DIABLO	27 kDa	2	5	4	6	6	7	0.58	0.0474
858	Mitochondrial carrier homolog 2 OS=Homo sapiens OX=9606 GN=MTCH2 PE=1 S	MTCH2_HU	MTCH2	33 kDa	5	3	3	7	5	7	0.58	0.0474
783	Ferritin light chain OS=Homo sapiens OX=9606 GN=FTL PE=1 SV=2	FRIL_HUM	FTL	20 kDa	3	3	2	5	4	6	0.53	0.0249
761	Plasminogen activator inhibitor 1 OS=Homo sapiens OX=9606 GN=SERPINE1 PE=	PAI1_HUM	SERPINE1	45 kDa	2	4	6	8	8	7	0.52	0.0380
612	CD2-associated protein OS=Homo sapiens OX=9606 GN=CD2AP PE=1 SV=1	CD2AP_HU	CD2AP	71 kDa	4	6	3	8	8	9	0.52	0.0132
714	Glutathione synthetase OS=Homo sapiens OX=9606 GN=GSS PE=1 SV=1	GSHB_HUN	GSS	52 kDa	6	3	4	7	8	10	0.52	0.0327
1141	Thioredoxin domain-containing protein 12 OS=Homo sapiens OX=9606 GN=TXNI	TXD12_HU	TXNDC12	19 kDa	2	2	3	5	4	5	0.50	0.0078
998	45 kDa calcium-binding protein OS=Homo sapiens OX=9606 GN=SDF4 PE=1 SV=1	CAB45_HU	SDF4	42 kDa	4	3	5	8	8	9	0.48	0.0029
945	Nuclear receptor coactivator 4 OS=Homo sapiens OX=9606 GN=NCOA4 PE=1 SV=	NCOA4_HU	NCOA4	70 kDa	4	3	3	6	9	6	0.48	0.0254
1112	Desmoglein-2 OS=Homo sapiens OX=9606 GN=DSG2 PE=1 SV=2	DSG2_HUN	DSG2	122 kDa	1	3	4	6	5	6	0.47	0.0335
1342	Guanine nucleotide-binding protein G(I)/G(S)/G(O) subunit gamma-12 OS=Homo	GBG12_HU	GNG12	8 kDa	2	2	2	4	5	4	0.46	0.0022
1360	Trans-Golgi network integral membrane protein 2 OS=Homo sapiens OX=9606 G	TGON2_HU	TGOLN2	51 kDa	2	2	2	5	4	4	0.46	0.0022
1198	Cluster of Ribonucleoside-diphosphate reductase large subunit OS=Homo sapien	RIR1_HUM	RRM1	90 kDa	1	3	3	7	6	4	0.41	0.0394
1173	Tumor protein D52 OS=Homo sapiens OX=9606 GN=TPD52 PE=1 SV=2	TPD52_HU	TPD52	24 kDa	2	3	2	6	6	5	0.41	0.0021
986	Sideroflexin-1 OS=Homo sapiens OX=9606 GN=SFXN1 PE=1 SV=4	SFXN1_HU	SFXN1	36 kDa	2	3	4	6	10	6	0.41	0.0406
1235	Cluster of C-Jun-amino-terminal kinase-interacting protein 4 OS=Homo sapiens C	JIP4_HUM	SPAG9	146 kDa	1	2	2	6	4	3	0.38	0.0474
1062	Pseudouridine-5'-phosphatase OS=Homo sapiens OX=9606 GN=PUDP PE=1 SV=3	HDHD1_HU	PUDP	25 kDa	3	4	1	7	6	8	0.38	0.0147
1097	Septin-10 OS=Homo sapiens OX=9606 GN=SEPT10 PE=1 SV=2	SEP10_HUI	10-Sep	53 kDa	2	2	3	8	7	4	0.37	0.0327
1712	Acyl-CoA dehydrogenase family member 9, mitochondrial OS=Homo sapiens OX=	ACAD9_HU	ACAD9	69 kDa	0	1	1	2	2	2	0.33	0.0161
351	Protein CYR61 OS=Homo sapiens OX=9606 GN=CYR61 PE=1 SV=1	CYR61_HU	CYR61	42 kDa	7	3	8	18	20	17	0.33	0.0022
971	GrpE protein homolog 1, mitochondrial OS=Homo sapiens OX=9606 GN=GRPEL1	GRPE1_HU	GRPEL1	24 kDa	2	3	2	9	7	6	0.32	0.0061
1709	Vacuolar protein sorting-associated protein 4B OS=Homo sapiens OX=9606 GN=V	VPS4B_HU	VPS4B	49 kDa	1	1	0	2	3	2	0.29	0.0241
630	Integrin alpha-5 OS=Homo sapiens OX=9606 GN=ITGA5 PE=1 SV=2	ITA5_HUM	ITGA5	115 kDa	4	2	3	12	7	13	0.28	0.0169
1510	Hemoglobin subunit alpha OS=Homo sapiens OX=9606 GN=HBA1 PE=1 SV=2	HBA_HUM	HBA1	15 kDa	1	1	1	4	3	4	0.27	0.0013
1045	Guanine nucleotide-binding protein-like 3 OS=Homo sapiens OX=9606 GN=GNL3	GNL3_HUN	GNL3	62 kDa	1	1	3	4	9	6	0.26	0.0433
1540	Protein AATF OS=Homo sapiens OX=9606 GN=AATF PE=1 SV=1	AATF_HUN	AATF	63 kDa		1	0	3	2	2	0.21	0.0486

Supplementary Table 4: Stemness Genes

ACTA2	EMP1	NYNRIN
ADGRG1	EPHA4	OLR1
AKR1C3	FAM30A	POU5F1
AKT1	FGFR2	PROM1
ALDH1A1	FOXO3	PTCH1
ANRF3	FSCN1	RNF43
ARHGAP22	GAS1	RSPO1
AXIN2	GLI1	RSPO2
BCL2	GLI2	RTK2
BCL2L11	HES1	SANI2
BEX3	HIF2	SAV1
BIM	IGFBP6	SMAD3
BOC	ITGA6	SMIM24
CAV1	JAG1	SMO
CD24	JAG2	SNAI1
CD34	KLF4	SOCS2
CD44	KLF5	SOX2
CDH1	KRT14	SPARC
CDH2	KRT17	SPON1
CDH3	KRT5	STK3
CDK6	LAPTM4B	TAZ
CDON	LAT	TGM2
COL1A2	LRG5	TP53
CPXM1	MMRN1	TP63
CTNNB1	MST2	TSPAN8
CXCL8	MYC	TWIST1
DAND5	MYLK	VCAM1
DLL1	NANOG	VIM
DLL4	NFYA	YAP
DNMT3B	NOTCH2	YY1AP1
DPP4	NOTCH3	ZBTB46
DPYSL3	NOTCH4	ZEB1
EGFR	NTRK2	
