# Supplementary material for: ICAM1 initiates CTC cluster formation and trans-endothelial migration in lung metastasis of breast cancer
Source: Nat Commun. 2021 Aug 11;12:4867. doi: 10.1038/s41467-021-25189-z (PMC8358026; doi:10.1038/s41467-021-25189-z)
Supplement: Supplementary file 2 — Reporting Summary [file 41467_2021_25189_MOESM2_ESM.pdf]

nature research

Corresponding author(s): Huiping Liu

Last updated by author(s): May 25, 2021

Reporting Summary

Nature Research wishes to improve the reproducibility of the work that we publish. This form provides structure for consistency and transparency in reporting. For further information on Nature Research policies, see our Editorial Policies and the Editorial Policy Checklist.

Statistics

For all statistical analyses, confirm that the following items are present in the figure legend, table legend, main text, or Methods section.

n/a	Confirmed	
☐	☒	The exact sample size (*n*) for each experimental group/condition, given as a discrete number and unit of measurement
☐	☒	A statement on whether measurements were taken from distinct samples or whether the same sample was measured repeatedly
☐	☒	The statistical test(s) used AND whether they are one- or two-sided<br>*Only common tests should be described solely by name; describe more complex techniques in the Methods section.*
☐	☒	A description of all covariates tested
☒	☐	A description of any assumptions or corrections, such as tests of normality and adjustment for multiple comparisons
☐	☒	A full description of the statistical parameters including central tendency (e.g. means) or other basic estimates (e.g. regression coefficient) AND variation (e.g. standard deviation) or associated estimates of uncertainty (e.g. confidence intervals)
☐	☒	For null hypothesis testing, the test statistic (e.g. *F*, *t*, *r*) with confidence intervals, effect sizes, degrees of freedom and *P* value noted<br>*Give P values as exact values whenever suitable.*
☒	☐	For Bayesian analysis, information on the choice of priors and Markov chain Monte Carlo settings
☒	☐	For hierarchical and complex designs, identification of the appropriate level for tests and full reporting of outcomes
☐	☒	Estimates of effect sizes (e.g. Cohen's *d*, Pearson's *r*), indicating how they were calculated

Our web collection on statistics for biologists contains articles on many of the points above.

Software and code

Policy information about availability of computer code

Data collection	1. Affymetrix Human Genome U133A Array data for HER2-negative breast cancer cases treated pre-operatively with taxane-anthracycline chemotherapy were downloaded from Gene Expression Omnibus (GEO; GSE25055; N = 310). Similarly, gene expression data from breast cancer tissue in a large population-based cohort of Swedish patients was also downloaded from GEO (GSE1456-GPL96; N = 159).<br>2. Cultured cells were incubated and monitored by the IncuCyte live cell imaging system (Essen BioScience), and images were acquired every 2 h.<br>3. The monomeric structure of ICAM1's (UniProt ID: P05362) ExD (aa 28-477) was retrieved from the Protein Data Bank (PDB ID: 1Z7Z; Chain I).<br>4. Cells were diluted in PBS and filtered, prior to analysis on a BD-LSR II flow cytometer (BD Biosciences) or a BDAria cell sorter (BD Biosciences)
Data analysis	Please refer to the method sections for details.<br>1. For Gene set enrichment analysis, analysis was performed using a rank-ordered list of differentially expressed genes on the C2 Chemical and Genetic Perturbations curated gene set collection. Analysis was performed using GSEA software version 4.0.3 and MSigDB gene set collections version 7.1. Of note, inflammatory breast cancers were excluded from this analysis. The data were pre-processed with an RMA (Robust multichip averaging) algorithm using the R/Bioconductor package Oligo 69, where background subtraction, quantile normalization, and summarization (via median-polish) were accomplished. ICAM1 expression was obtained for each sample, and patients within each cohort were divided into two groups according to the median expression level of ICAM1. In addition, a 98-gene stemness signature (Supplementary Table 1) was evaluated on a per-sample basis using the single sample Gene Set Enrichment Analysis (ssGSEA) (Version 9) protocol, thus providing a Stemness Signature Index per patient tumor. Patients were then divided into groups based on whether their tumors express higher than median levels of ICAM1 expression either alone or in combination with the Stemness Signature Index. Survival differences between these groups were estimated using the Survival package in R and the Kaplan-Meier survival plots were created using the ggsurvplot command from the Survminer package (Version 0.4.9) in R (Version 3.7).<br>2. Cluster size of aggregated cells was analyzed over time by the IncuCyte ZOOM (2016A BID 1.0 2016-07-29) software.

3. The homodimer models were initially built with rigid docking by the ClusPro webserver (dimer mode) and then flexible refinement by BAL (Bayesian Active Learning), a machine learning-assisted protein docking method with uncertainty quantification. BAL predicted not only the refined dimer structure models but also the conditional probabilities of individual models (summing up to 1), interfacial residue-contacts, and interfacial residues.
4. Flow cytometry data and dot plots have been analyzed by BDAria v8.0.2 or Flow Jo v10.7.1.

For manuscripts utilizing custom algorithms or software that are central to the research but not yet described in published literature, software must be made available to editors and reviewers. We strongly encourage code deposition in a community repository (e.g. GitHub). See the Nature Research guidelines for submitting code & software for further information.

Data

Policy information about availability of data

All manuscripts must include a data availability statement. This statement should provide the following information, where applicable:

- Accession codes, unique identifiers, or web links for publicly available datasets
- A list of figures that have associated raw data
- A description of any restrictions on data availability

Affymetrix Human Genome U133A Array data for HER2-negative breast cancer cases treated pre-operatively with taxane-anthracycline chemotherapy were downloaded from Gene Expression Omnibus (GEO; GSE25055; N = 310). Similarly, gene expression data from breast cancer tissue in a large population-based cohort of Swedish patients was also downloaded from GEO (GSE1456-GPL96; N = 159).
All single-cell and bulk-cell RNA sequencing data that support the findings of this study have been deposited in the National Center for Biotechnology Information Sequence Read Archive (SRA) and are accessible through the SRA Series accession number PRJNA706068 (currently released). All other relevant data are available from the corresponding author on request.
The GSE25055 and GSE1456 data referenced during the study are available in a public repository from the Gene Expression Omnibus (https://www.ncbi.nlm.nih.gov/geo/) website.
The label-free mass spectrometry raw data files have been deposited to jPOST (https://repository.jpostdb.org/entry/JPST001184) with accession number JPST001184.0 and PXD026234 (publicly available).

Field-specific reporting

Please select the one below that is the best fit for your research. If you are not sure, read the appropriate sections before making your selection.

- [x] Life sciences
- [ ] Behavioural & social sciences
- [ ] Ecological, evolutionary & environmental sciences

For a reference copy of the document with all sections, see nature.com/documents/nr-reporting-summary-flat.pdf

Life sciences study design

All studies must disclose on these points even when the disclosure is negative.

Sample size	Affymetrix Human Genome U133A Array data for HER2-negative breast cancer cases treated pre-operatively with taxane-anthracycline chemotherapy were downloaded from Gene Expression Omnibus (GEO; GSE25055; N = 310). Similarly, gene expression data from breast cancer tissue in a large population-based cohort of Swedish patients was also downloaded from GEO (GSE1456-GPL96; N = 159). The other studies in the laboratory with biological replicates N>=3 and technical replicates n>=3.<br>We determined the sample size using Bonferroni correction for multiplicity, with $\alpha = 0.05/4 = 0.0125$, assuming that the mean difference between the groups will be at least twice the standard deviation (effect size = 2.0), to achieve 80% power using the two-sample t-test.
Data exclusions	no data exclusions.
Replication	All experiments were replicated at least three times. All attempts at replication were successful.
Randomization	Mouse groups were randomized. All other experiments were conducted with randomly allocated samples and groups.
Blinding	The data collection and analyses of mouse experiments and other experiments were blinded.

Reporting for specific materials, systems and methods

We require information from authors about some types of materials, experimental systems and methods used in many studies. Here, indicate whether each material, system or method listed is relevant to your study. If you are not sure if a list item applies to your research, read the appropriate section before selecting a response.

Materials & experimental systems

n/a	Involved in the study	
☐	☒	Antibodies
☐	☒	Eukaryotic cell lines
☒	☐	Palaeontology and archaeology
☐	☒	Animals and other organisms
☐	☒	Human research participants
☒	☐	Clinical data
☒	☐	Dual use research of concern

Methods

n/a	Involved in the study	
☒	☐	ChIP-seq
☐	☒	Flow cytometry
☒	☐	MRI-based neuroimaging

Antibodies

Antibodies used	The primary antibodies used include: ICAM1 (1:3000, Sigma Aldrich, HPA004877), CDK6 (1:1000, Protein Tech, 14052-1-AP), CD44 (1:1000, Thermo Fisher, 156-3C11), EpCAM (1:1000, Thermo Fisher, MA1-10195), Oct3/4 (1:1000, Santa Cruz, sc-5279), c-Myc (1:1000, Thermo Fisher, 13-2500), Flag (1:1000, Sigma-Aldrich, F7425), Sec23a (1:5000, Thermo Fisher, PA5-28984), HIF1a (1:2000, Thermofisher, MA1-516), BMP4 (1:500, Protein Tech, 12492-1-AP), HMGA2 (1:2000, Protein Tech, 20795-1-AP), CD34 (1:500, Protein Tech, 14486-1-AP), N Cadherin (1:4000, Protein Tech, 22018-1-AP), PAWR (1:500, Protein Tech, 20688-1-AP), PAI1 (1:500, Protein Tech, 13801-1-AP), KRT19 (1:2000, Protein Tech, 10712-1-AP), MCM3 (1:2000, Protein Tech, 15597-1-AP), ANKRD1 (1:3000, Protein Tech 11427-1-AP), Notch1 (1:1000, Cell signaling 3608S), and β-actin (1:1000, Abcam ab8224). Zeb1 antibody was a gift from Dr. Hidayatullah G. Munshi (Northwestern University). The horseradish peroxidase (HRP)-conjugated secondary antibodies used were from Promega (1:10,000, Rabbit W401B and Mouse W402B), and the substrate ECL was detected by Pierce ECL2 solution (Thermo Fisher Scientific, 1896433A). The full blot images of Western blots have been provided in a Supplementary document. Anti-ICAM1 neutralizing antibody (R&D Systems, AF720) was used in clustering assay, transendothelial migration assay and in vivo treatment. CellSearch kit and anti-ICAM1 antibody (conjugated to PE, BD# 555511) were used to enrich CTCs for immunofluorescence staining.
Validation	These antibodies are provided by commercial vendors with validation.

Eukaryotic cell lines

Policy information about cell lines

Cell line source(s)	MDA-MD-231, MCF7, BT549, MCF-10A, MCF-12A, SKBR3, BT-474, and HEK-293 were obtained from ATCC. FC-IBC-02 and EMF-01 were generated by and obtained from Dr. Massimo Cristofanilli's laboratory at Northwestern University.
Authentication	We have RNA sequencing, mass spectrometry and microarray data for MDA-MB-231, western blotting for MCF-10A, MCF-12A, SKBR3, BT-474, MDA-MB-231, MCF7, and HEK-293 cells, and flow cytometry analysis for MDA-MD-231, MCF7, BT549, FC-IBC-02, and EMF-01.
Mycoplasma contamination	No mycoplasma contanmination as measured routinely in the laboratory.
Commonly misidentified lines (See ICLAC register)	None.

Animals and other organisms

Policy information about studies involving animals; ARRIVE guidelines recommended for reporting animal research

Laboratory animals	Female NSG mice at age of 6-8 weeks Animals (Jackson Laboratory) were randomized by age and weight for human PDX inoculations or injections. B6 female mice were utilized to receive E0771 tumor cells. All mice used in this study were housed in specific pathogen-free facilities, with regular diet and regular light/dark cycles, and regular ambient temperature and humidity in the Animal Resources Facilities at Northwestern University.
Wild animals	no
Field-collected samples	no
Ethics oversight	All animal procedures conformed to the NIH Guidelines for the Care and Use of Laboratory Animals and were accepted by the Northwestern University Institutional Animal Care and Use Committees.

Note that full information on the approval of the study protocol must also be provided in the manuscript.

Human research participants

Policy information about studies involving human research participants

Population characteristics	Affymetrix Human Genome U133A Array data for HER2-negative breast cancer cases treated pre-operatively with taxane-anthracycline chemotherapy were downloaded from Gene Expression Omnibus (GEO; GSE25055; N = 310, age mean=51, SD=11, details see https://www.ncbi.nlm.nih.gov/pmc/articles/PMC5638042/). Similarly, gene expression data from breast

	cancer tissue in a large population-based cohort of Swedish patients was also downloaded from GEO (GSE1456-GPL96; N = 159). For CTC analysis, blood samples were collected from breast cancer patients at stage III-IV at Northwestern University hospitals.
Recruitment	For CTC analysis, breast cancer patients with stage III-IV diseases were recruited at Northwestern Memorial Hospital based on their availability and willingness to consent and participate in the research. The experimental design and data analyses were blinded so not affected.
Ethics oversight	The human specimen collection and blood sample analyses were approved by the Northwestern University Institutional Review Board following NIH guidelines for human subject studies. Written consent was obtained from all patients whose blood samples were analyzed for the study.

Note that full information on the approval of the study protocol must also be provided in the manuscript.

Flow Cytometry

Plots

Confirm that:

- [x] The axis labels state the marker and fluorochrome used (e.g. CD4-FITC).
- [x] The axis scales are clearly visible. Include numbers along axes only for bottom left plot of group (a 'group' is an analysis of identical markers).
- [ ] All plots are contour plots with outliers or pseudocolor plots.
- [x] A numerical value for number of cells or percentage (with statistics) is provided.

Methodology

Sample preparation	Live blood cell samples were centrifuged and after red blood cells were lysed, white blood cells were stained with antibodies for lineage markers such as CD45 (leukocytes), EpCAM (epithelial), and candidate markers ICAM1 and CD44 for flow cytometry analysis on FACS LSR (BD Biosciences). Single and clustered tumor cells were gated for ICAM1 expression (%). Dissociated PDX tumor cells or MDA-MB-231 cells were resuspended at 10 million per mL in PBS with 2% FBS. Cells were first blocked with IgG for 10 min, then incubated with mouse anti-human ICAM1-APC antibody (BD Biosciences #559771) for 25 min on ice, followed by washing twice with PBS. Finally, cells were diluted in PBS and filtered, prior to analysis on a BD-LSR II flow cytometer (BD Biosciences) or a BDAria cell sorter (BD Biosciences).
Instrument	BD FACSaria and LSR-II.
Software	BD FACSaria and flowJo.
Cell population abundance	ICAM1+ cells in TNBC are from 1-90%
Gating strategy	Gate Dapi negative viable cells are gated based on FSC and SSC for singles and clusters, and gated for cell populations.

- [x] Tick this box to confirm that a figure exemplifying the gating strategy is provided in the Supplementary Information.
