# Supplementary material for: ICAM1 initiates CTC cluster formation and trans-endothelial migration in lung metastasis of breast cancer
Source: Nat Commun. 2021 Aug 11;12:4867. doi: 10.1038/s41467-021-25189-z (PMC8358026; doi:10.1038/s41467-021-25189-z)

Figure 1 Source Data

Fig 1d

PDX type	Primary Tumor ICAM1 %	Lung Mets ICAM1 %
TN1	0.1	8
TN1	1.2	60.7
TN1	0.6	91.2
TN2	2.2	7.4
TN2	2.9	19.3
TN2	4	16.8
TN3	32	99
TN3	26.5	45.9
TN3	33.1	68.1
Average	Primary Tumor	Lung Metastasis
TN1	0.633333333	53.3
TN2	3.033333333	14.5
TN3	30.53333333	71
SD		
TN1	0.550757055	42.09073532
TN2	0.907377173	6.274551777
TN3	3.536005279	26.66852077

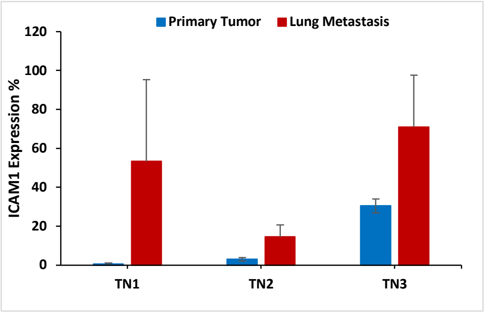

Fig 1j TN1- Lung mets BLI

Con	ICAM1 OE
1.363738158	8.156564851
3.435571341	11.77442128
1.537690274	10.15922578

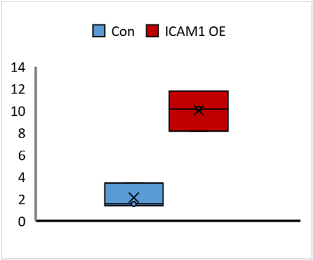

Fig 1m 231- Lung mets BLI

siCon	siICAM1
0.68	0.08
0.38	0.39
0.58	0.14
0.48	0.13

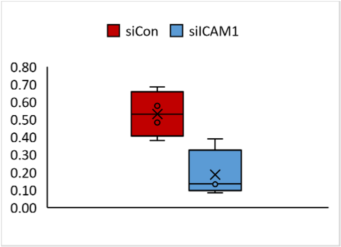

Figure 2 Source Data

Fig 2c BC Patient CTC data

% ICAM1+ CD45-

patient	Singles	Clusters
1	0.000000	0.000000
2	0	0
3	0	0
4	0.00058777	0.00099985
5	0.0002883	0.00035732
6	0.004016	0.000000
7	0.003009	0.000000
8	0.011194	0.000000
9	0.0108729	0.00760109
10	0.026432	0.000000
11	0.047619	0.000000
12	0.1	0.000000
13	0.10526316	0.000000
14	0.16666667	0.03703704
15	0.005131	0.007585
16	0.002165	0.009346
17	0.002705	0.010169
18	0.002869	0.017647
19	0.004383	0.018223
20	0.000643	0.019017
21	0.00730927	0.020906
22	0.00127138	0.02998362
23	0.013713	0.036900
24	0.033333	0.057143
25	0.03496503	0.05714286
26	0.01485934	0.1211422
27	0.03846154	0.125
28	0.03477748	0.137097
29	0.041342	0.184532
30	0.071749	0.205882
31	0.028369	0.208835
32	0.025907	0.219780
33	0.05882353	0.250000
34	0.009097	0.256745
35	0.009042	0.273552
36	0.055762	0.297975
37	0.046526	0.396721
38	0.004373	0.467532
39	0.01722575	0.500000
40	0.100000	0.500000
41	0.046358	0.558824
42	0.128931	0.632057
43	0.002346	0.660482
44	0	0.666667
45	0.114754	0.687500
46	0.1147541	0.6875
47	0.05825243	0.77777778
48	0.157895	0.835294
49	0.224806	1.000000
50	0.23529412	1
51	0.00650234	0.993056

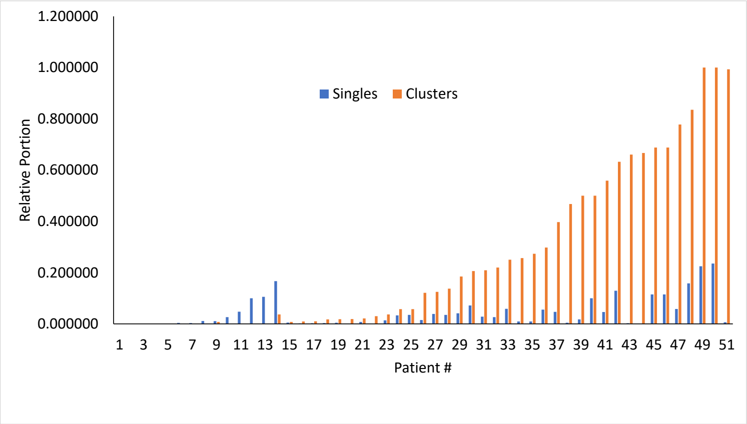

Fig 2d TN1 aggregation

Hours	ICAM1+	ICAM1-	ICAM1 + (Std Err)	ICAM1 - (Std Err)
0	430.925	324.4	55.39558	45.69983
1	902.35	614.925	77.57028	56.8914
4	1159.575	750.425	67.96844	56.64291
8	1272.275	859.95	64.40472	55.07319
12	1399.525	977.65	58.75548	59.1491
16	1506.4	1024.4	54.41523	52.46752
20	1622.7	1117.975	60.32451	34.88438
24	1678.975	1211.8	62.40241	38.16537

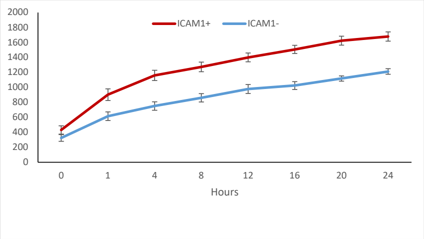

Fig 2f 231 aggregation

Hours	231 siCon	231 siICAM1	231 siCon (Std Dev)	231 siICAM1(Std Dev)
0	3909.489	4117.917	601.5955	406.3486
1	5493.189	5285.05	527.6917	832.7318
2	9626.51	8640.222	1500.192	1557.587
4	24901.68	12715.75	7290.848	2989.454
6	52061.35	14744.43	23281.75	2960.499
8	61409.54	14945.16	17149.39	2650.304
10	86659.21	14049.85	26067.09	2447.673
12	95195.88	12996.04	36073.21	1769.213
14	94552.31	11738.71	33233.36	1458.859
16	112093.3	12242.66	41117.04	1798.081
18	109234.3	11318.44	30983.1	1768.445
20	113271.4	10544.6	38052.67	1695.872

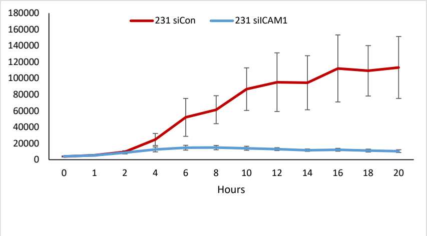

Fig 2i Solid phase self-interaction

BSA	ICAM1
0.202	0.651
0.202	0.65
0.269	0.605
0.269	0.605
	0.704
	0.703

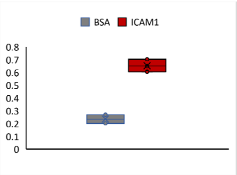

Figure 3 Source Data

Fig 3e

231 Mammosphere

siCon	67	73	77
siICAM1	39	42	34
siCDK6	38	53	46

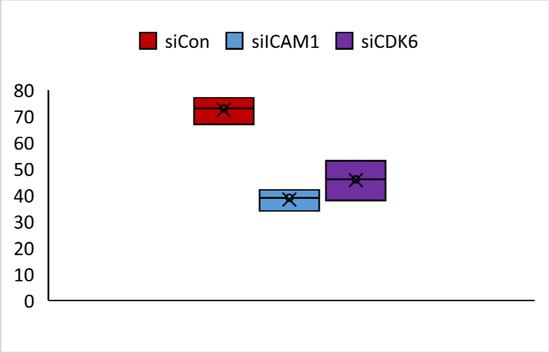

Fig 3h

231 BLI Lung Metastasis

siCon	6.08175166	2.78633303	5.4786357
siICAM1	0.7813712	0.48766357	0.62474464
siCDK6	2.34135703	2.11040463	1.17713264

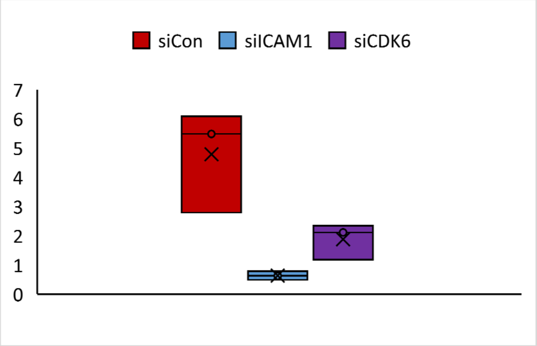

Fig 3j

231 Proliferation

siCon	siICAM1	siCDK6
75,000	55,000	30,000
60,000	35,000	60,000
110,000	35,000	55,000
85,000	50,000	55,000

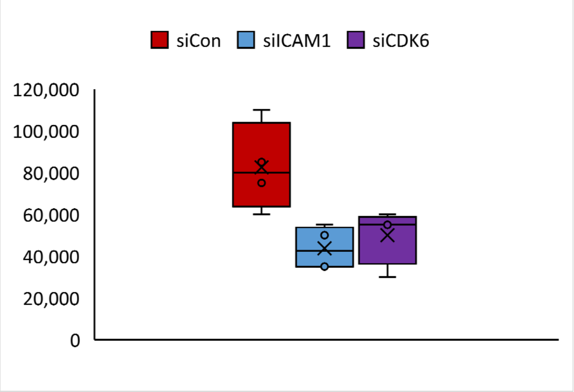

Figure 4 Source Data

Fig 4b TEM

	siCon	EC siICAM1	Tumor siICAM1	Tumor+EC siICAM1
Normalized value	1.371428571	0.829411765	0.428571429	0.084507042
	0.771428571	0.776470588	0.514285714	0.042253521
	0.857142857	0.952941176	0.385714286	0.042253521
	0.970588235			
	0.988235294			
	1.041176471			

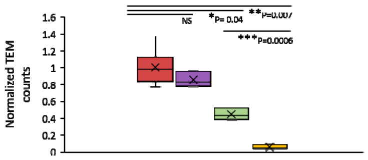

Fig 4d 231-HUVEC co-culture aggregation

hours	231 Con, HUVEC Con	231 siICAM1 , HUVEC siICAM1	231 Con, H Con (	231 siICAM1 , H siICAM1 (Std Dev)
0	6082.662	2336.872	407.5376	128.7943
1	6115.067	2773.158	200.3974	395.0648
2	6445.974	3119.597	537.0887	1223.719
3	6851.437	3389.927	840.3333	622.6345
4	7479.038	3982.055	80.91919	216.4259
5	8352.982	4141.219	336.4865	522.0744
7	9909.662	4064.393	16.41468	536.432
9	11923.92	4258.316	1387.034	772.7932
11	14074.99	4274.921	1809.347	788.6356
13	16038.07	4401.558	2596.879	825.379
15	21612.98	4445.483	7270.891	1101.053
17	21157.72	4444.65	6632.421	879.8031
19	21057.63	4578.458	6005.09	953.2828
21	21755.47	4883.174	7132.669	1357.725
22	24289.24	4768.68	10092.5	1220.397
23	26629.43	4914.341	11274.72	1219.776
25	28708.99	5274.828	12438.76	1500.564
27	28015.88	5143.908	10519.14	1400.693
28	29189.56	5389.575	12691.26	1339.827
29	29608.51	5806.262	13090.31	1399.888
30	33035.21	5667.34	16600.21	1461.637

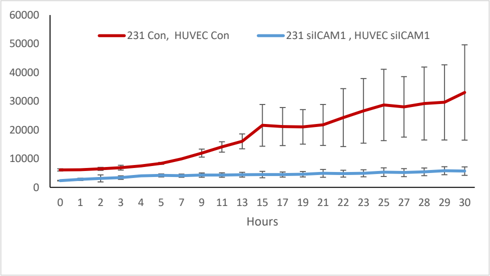

Fig 4f TN1- BLI Lung Metastasis

ICAM1 -	ICAM1 +
0.014604248	0.054165773
0.022973191	0.031653066
0.022576277	0.045108995
0.013320811	0.062488562

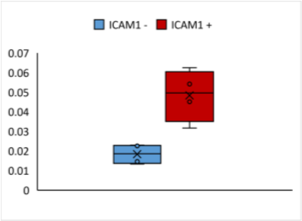

Fig 4h 231 anti-ICAM1- BLI Lung Metastasis

IgG	anti-ICAM1
0.222385638	0.119469457
0.262913236	0.172924433
0.237179596	0.149487824

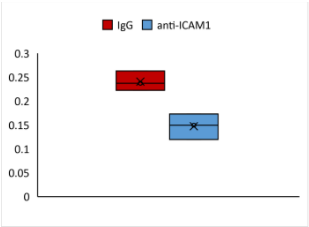

Fig 4i 231 anti-ICAM1- CTCs

IgG	anti-ICAM1
0.001	0.004
0.002	0.005
0.005	0.007

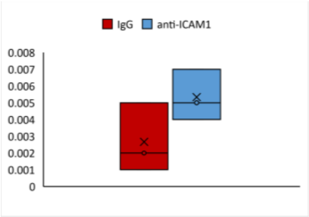

Figure 5 Source Data

Fig 5d 231 anti-ICAM1 aggregation

Hours	IgG	anti-ICAM1	IgG (Std Error)	anti-ICAM1 (Std Error)
0	5549.44	5427.845	1224.091	851.6711
1	8621.747	6393.455	1467.624	689.8127
2	10579.63	7136.274	1845.597	889.0221
4	10435.16	7027.778	1667.121	1077.82
6	11637.11	6284.472	2780.29	713.9831
8	14539.71	7106.873	3900.012	948.1987
9	15317.58	7975.35	3438.528	1166.738
10	19416.86	8117.873	4031.604	718.4599
12	23252.62	9271.74	6013.579	1150.226
14	28605.63	11072.15	7307.527	1769.576

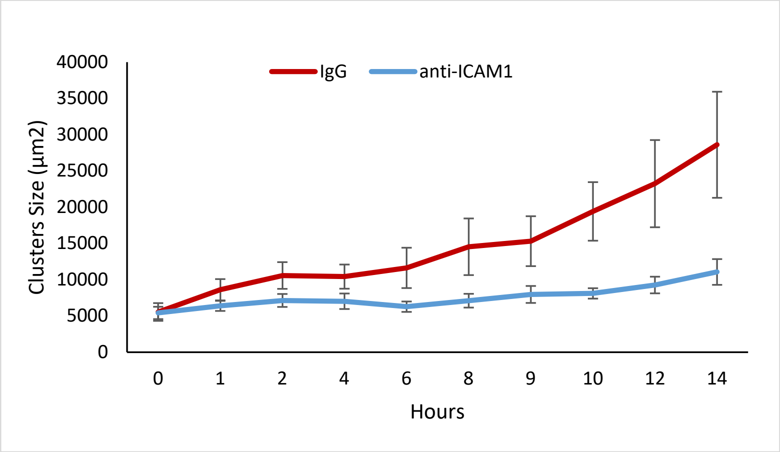

Fig 5f 231 anti-ICAM1 TEM

IgG	anti-ICAM1
14400	4200
8700	3600
14700	4500
9300	2100

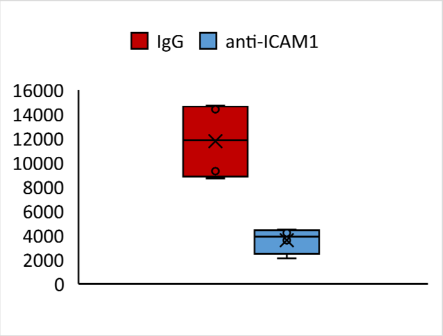

Fig 5h 231 anti-ICAM1 Spontaneous Lung Metastasis

IgG	anti-ICAM1
10615810.7	2484135.714
7746616.6	2410402.5
6935610	3344650

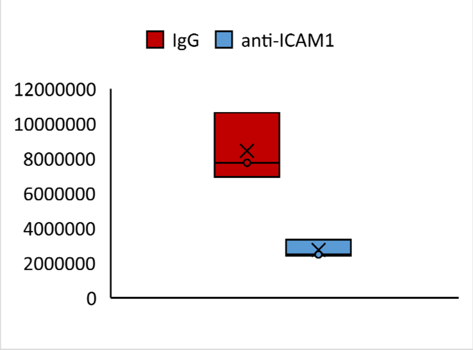

Supplementary Figure 3 Source Data

Supp. Fig 3a

CellSearch CTC patient	ICAM1+ cells
1	0.118421053
2	0.179586563
3	0.212518195
4	0.272727273
5	0.310344828
6	0.33853211
7	0.409274194
8	0.440217391
9	0.571428571

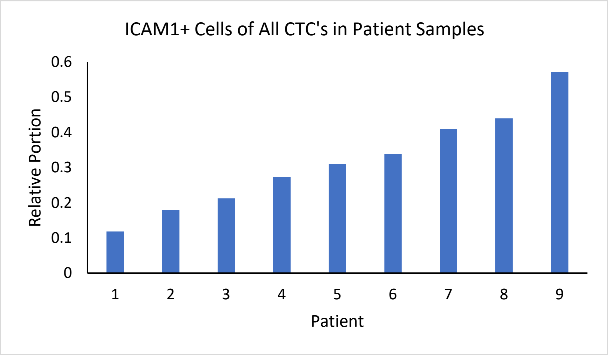

Supp. Fig 3b

CellSearch CTC

ICAM1+ Singles	ICAM1+ Clusters
0.150882825	0.302013423
0.359550562	0.414634146
0.270454545	0.45
0.394209354	0.666666667
0.443181818	0.75

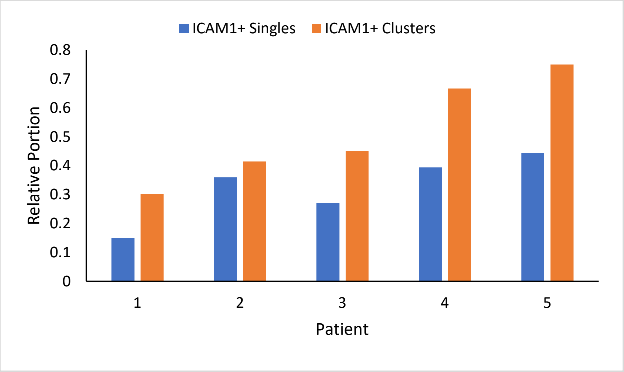

Supplementary Figure 4 Source Data

Supp. Fig 4a TN3 sorted aggregation

Hours	TN3 PDX ICAM1+	TN3 PDX ICAM1-	TN3 ICAM1+ (Std Dev)	TN3 ICAM1- (Std Dev)
1	2574.659	1176.283	489.1741	49.21133
2	4807.611	1585.267	1050.225	591.0876
3	5285.108	1588.586	1104.387	495.7609
4	4810.473	1496.84	983.5862	278.3916
6	4658.361	1469.619	987.3871	187.509
8	4582.139	1471.918	854.562	192.3494
10	4841.159	1529.313	1211.901	201.2453
12	4773.554	1518.573	1229.11	193.4563
14	4735.881	1567.344	1025.854	227.158
16	4679.477	1549.6	1114.713	178.0369
18	4461.723	1540.884	833.4722	159.7352
20	4365.051	1553.131	856.7463	181.8796
22	4372.499	1569.819	813.0834	207.6947
24	4565.148	1551.204	1084.827	181.7327

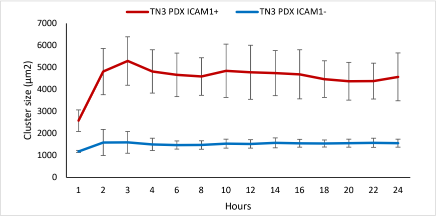

Supp. Fig 4b TN3 siICAM1 aggregation

Hours	siCon	siICAM1	siCon (Std Err)	siICAM1 (Std Err)
0	4280.66475	2461.667875	148.4167	95.09427
2	4750.107	2815.68375	168.454	124.2972
4	5394.18525	3005.061125	239.4051	103.5349
6	5953.35525	3263.157875	286.4602	122.3945
8	6478.9185	3661.13625	170.4269	148.8595
10	7451.087375	4185.178125	659.592	219.1309
12	9052.211375	4411.162	1311.563	218.0944

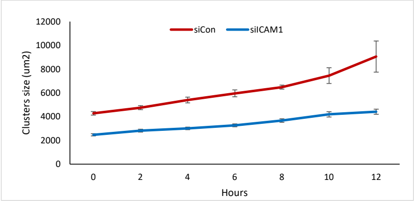

Supp. Fig 4e 231 siICAM1-siCD44 aggreation

Hours	siCon	siICAM1	siCD44	siCon (Std Err)	siICAM1 (Std Err)	siCD44 (Std Err)
0	2349.732	2805.323	3005.371	437.3912	263.3069	82.67647
1	4317.074	3852.865	4379.593	108.3164	127.83	164.2082
2	7222.198	4709.058	6223.77	225.3395	140.2775	224.6801
3	17695.11	6309.468	10574.89	1238.762	266.5724	487.6923
4	33624.67	7818.349	16261.4	3441.553	394.8413	822.2631
5	43504.52	9684.26	23851.05	3052.02	409.5486	1554.748
6	65358.66	12735.04	34414.8	7049.584	697.3925	2930.504
8	96276.38	17997.36	65636.81	11204.41	1295.142	9853.377
10	127767	30881.14	94771.45	13479.65	3471.46	14089.95
12	147000.4	39887.24	110182	24980.44	4289.782	18726.87

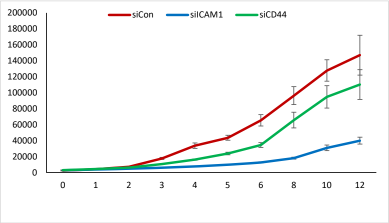

Supp. Fig 4f 231 sorted ICAM1-CD44 aggreation

Hours	ICAM1+/CD44+	ICAM1+/CD44-	ICAM1-/CD44+	ICAM1+/CD44+ (Std Dev)	ICAM1+/CD44- (Std Dev)	ICAM1-/CD44+ (Std Dev)
0	1239.609	435.3553	44.09368	651.9813	509.0635	62.35788
2	2371.979	280.5623	0	951.0353	363.1387	0
4	3265.686	360.9356	0	712.5248	481.856	0
6	4475.676	302.5161	0	1371.105	392.6228	0
8	4348.195	480.7513	0	1012.827	399.4409	0
10	3908.449	538.7988	0	872.6992	656.9166	0
12	4049.772	545.1244	0	934.3884	357.389	0
14	3484.703	472.9373	40.93084	835.0975	465.91	57.88495
16	3630.64	722.2433	62.14046	890.405	662.2372	87.87988
18	4169.141	705.871	0	992.4384	670.4192	0
20	4934.548	450.6114	163.7234	1517.316	353.5688	78.4078
22	6498.776	1218.623	294.8881	1682.987	978.0743	282.847

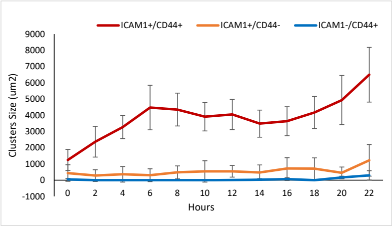

Supplementary Figure 7 Source Data

Supp. Fig 7b 231 ICAM1 Targets- Mammosphere

siCon	67	73	77
siCDK6	38	53	46
siSec23a	43	40	57
siZeb1	29	33	31
siICAM1	39	42	34

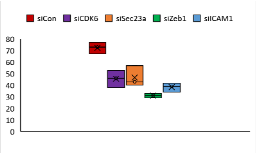

Supp. Fig 7f 231 ICAM1 Targets- Clustering

Hours	siCon	siICAM1	siCDK6	siSec23a	siZeb1	siCon (Std Err)	siICAM1 (Std Err)	siCDK6 (Std Err)	siSec23a (Std Err)	siZeb1 (Std Err)
0	3436.997	3154.617	3300.99	3371.088	2989.282	470.6985	263.267	67.03857	187.3692	75.63711
2	4360.631	3557.592	5686.564	4133.271	3525.744	83.22063	105.871	207.8756	145.1611	93.92319
4	8627.543	4666.538	11819.25	6637.632	5404.232	320.0208	127.262	349.4136	286.2548	141.5246
6	16034.7	6104.704	20428.63	8697.621	9134.739	1083.226	237.5637	1024.3	430.7331	441.9329
8	24208.63	7843.595	26515.57	11797.29	13497.3	1531.498	511.3268	1950.815	897.3362	818.4231
10	33156.86	8815.676	34038.77	15823.3	15693.29	2891.486	549.91	1869.298	930.3239	1112.58
12	44247	9482.393	45731.29	18257.73	17016.51	3355.789	523.5018	3444.781	1119.36	1379.503
14	52064.65	9889.433	52943.1	24029.82	17947.79	3083.429	586.7422	5803.319	2077.457	1273.523
16	54892.16	9842.09	55435.34	26628.7	18693.38	2742.242	592.3599	7595.382	1745.12	1426.656
18	61327.04	10062.26	54102.41	30705.3	17906.83	3108.126	576.6551	3613.625	2392.868	1383.784
20	62398.83	10424.07	58048.04	36680.51	17979.48	2331.104	759.4388	4317.468	4302.082	1078.193

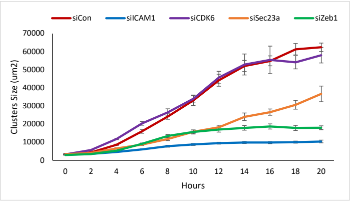

Supp. Fig 7h 231 ICAM1 Targets- BLI Lung Metastasis

		Day 0	Day 1	Day 6	Day 7- lungs
Normalized c	siCon	1	0.46190781	0.95617443	6.08175166
		1	0.4884111	0.86254794	2.78633303
		1	0.41432555	1.10198074	5.4786357
	siSec231	1	0.81541851	0.84311936	5.64522763
		1	0.76362539	1.179561	5.55521346
		1	0.5017808	0.61724736	2.46768951
	siZeb1	1	0.98839728	1.15452002	8.54369408
		1	0.65250922	0.90532824	6.75039526
	siICAM1	1	0.19706929	0.28357666	0.7813712
		1	0.2227191	0.25873886	0.48766357
		1	0.14489275	0.14817428	0.62474464

		0	1	6
		Day 0	Day 1	Day 6
Averages:	siCon	1	0.454881488	0.9735677
	siSec231	1	0.693608231	0.87997591
	siZeb1	1	0.820453249	1.02992413
	siICAM1	1	0.188227047	0.23016327

		Day 0	Day 1	Day 6
SD:	siCon	0	0.037539233	0.12066031
	siSec231	0	0.168133743	0.28296283
	siZeb1	0	0.23750872	0.1762052
	siICAM1	0	0.039659476	0.07208241

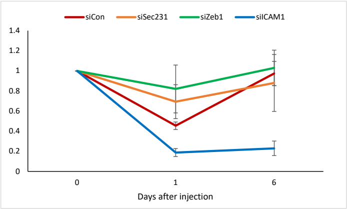

Supp. Fig 7i 231 ICAM1 Targets- BLI Lung Metastasis at day 7

	Day 7- lungs		
siCon	6.08175166	2.78633303	5.4786357
siSec231	5.64522763	5.55521346	2.46768951
siZeb1	8.54369408	6.75039526	
siICAM1	0.7813712	0.48766357	0.62474464

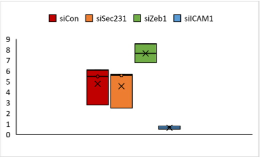

Supp. Fig 7j 231 ICAM1 Targets- Cell Cycle

Cell cycle %	siCon	siICAM1	siCDK6
Apoptosis	3.5	3.2	0.8
	4.9	2.6	1.6
	4.9	6.2	1.9
G0/G1	55.2	54	59.5
	53.4	51.6	60.5
	56.4	53.3	63.1
S	15.7	13.7	13.3
	14.1	14.9	14.9
	13.9	13.9	13.6
G2/M	24.6	29	26.4
	26.4	30.5	22.8
	24.3	26.2	21.5

Average

	siCon	siICAM1	siCDK6
Apoptosis	4.43333333	4	1.43333333
G0/G1	55	52.9666667	61.0333333
S	14.5666667	14.1666667	13.9333333
G2/M	25.1	28.5666667	23.5666667

SD	siCon	siICAM1	siCDK6
Apoptosis	0.80829038	1.92873015	0.56862407
G0/G1	1.50996689	1.23423391	1.85831465
S	0.98657657	0.64291005	0.85049005
G2/M	1.13578167	2.1825062	2.53837218

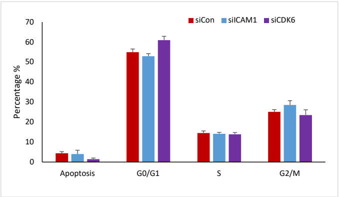

Supplementary Figure 8 Source Data

Supp. Fig 8b 231 siICAM1-siCDK6 BLI Lung Metastasis

siCon	0.32392284	0.51205147	0.38002314
siICAM1	0.111722022	0.118344113	0.110805341
siCDK6	0.30834558	0.41655929	0.47351564

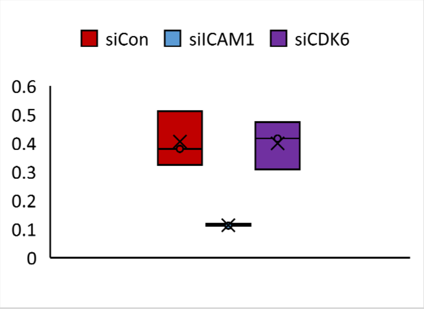

231 siICAM1-siCDK6 CTC

siCon	0.00096	0.00075	0.00109
siICAM1	0.0009	0.00143	0.00258
siCDK6	0.00129	0.00005	0.00136

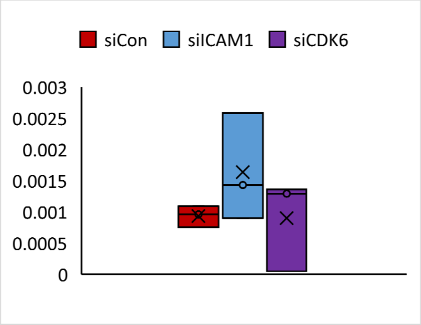

Supplementary Figure 9 Source Data

Supp. Fig 9c 231- ICAM1 siRNAs- BLI Lung Metastasis Day 0

			Day 1	Day 3	Day 6
Averages:	siCon	1	0.301661435	0.443815698	1.090325041
	siICAM1- SP	1	0.130091857	0.128032299	0.214195222
	siICAM1- A	1	0.18228931	0.164762887	0.274640732
	siICAM1- B	1	0.233380491	0.234988383	0.348897327
		Day 0	Day 1	Day 3	Day 6
SD	siCon	0	0.118016189	0.129361695	0.369819389
	siICAM1- SP	0	0.022004158	0.021391474	0.063806164
	siICAM1- A	0	0.058214373	0.056390776	0.10308542
	siICAM1- B	0	0.05978584	0.024992461	0.1005748
	days	0	1	3	6

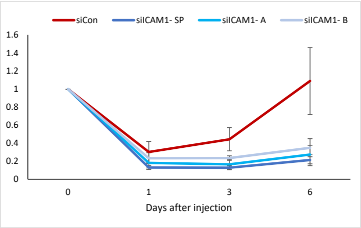

Supp. Fig 9d 231- ICAM1 siRNAs- BLI Lung Metastasis at Day 6

siCon	siICAM1- SP	siICAM1- A	siICAM1- B
3.28515508	0.387209671	0.889738502	0.854150999
0.99241669	0.431394871	1.258397699	1.081175488
2.44436251	0.523565956	0.646840117	0.599283825
1.82904763	0.33380341	0.278404088	0.984352664

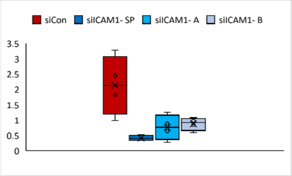

Supp. Fig 9f 231- ICAM1 siRNAs- Clustering

Hours	siCon	siICAM1-SP	siICAM1-A	siICAM1-B	siCon (Std Dev)	siICAM1-SP (Std Dev)	siICAM1-A (Std Dev)	siICAM1-B (Std Dev)
0	28479.34	10128.79	8120.58	5237.319	5338.923	1239.762	2139.196	1005.97
1	120476.9	23959.92	17660.74	14125.8	45675.49	2883.435	2685.807	1286.792
2	186698	43922.94	32943.6	30061.99	56731.23	7090.771	6661.626	8726.254
3	195829.7	48358.77	42210.7	46079.88	99569.02	7573.986	5471.761	9515.376
5	217522.4	47005.92	60725.69	50397.63	100966.2	6892.203	19631.04	4647.183
7	228050.4	45767.3	78230.59	60572.8	91955.88	7242.573	36757.72	14362.06
11	272357	51813.01	119921	72886.53	100221.6	7747.225	29761.48	28634.7

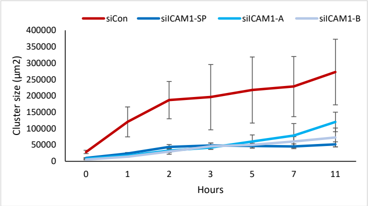

Supp. Fig 9h 231- ICAM1 siRNAs- Mammosphere

siCon	86	90	71
siICAM1- SP	38	50	44
siICAM1- A	39	49	44
siICAM1-B	34	36	51

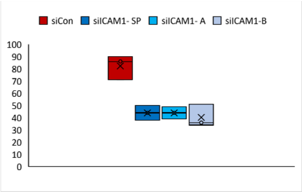

Supp. Fig 9j 231- ICAM1 siRNAs- Proliferation

siCon	60	52	70
siICAM1- SP	23	27	23
siICAM1- A	27	23	20
siICAM1-B	21	18	32

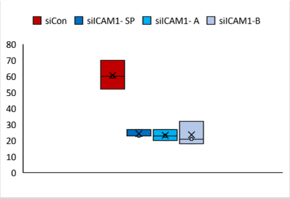

Supplementary Figure 10 Source Data

Supp. Fig 10d 231 anti-ICAM1- BLI Lung Metastasis

IgG	anti-ICAM1
1.24	0.66
1.00	0.77
0.83	0.57

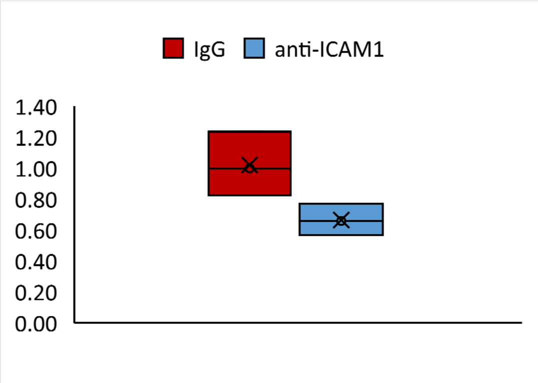

Western Blots

Figure 2j

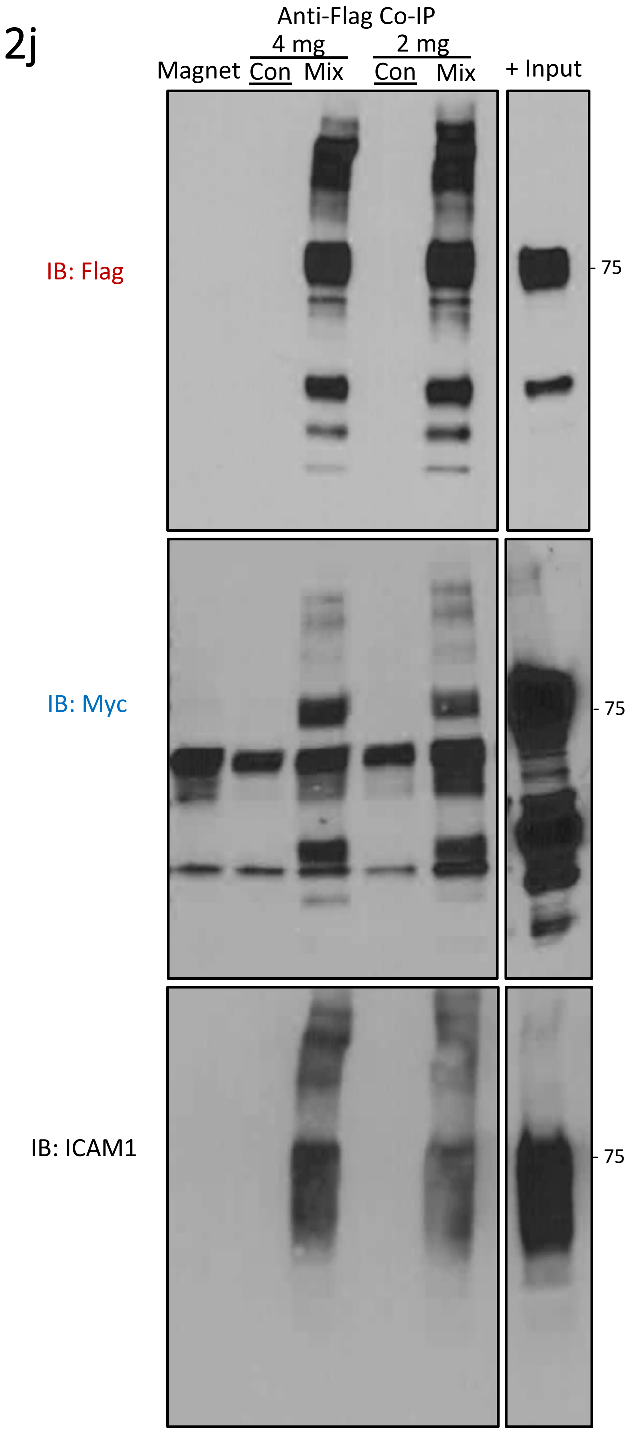

Figure 2l

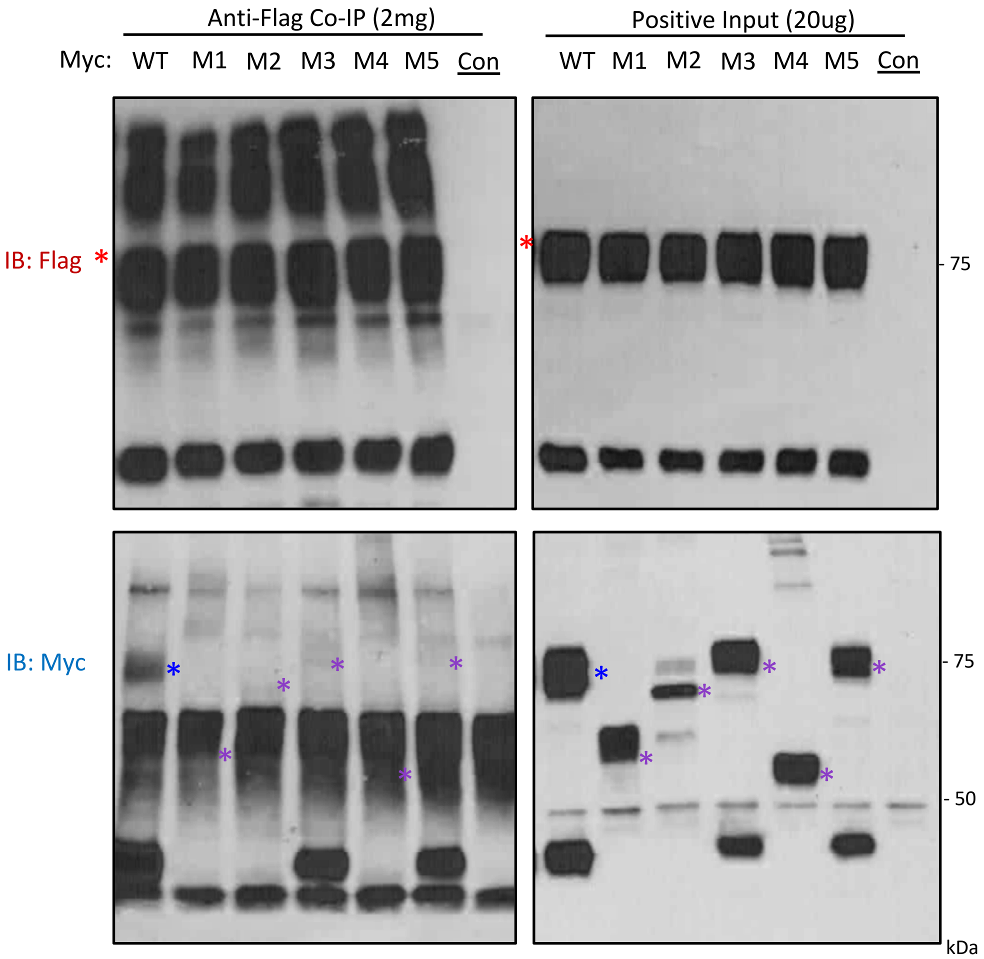

Figure 3c

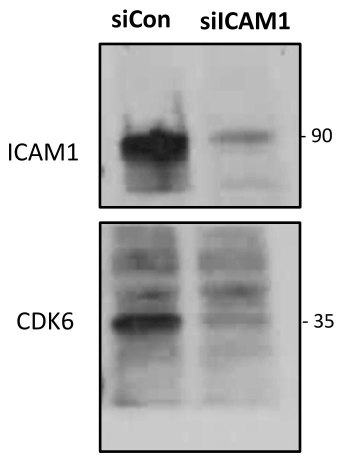

Suppl. Figure 1f

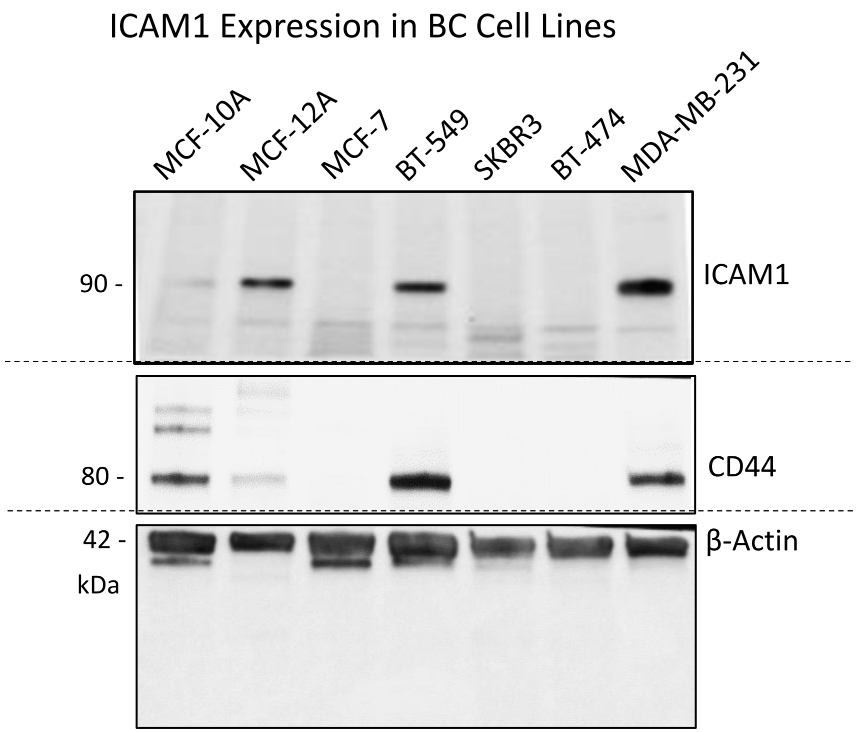

Suppl. Figure 3f

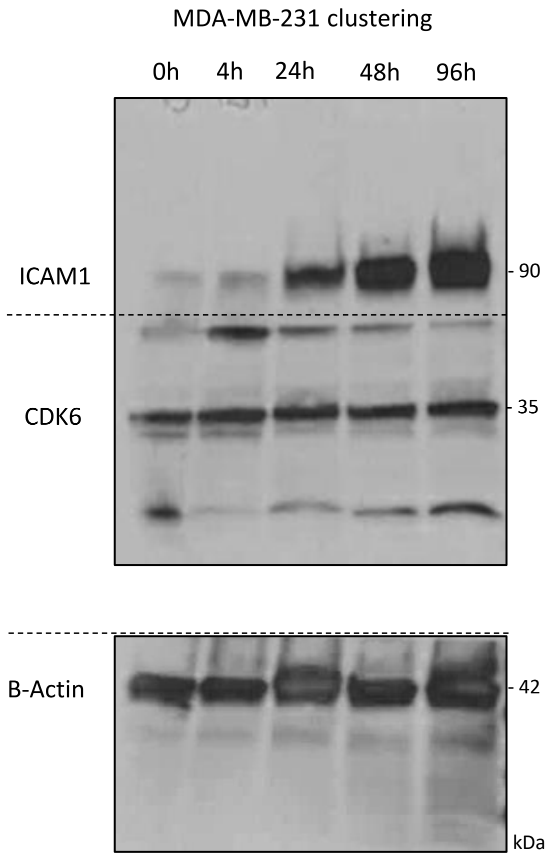

Suppl. Figure 4c

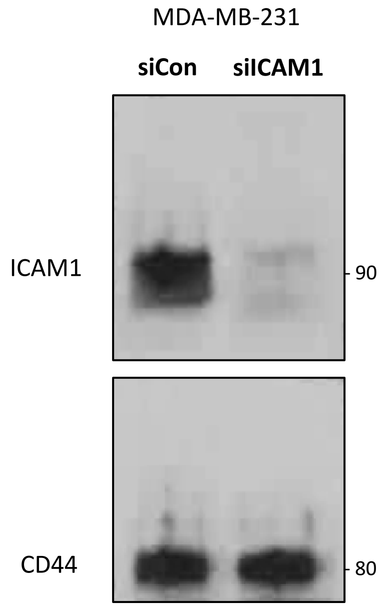

Suppl. Figure 5b

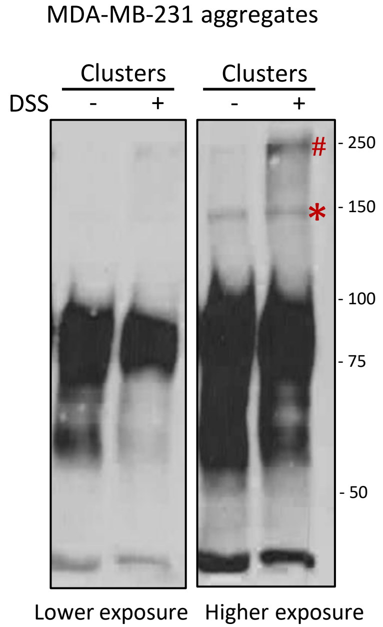

Suppl. Figure 6c,f

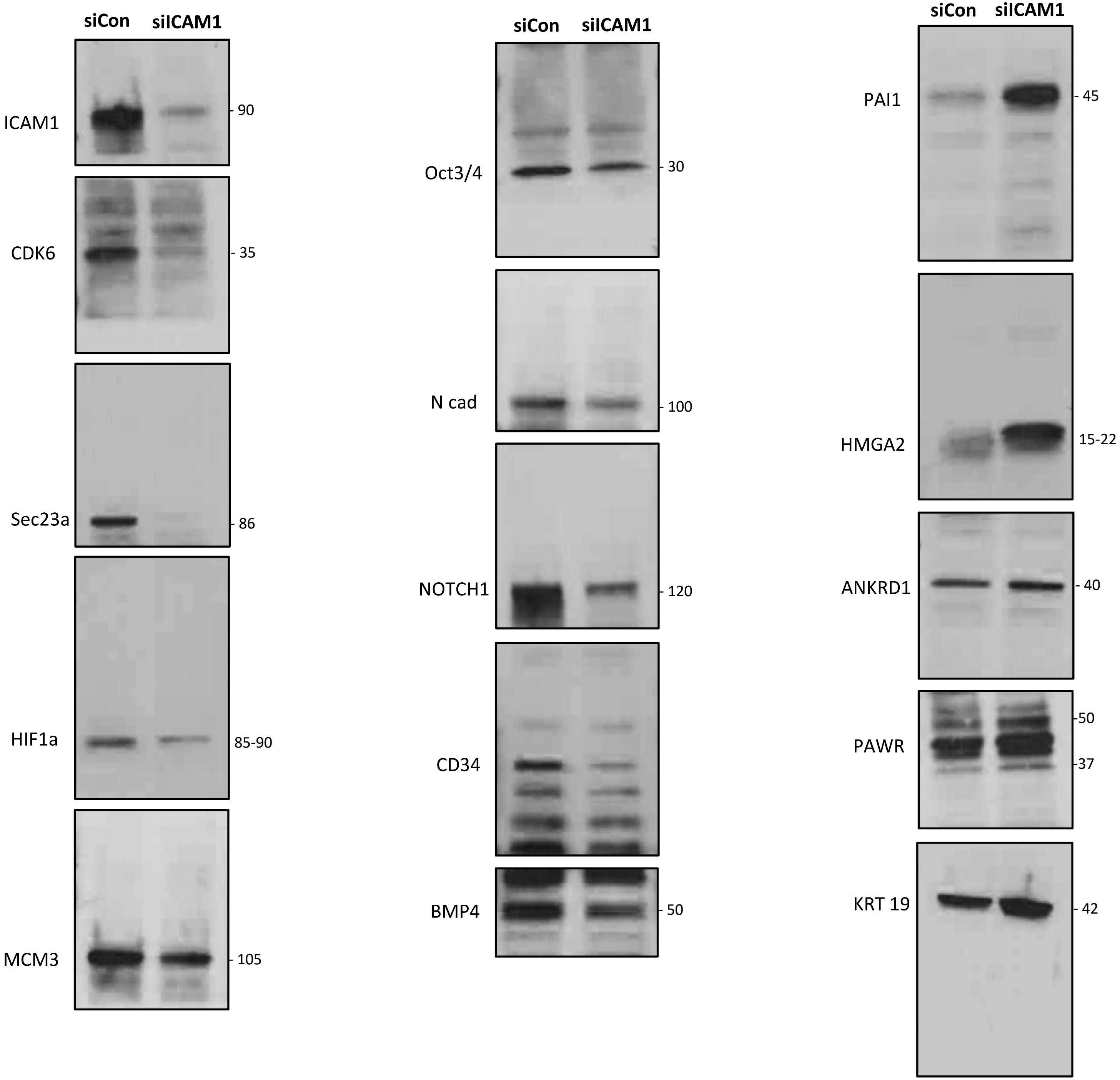

Suppl. Figure 7a

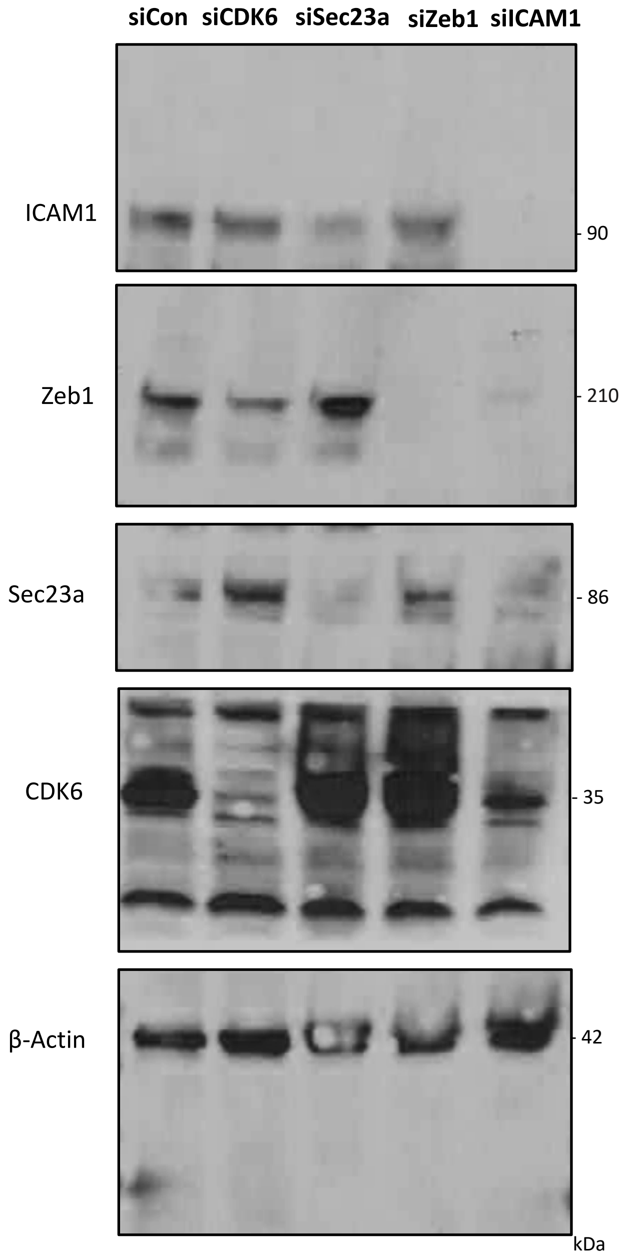

Suppl. Figure 8c

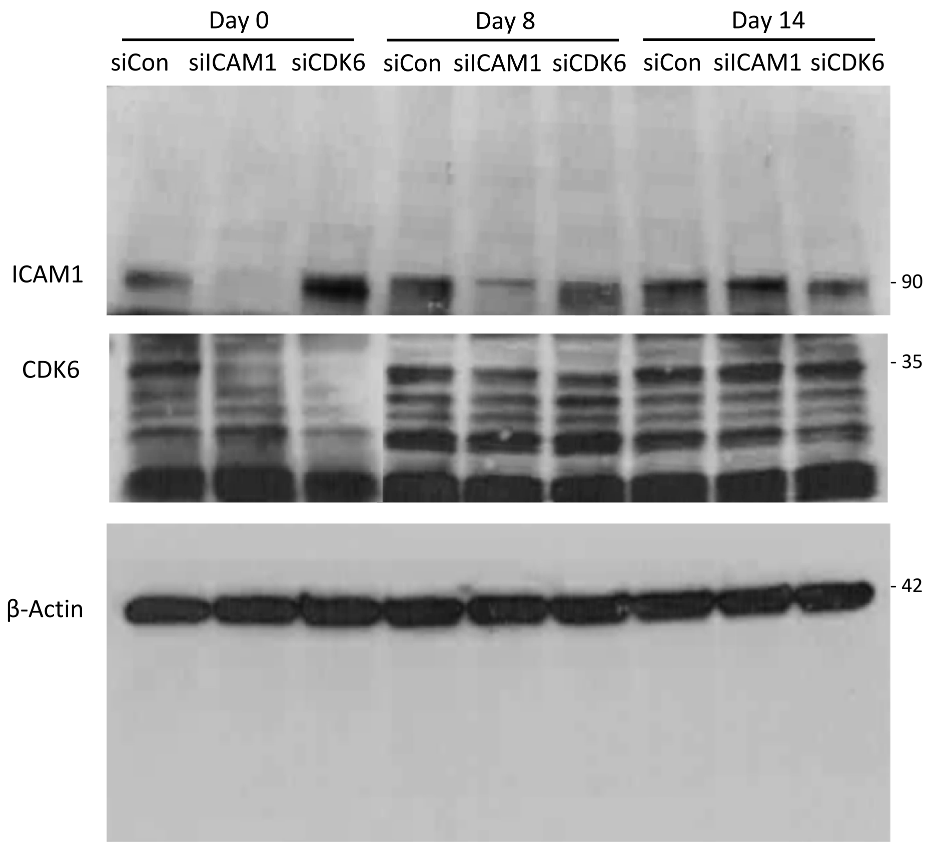

Suppl. Figure 9a

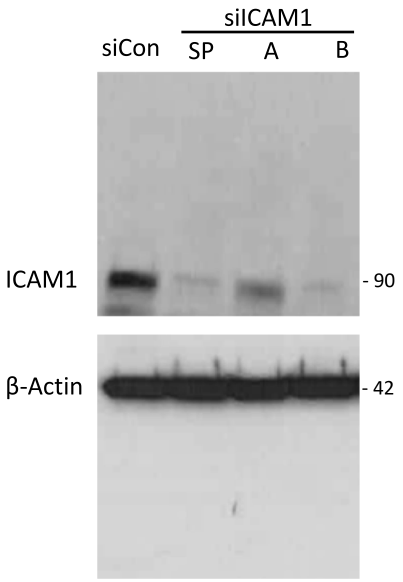

Supplement: Supplementary file 3 — Source Data [file 41467_2021_25189_MOESM3_ESM.pdf]
